# Molecular signatures written in bone proteins of 79 AD victims from Herculaneum and Pompeii

**DOI:** 10.1038/s41598-022-12042-6

**Published:** 2022-05-27

**Authors:** Georgia Ntasi, Ismael Rodriguez Palomo, Gennaro Marino, Fabrizio Dal Piaz, Enrico Cappellini, Leila Birolo, Pierpaolo Petrone

**Affiliations:** 1 Department of Chemical Sciences, University of Naples Federico II, Naples, Italy; 2 Evolutionary Genomics Section, Globe Institute, University of Copenhagen, Copenhagen, Denmark; 3 Department of Humanities, University Suor Orsola Benincasa, Naples, Italy; 4 Department of Medicine, Surgery and Dentistry, University of Salerno, Fisciano, Salerno, Italy; 5 Task Force Di Ateneo “Metodologie Analitiche per la Salvaguardia dei Beni Culturali”, University of Naples Federico II, Naples, Italy; 6 Department of Advanced Biomedical Sciences, Departmental Section of Legal Medicine, Anatomy and Histology, University of Naples Federico II, Naples, Italy

**Keywords:** Analytical chemistry, Biochemistry, Proteins, Proteomics

## Abstract

An extensive proteomic analysis was performed on a set of 12 bones of human victims of the eruption that in AD 79 rapidly buried Pompeii and Herculaneum, allowing the detection of molecular signatures imprinted in the surviving protein components. Bone collagen survived the heat of the eruption, bearing a piece of individual biological history encoded in chemical modifications. Here we show that the human bone proteomes from Pompeii are more degraded than those from the inhabitants of Herculaneum, despite the latter were exposed to temperatures much higher than those experienced in Pompeii. The analysis of the specimens from Pompeii shows lower content of non-collagenous proteins, higher deamidation level and higher extent of collagen modification. In Pompeii, the slow decomposition of victims’ soft tissues in the natural dry–wet hydrogeological soil cycles damaged their bone proteome more than what was experienced at Herculaneum by the rapid vanishing of body tissues from intense heat, under the environmental condition of a permanent waterlogged burial context. Results herein presented are the first proteomic analyses of bones exposed to eruptive conditions, but also delivered encouraging results for potential biomarkers that might also impact future development of forensic bone proteomics.

## Introduction

Ancient protein analysis provides clues about human and animal life and diseases from the past^1^. Bone, a mineralised extracellular matrix rich in connective tissue components, is one of the most abundant sources of ancient proteins and one of the most abundant biomaterials in the archaeological record. The diagenesis, in particular when heat-induced, of organic matter in archaeological bone, initially represented mostly by type I collagen (90%), lipoproteins and mucopolysaccharides^2^, is a complex phenomenon^3,4^, linked to several factors in the depositional environment^2,5,6^. The analysis of how diagenesis acted to produce or prevent specific modifications led to coin the term “diagenetiforms” to describe the variety of distinct molecular species arising from chemical modifications by environmental conditions^7^.

Mass spectrometry-based techniques have been widely applied to characterize collagen in ancient proteomes and its chemical modifications occurred pre- and post-mortem^8^. Apart from the extensive observation of deamidation of asparagines and glutamines Gln^9–12^ and methionine oxidation^13^, a few other diagenetically induced modifications were also detected, such as: (i) aminoadipic acid formation from lysine, (ii) tryptophan oxidation products^13^, (iii) advanced glycation end-product (AGEs)^13–15^, (vi) backbone cleavage^14,15^, while complex oxidative reactions occurring on prolines have been hypothesised but not characterised in details^13,14^. So far though, no study characterized the modifications occurred post-mortem in the proteome of archaeological bones from individuals who died due to the nearly instantaneous exposure to extreme temperature.

Here we applied a bottom-up proteomic approach to investigate the proteome and the chemical modifications present in the bone proteins of humans from Herculaneum^16^ and Pompeii (Italy)^17^, who died during the eruption of the Vesuvius in 79 AD. The accurate description of the catastrophic event affecting Herculaneum and Pompeii provided by ancient sources, and the peculiar burial conditions^18–20^, offer a unique opportunity to analyse lifeways in a relatively large cohort of perfectly coeval individuals who lived and died together^21^, whose bone proteome experienced such extreme conditions.

Herculaneum, Pompeii and other Roman settlements up to 20 kms away from the volcano were suddenly hit by successive hot pyroclastic currents and buried by up to tens of meters thick volcanic ash deposits produced by the 79 AD eruption, that killed everyone who had not been evacuated or managed to flee^16^. The skeletal remains were in an excellent state of preservation as a result of the unusual death and burial conditions: instant death caused by hot pyroclastic surges at temperatures between approx. 300 (Pompeii) and 500 °C (Herculaneum)^16,17,22^.

In Herculaneum, death was followed by vanishing of soft tissues and rapid replacement by volcanic ash^19^. Here, some evidence suggests that the volcanic environment was characterised by a drop in temperature of the first pyroclastic surge during its emplacement. Evidence of rapid cooling of the volcanic ash cloud may account for the preservation of organic tissue residues^22,23^ and organic compounds^24^. At Herculaneum the ash-bed deposit was permanently waterlogged by groundwater^25,26^, as revealed by the early 1980s archaeological investigations of the victims' skeletons on the ancient beach, thus a permanent system of hydraulic pumps was activated^20^. The burial environment of the victims was most likely able to inhibit microbial attack to bone and related diagenetic processes^27^.

On the other hand, the temperature of approx. 300 °C experienced by the victims in Pompeii was sufficient as well to instantly kill people, but it was not hot enough to cause rapid soft tissue disappearing as in the case of Herculaneum. Therefore, in Pompeii the victims’ corpses were preserved intact inside the ash deposit after its rapid cooling and hardening around them^16^. The cavity formed around the victim's body after the slow disappearance of the flesh would then be filled with plaster of Paris in order to obtain plaster casts, technique adopted for the first time on human victims in 1863 by Giuseppe Fiorelli^28^. Over the last century and up to the present, in the case of bodies of victims found in the ash surge deposit, this technique has been used to replicate the features of the body.

In this paper, our objective was a proteomic profiling of the bones of the eruption victims, using a bottom-up proteomic approach and an unbiased discovery of chemically modified peptides^29^, in search for signatures of the high temperatures and environmental conditions the bodies were exposed to. Bone collagen survived even the harshest conditions of temperature imposed by the volcano eruption, bearing encoded in the chemical modifications a piece of individual biological history. For comparison, the bones from a coeval skeletal population from the Campanian region were considered (Baia, Scalandrone locality, II sec. AD, Roman Imperial Age, Puteoli, Naples, Italy). The necropolis site of Baia Scalandrone was chosen as a control because it is coeval with the sites of Herculaneum and Pompeii and, as with these, the burial ground is of volcanic origin. Furthermore, unlike the two Vesuvian sites, the bodies of these individuals were not exposed to heat.

Most interestingly, bones are frequently found in archaeological and forensic contexts, and their characterization for the study of past populations (e.g., age at death or details of funerary practices), or for victim characterization in forensic investigations is of unquestionable relevance^30–33^. Actually, burned bones might be the only remains found in forensic scenarios (e.g. from terrorist attacks, explosions or fires) from which identify victims or obtain information, and the present study is expected to contribute to a full molecular characterisation of bones that have been exposed to heat.

## Results

### Proteome of human bones

A shotgun proteomics approach by LC–MS/MS (Figure S2) was applied to skeletal samples excavated from the archaeological sites of Pompeii (7 samples) and Herculaneum (5 samples)^16,19^. Samples collected from three individuals from Roman Imperial Age (II sec. AD) cemetery in Baia Scalandrone were also analysed to compare results from the AD 79 eruption victims with this coeval skeletal population. All specimens are illustrated in the Table S1 and Figure S1. Moreover, raw data from proteomic analyses of archaeological bones from a completely independent excavation site, from the Hitotsubashi site (AD 1657–1683) in Tokyo, Japan, were used for comparative evaluation of the results. Samples H-162, H-142 from^34^ were selected because proteins have been extracted with almost the same protocol and analysed on the same instrument as herein samples.

Very stringent criteria for protein identification were used: only peptides with score higher than 70 were considered and proteins were considered as identified only when 2 or more peptides have been detected (Table S2).

The number of identified proteins in the samples from Herculaneum, Pompeii and Baia Scalandrone varied among individuals from 2 to 27 and includes collagenous and non-collagenous proteins (NCPs) (Table S3). The expected dominance of collagen in bone tissue is reflected by the result that the two chains of type 1 collagen, namely collagen alpha-1 (I) and collagen alpha-2 (I), were confidently identified in all the samples. Of the 15 samples, 5 contained only type 1 collagen chains (four samples out of seven from Pompeii, 2.8 proteins on average, ± 1.12, and one sample from Baia Scalandrone) while samples from Herculaneum exhibited a higher protein content (13 proteins on average, ± 7). Bone proteome complexity is affected by several factors, including burial age^35^. In this case, since bones from Pompeii and Herculaneum are exactly coeval, the volcanic environmental conditions during death and burial appear to have played a significant role in protein survival in the two different sets of bone samples^36^. Raw data of Control samples were processed with the same constrains as the samples herein analysed, and a large number of proteins, (38 ± 0) were identified, as already reported^34^.

Despite a general large variability among the individuals within the different groups (Figure S3), samples can be grouped depending on the site of origin in respect to NCP content. We definitively observed that bone samples from Herculaneum exhibit more NCPs than those from Pompeii. Venn diagram using the ensembles of the proteins identified in each sample group shows that the proteins identified in Pompeii bones are common to all three groups, while several other proteins are shared exclusively by Herculaneum and Baia Scalandrone bones (Figure S4, Table S4).

The non-collagenous proteins identified in this study agree with those expected for archaeological bones^37,38^. Most of the identified NCP proteins are small leucine-rich proteoglycans (SLRPs) from the extracellular matrix (namely chondroadherin, biglycan, decorin, lumican and osteomodulin), all involved in biomineralisation or interacting with fibrillar collagen, (such as vitronectin and pigment epithelium-derived factor). Moreover, alpha-2-HS-glycoprotein, also known as fetuin-A is a bone matrix protein, known to have a high affinity for apatite. It is worth mentioning that several of the NCPs are related to the coagulation pathway (namely, prothrombin and antithrombin III, and Protein Z-dependent protease inhibitor), and can be functionally connected (Figure S5).

### Diagenetically induced modifications in bone proteins

Modifications of amino acids, such as oxidation of methionine, deamidation of asparagine and glutamine, as well as the backbone cleavage, are all degradation phenomena commonly observed and routinely searched for in ancient/aged proteins^13,39^.

#### Deamidation

**Figure 1:**
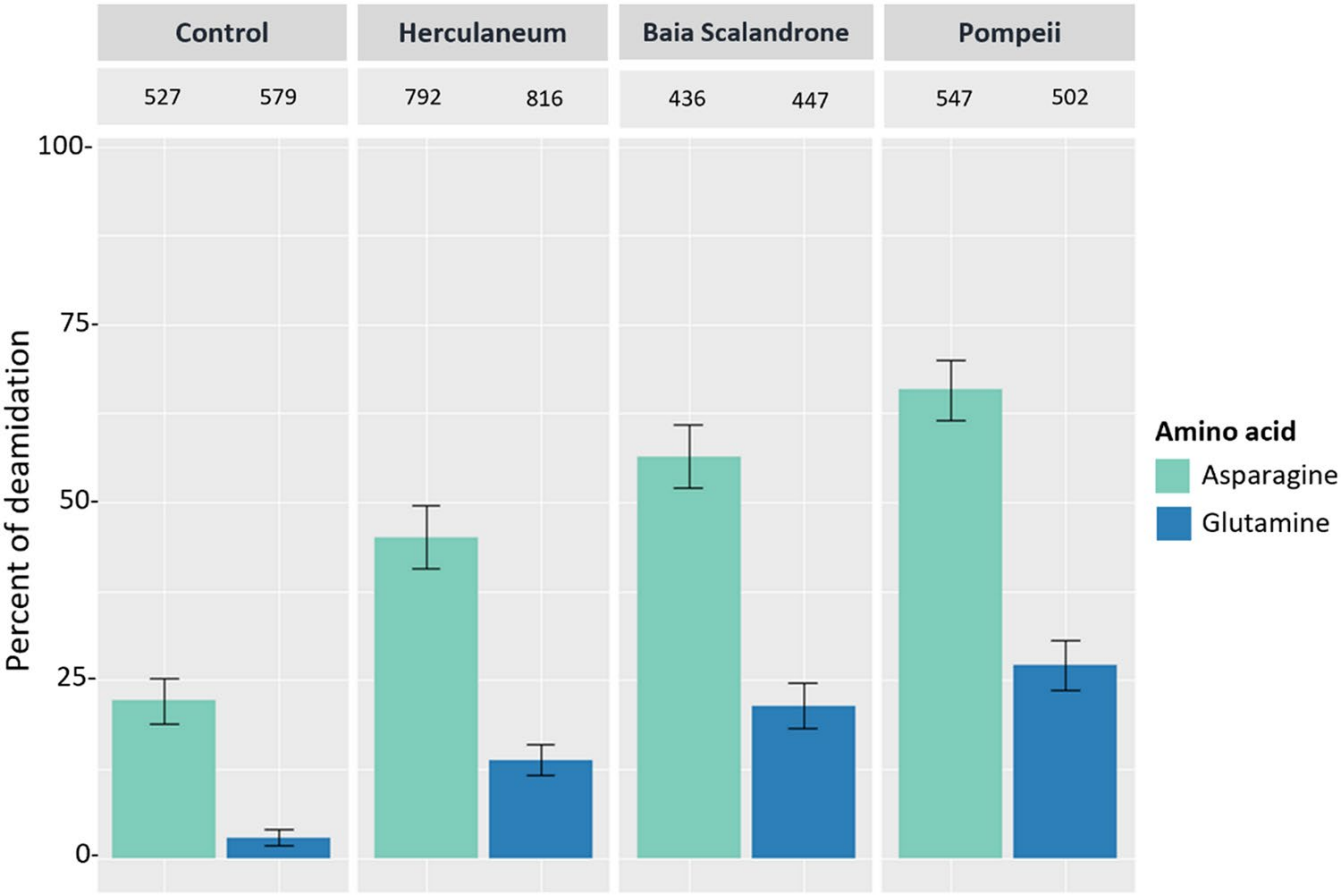
Overall percentage of deamidation for asparagine and glutamine residues of collagenous bone proteins from Pompeii, Herculaneum, Baia Scalandrone and control (H-162, H-142 from^34^). Error bars represent standard deviation and numbers above each bar represent the number of deamidation sites the data is based on.

To begin with, deamidation of asparagine (N) and glutamine (Q) residues, among the most common and most informative diagenetically derived modifications in proteins^11,12,40^, was examined. While extensive deamidation increases heterogeneity of the samples, it is a general and relevant glance on the myriad changes that archaeological bone proteins undergo and is influenced by the age and more generally by the preservation state of the bone sample under consideration^11,40–42^. Extensive protein deamidation (N,Q) has been consistently observed in ancient samples, and it has been routinely measured as part of the palaeoproteomics analysis of archaeological and paleontological specimens as a global indicator of sample preservation quality, since rates and levels of deamidation are affected by several chemical and environmental factors^40^. As expected, proteins in our samples are extensively deamidated and asparagine sites are much more deamidated than glutamines^40,43^, (Fig. 1). Moreover, we split the evaluation of the deamidation levels for collagenous (Fig. 1) and non-collagenous proteins (Figure S6) and, in agreement to what already reported by^15^, peptides from non-collagenous proteins showed very high to complete deamidation in comparison to peptides derived from collagenous proteins. On average, peptides from Pompeii samples are the most deamidated (see bulk deamidation per archaeological site, Fig. 1).

It is worth mentioning that Control samples are significantly more recent (1657–1683 AD)^34^ than samples from the Vesuvius area, and they are definitively less deamidated than Pompeii and Herculaneum bone samples. Variations were observed from individual to individual in the three groups from the Vesuvius area (Figure S7–S8). As a general trend, we can confidently assert that the lower the number of surviving proteins the higher the deamidation level, and it is worth observing that the few NCPs identified in samples from Pompeii are almost completely deamidated. Apparently, other factors rather than temperature might have played the biggest role in deamidation. In fact, the skeletal remains from Baia Scalandrone, which were not exposed to hot pyroclastic flows, but had been buried in the volcanic soil of the Campi Flegrei area (Bay of Puteoli, Gulf of Naples, Italy), exhibited a level of deamidation only slightly lower than that measured for bones from Herculaneum and Pompeii.

We analysed the distribution of deamidation level along the sequence of collagen type I chains, to explore the possibility of hot spots for deamidation rather than an average distribution. Figure S9 illustrates the deamidation values at single deamidation sites along collagen alpha-1 (I) and alpha-2 (I) chains. The label size indicates the relative intensity of each position in each sample. The values for Control are always well below those calculated for samples from the volcanic areas, in agreement with the global deamidation level calculated in Fig. 1. This difference is even more evident in glutamines, conceivably because glutamine deamidates more slowly. There is a trend in the deamidation; there are some zones where deamidation is more pronounced than others. This trend is almost reproducible in the samples of Pompeii, Herculaneum and Baia Scalandrone suggesting that the deamidation profile is quite robust for samples similar as concerns age and burial soil and also that three-dimensional arrangement might affect the local deamidation level.

#### Oxidation of methionines

With the same approach the oxidation of methionines (M) was evaluated. Figures S10–S11 illustrate the global oxidation levels of collagenous and non-collagenous proteins in all the samples. Apart from the zero values of control samples, all the proteins in all groups are almost totally oxidized (100%), demonstrating that methionine oxidation follows another pattern than deamidation. Furthermore, we investigate the oxidation values at single oxidation sites along collagen alpha-1 (I) and alpha-2 (I) chains. Almost all oxidation values are either 1 or 0, meaning that methionines are either fully oxidized or not oxidized at all (Figure S12). However, it is worth saying that several methionines were not detected, despite the generally good protein sequence coverage.

#### Backbone cleavage

Backbone cleavage of the polypeptide chain is also expected as a degradation feature in ancient proteins^15,44,45^, and can be evaluated since, upon trypsin hydrolysis, semi-tryptic peptides will be generated. Search for semi-tryptic peptides was carried out only on collagen type I chains for comparative purposes, since they are the only polypeptide chains shared among all the samples. The frequency of semitryptic peptides was evaluated as percentage of semitryptic peptides over the total number of identified peptides for each chain, on the basis of spectrum matches (PSMs).

**Figure 2:**
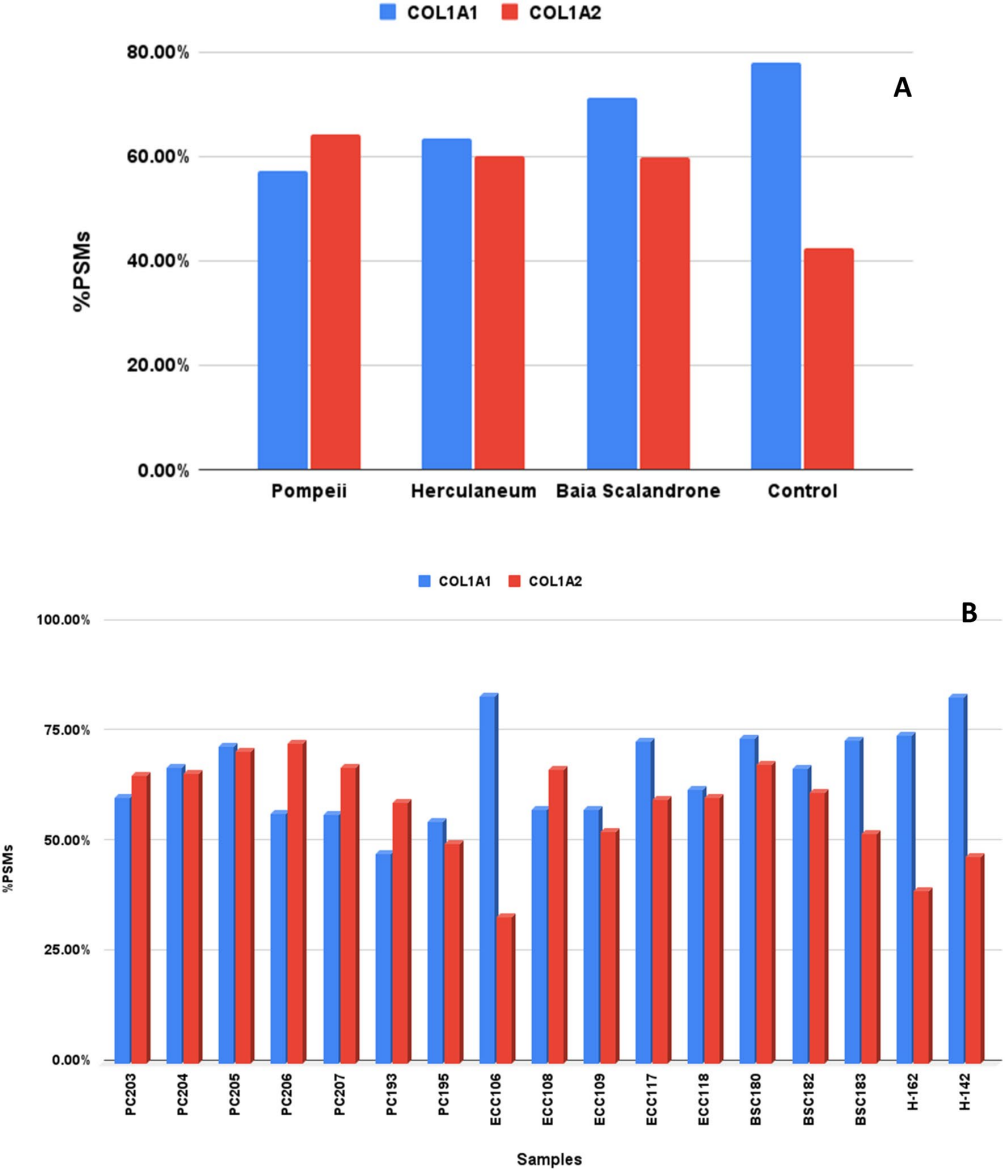
Backbone cleavages in collagen alpha 1(I) and collagen alpha 2 (I) in bone samples from Pompeii, Herculaneum, Baia Scalandrone and control (H-162, H-142^34^). Overall occurrence per samples groups (**A**) and in the single samples (**B**), evaluated as percentage of peptide-spectrum matches (PSMs) of semitryptic peptides over the total number of peptide-spectrum matches (tryptic plus semitryptic peptides).

Figure 2 shows the relative abundance of peptide-spectrum matches (PSMs) of semitryptic peptides over the total number of peptides of collagen alpha-1 (I) and alpha-2 (I) chains as a bulk per archaeological site (Fig. 2A) and in the single samples (Fig. 2B). The frequency of backbone cleavages is generally high. However, no clear-cut difference was observed among the samples from the volcanic areas or with the control sample.

The peptides showed a clear pattern derived from extended terminal hydrolysis occurring in regions of the collagen chains rather than in specific peptide bonds (Figure S13). A manual alignment of all the semitryptic peptides in the four different groups (Pompeii, Herculaneum, Baia Scalandrone, Control) to COL1A1 and COL1A2 sequences, however, reveals that while in case of controls the number cleavages are spread along the sequences, in the samples of Pompeii, Herculaneum and Baia Scalandrone they are localized in some regions of the protein sequences. These hot spots are between 266–286, 481–511, 772–796 and 1051–1118 sites of COL1A1 with a window of ± 2 amino acids, and in COL1A2 between 154–167, 232–250, 320–340, 425–438, 499–517, 681–708, 965–1006 and 1042–1066 with a window of ± 2 amino acids (Figure S14).

The cleavage frequency was then re-evaluated considering the regions rather than the single peptide bonds, by calculating the number of PSMs with semitryptic cleavages identified in a region divided by the total PSMs in the same region, including both tryptic and semitryptic matches. As shown in figure S14, the regions listed above are more hydrolysed in the samples from Pompeii, Herculaneum and Baia Scalandrone. The higher frequency of observed backbone cleavage seems to suggest a different state of preservation of bones embedded in volcanic deposits from those from agricultural soil.

#### Other diagenetically induced chemical modifications

**Figure 3:**
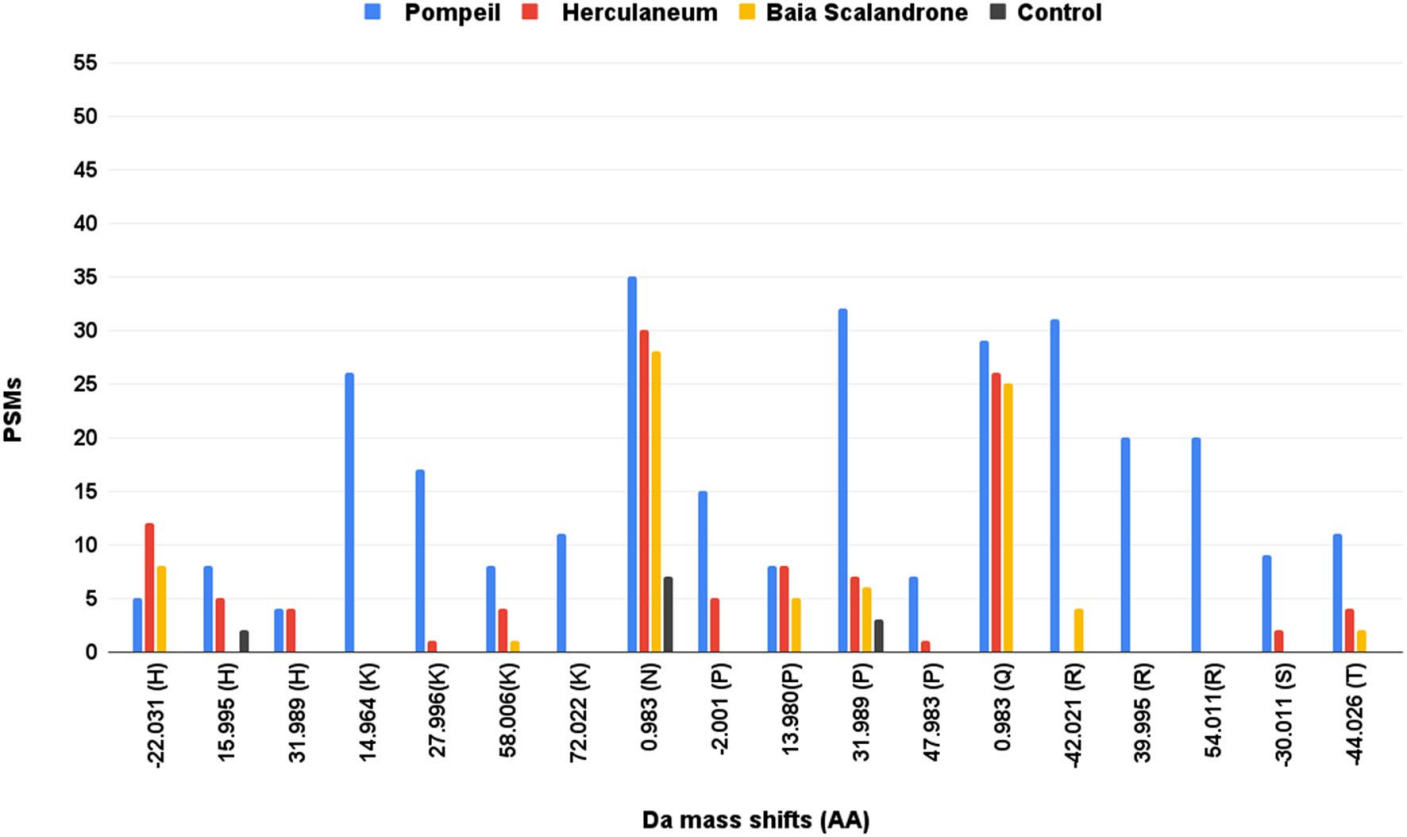
Peptide-spectrum matches (PSMs) of “dependent peptides” with mass shifts in the type I collagen chains in the sample groups of Pompeii, Herculaneum, Baia Scalandrone and control samples (H-142, H-162^34^). Mass shifts were selected after filtering with localization probabilities of ≥ 80% for modified peptides and occurrence of detection of DP Cluster Mass ≥ 5 times for each sample. Reported data only include mass shifts corresponding to known oxidative modifications with matching amino acid targets (Unimod, http://www.unimod.org/).

Data-depended peptide algorithm of MaxQuant^29,46^ was used for an blind search of chemical modifications (CMs) in the samples. The CMs were ranked by their occurrence within the dataset. The modifications were chosen after filtering with localization probabilities of ≥ 80% for modified peptides and occurrence of detection of DP Cluster Mass ≥ 5times for each sample (see Fig. 3). As expected, hydroxylation of prolines is fairly abundant, actually overwhelming most of the other modifications (and therefore omitted from the figure), as well as deamidation at asparagines and glutamines.

As a second step, the selected CMs were inserted as variable modifications in standard MaxQuant searches, by setting the modifications as variable in separate runs, for each group separately, as detailed in Table S2. To confirm peptide assignment, we manually inspected MS/MS spectra (and some examples are reported in the supplementary information, Figures S23–S28) thus allowing to confidently assess the site localization of the chemical modifications.

The frequency of modified residues in respect to the amino acid detection is reported in the tables S5 (A–E). Each position was considered only once in this calculation, even when the position was present in overlapping peptides. Furthermore, the frequency of chemical modifications at a specific primary structure position was semiquantitatively evaluated using the MaxQuant calculation of mod/base ratio as reported in^47^ (Figures S19–S22).

**Figure 4:**
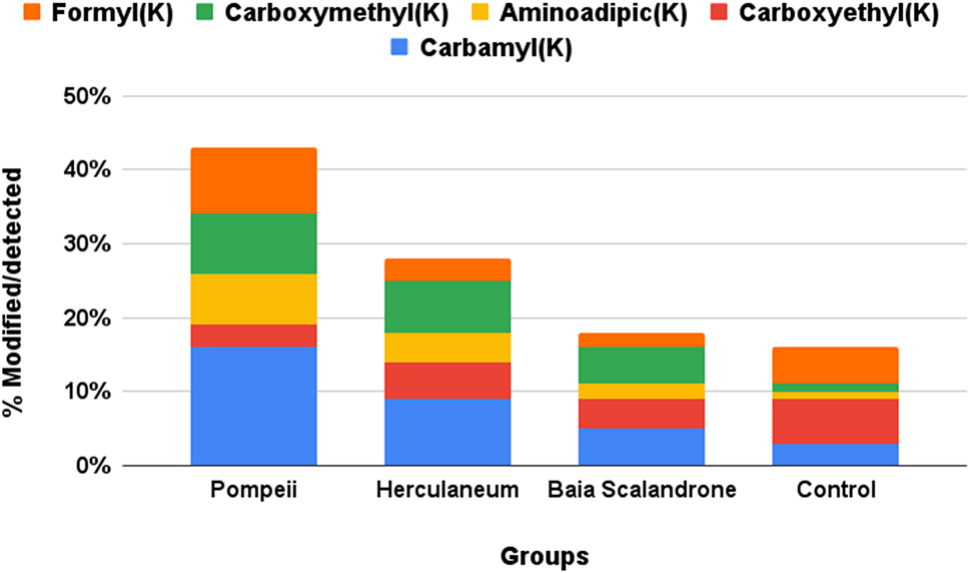
Extent of modified lysine residues, reported as percentage of modified over detected (modified plus unmodified) ones.

**Figure 5:**
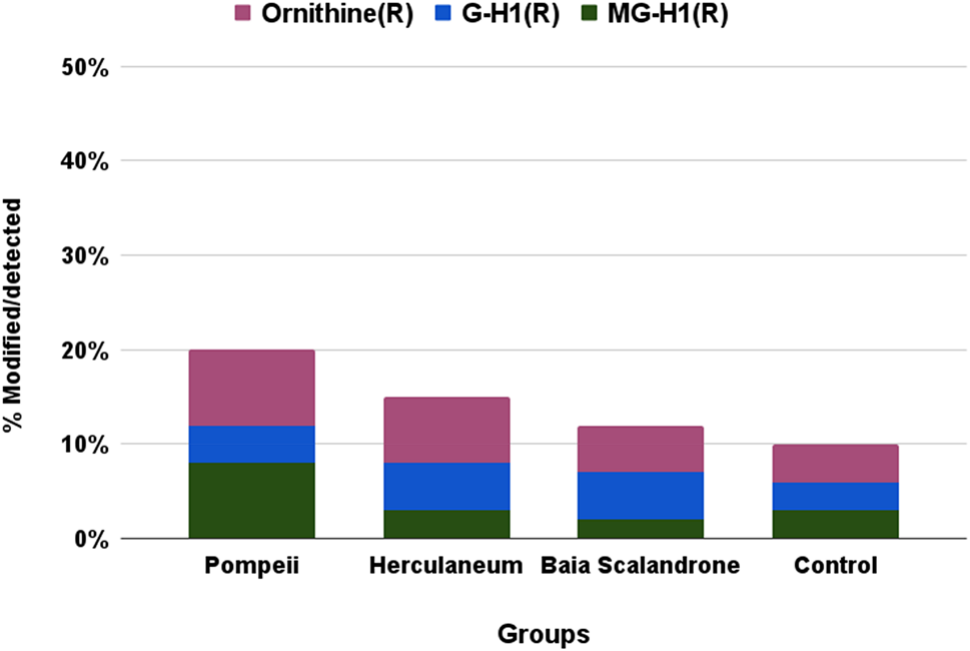
Extent of modified arginine residues, reported as percentage of modified over detected (modified plus unmodified) ones.

Interestingly, a high occurrence of mass shifts on lysine (K) and arginine (R) (Figs. 4 and 5) was observed, all, as expected, in correspondence of trypsin missed cleavages, that were interpreted as glycation products, with a high incidence in the samples group of Pompeii. Protein glycation involves the binding of reducing sugar carbonyl groups to protein amino groups, or the reaction of α-dicarbonyls such as glyoxal or methylglyoxal, that are continuously formed during oxidative degradation of sugars, with lysine and arginine residues, leading to a series of molecular reactions collectively called Maillard reaction that generate a variety of complex compounds called advanced glycation end products (AGEs)^48–51^. Among lysine-derived AGEs, N^ε^-(carboxymethyl)lysine (CML) and N^ε^-(carboxyethyl)lysine (CEL) are the most studied representatives and were significatively observed in the samples from the eruptive area (Fig. 4). Formylation at lysine side chains, oxidative deamination of lysine to aminoadipic acid, another marker of protein carbonyl oxidation^52^ that can be associated to decomposition after death^13^, and carbamylation, that has been reported as a hallmark of protein aging^53^, were all also observed in collagen from samples from Herculaneum and Pompeii. Among arginine-related AGEs we detected the hydroimidazolones MG-H1 and G-H1 formed by reaction of arginine side chain with the oxoaldehydes methylglyoxal and glyoxal^54^, respectively, and a substantial formation of ornithine (Figure S15 and fragmentation spectra at Figure S27)^55^, that was also recently identified in ancient dental enamel proteins^56^.

These modifications are less frequent in the control sample and, within samples from the volcanic areas, such modifications are significantly higher in bone collagen from Pompeii (Figs. 4 and 5).

**Figure 6:**
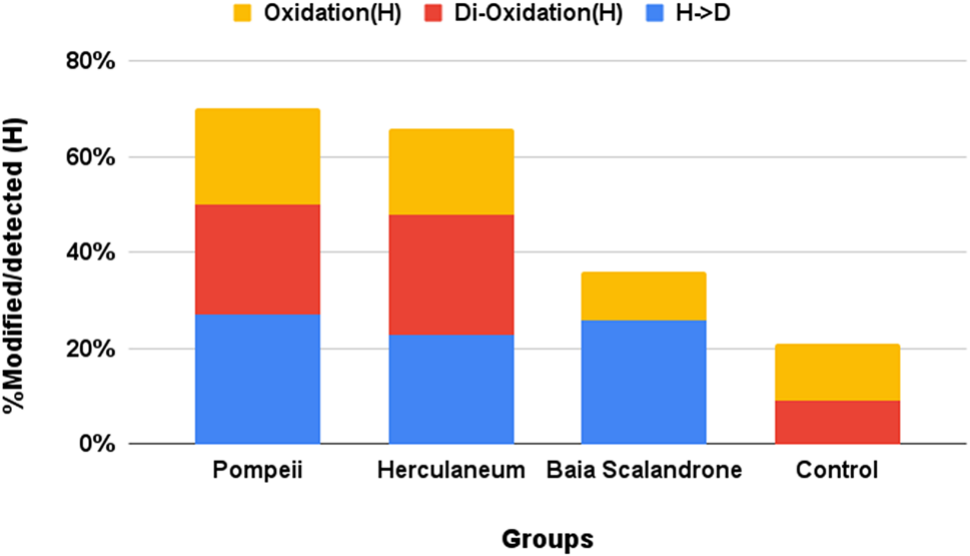
Extent of modified histidine residues, reported as percentage of modified over detected (modified plus unmodified) ones.

Histidine is one of the targets of oxidative modifications^39^, generating 2-oxohistidine and dioxohistidine that can evolve further to break down to aspartic acid. An extensive oxidation of histidine residues in collagen chains from the bones from the eruptive area was observed (Fig. 6). In fact, more than 65% of collagenous histidine residues in Pompeii and Herculaneum bone samples have been found modified (Table S5D). Interestingly, extensive evolution to aspartic acid has been observed in all the samples coming from the volcanic sites, comprising those from Baia Scalandrone, but not in the control samples, suggesting an influence of the alkalinity of volcanic soil in the final degradation product^57^.

**Figure 7:**
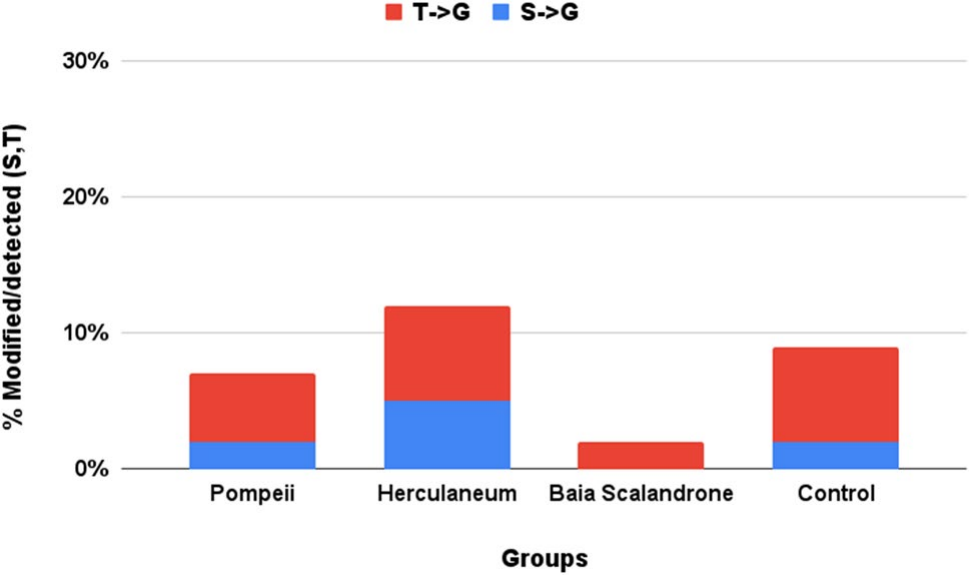
Extent of Cα-Cβ bond cleavage at serine and threonine reported as percentage of modified over detected (modified plus unmodified) residues.

Mass shifts that are consistent with the Cα-Cβ bond cleavage of the side chains of serine and threonine, which result in the formation of glycine (G) (− 30.011 Da and − 44.026 Da, respectively) were observed (Figs. 7, S16, S28). This modification resembles what recently reported on histidine residues^39^ and generally postulated as a result of radical transfer to backbone following oxidation reactions^58–60^, although it has never been reported so far for serine and threonine residues. However, this modification is not a prerogative of the bone samples here analysed, from volcanic sites, since it has been consistently observed also in the ancient bone control samples.

**Figure 8:**
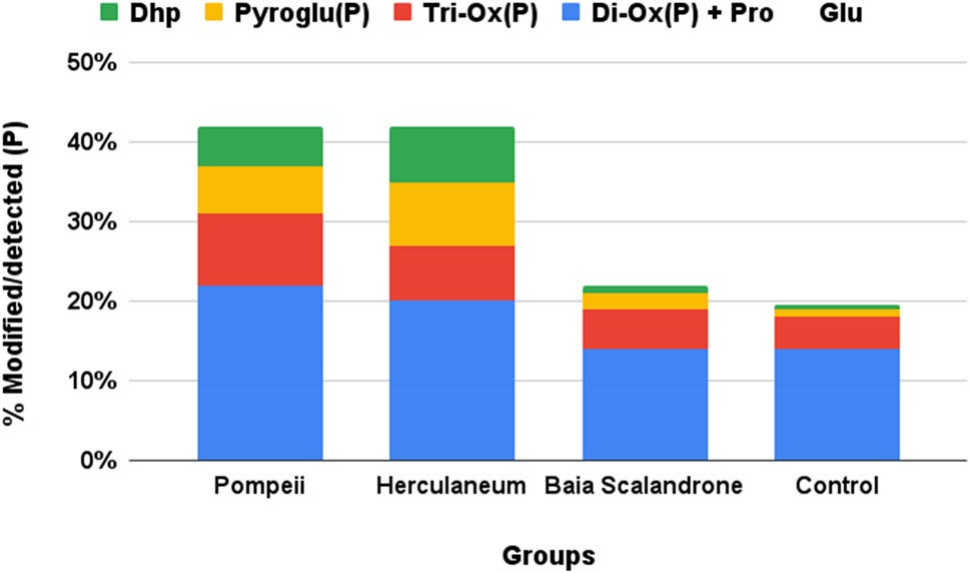
Extent of modified proline residues, reported as percentage of modified over detected (modified plus unmodified) ones.

Proline is a rather complex and often neglected target of chemical modification. The abundance of this residue in collagen, exceeding 20% of the total amino acids in human type I collagen, however, increases the rate of detection of modifications on this peculiar residue, although the abundant and variable incidence of hydroxylation makes detection of any other modification quite challenging (see Figure S17 for the occupancy of hydroxylation of proline along the sequences of COL1A1 and COL1A2). It has already been suggested that an increased level of hydroxylated prolines might result from a non-enzymatic oxidation^61^. The peculiar cyclic structure of proline results in an oxidative fate different from that of other aliphatic side chain^62^. Unfortunately, some oxidation products, such as glutamic semialdehyde are isobaric with hydroxylation^62,63^, impairing their unequivocal identification. Nevertheless, consistent formation of pyroglutamic acid from proline (ΔM + 13.980 Da) and di- and tri-oxidation products (ΔM + 31.989 Da and + 47.983 Da respectively), with di-oxidation that also matches formation of glutamic acid (Figure S18), are eventually suggestive of oxidative diagenetic modification (double hydroxylation is not reported as a physiological post-translational modification) (Figures S23–S26). Most interestingly, a mass shift of ΔM − 2.001 Da, consistent with the loss of 2 hydrogens, was repeatedly detected and only in the samples of Pompeii and Herculaneum (Fig. 8). We suggest (Figures S18 and S26) that this mass shift is attributed to 3,4 dehydro-proline, which is the only stable form of the five possible isomers of olefinic proline^64^, and could arise from dehydration of 4-hydroxyproline or 3-hydroxyproline. From now it will be called Dhp, with a mass shift of − 18.001 Da from hydroxyproline and ΔM − 2.001 Da from proline.

We also explored the occupancy of the non-enzymatic identified modifications along the sequence of COL1A1 and COL1A2. In general, the distribution of modifications is uneven, with residues with high modification occupancy and sites with low occupancy (Figures S19–S22). However, as far as the glycation products, that are the most striking peculiarity of Pompeii samples, rather interestingly, the G-H1 and MG-H1 modifications seem to be localized in some specific arginine positions, namely positions 564, 574, 1014, 1026 and 1034 of COL1A1, and positions 448, 474, 673 and 691 in COL1A2 (Figure S19). Conversely, in agreement with the observation of a higher average modification of lysines (according to Table S5A), glycation products on lysines seems more spread along the polypeptide chain in Pompeii samples (Figure S20). It is worth mentioning that almost all the lysine and arginine were actually covered.

More than 65% of the detected histidines in the samples from Pompeii and Herculaneum have been found modified (Table S5D). Figure S21 reports the occupancy of the identified modifications along the sequence of COL1A1 and COL1A2 in all the sample groups. Pompeii and Herculaneum samples behave quite similarly, and histidine 267 in COL1A1 seems to be a rather hot spot for oxidation.

Proline oxidation products are quite spread along the sequences (Figure S22) and follow the general trend of samples from Pompeii which are more modified than the Herculaneum ones. This is in agreement also with the observation that collectively 8% of prolines have been found to be modified (differently from hydroxylation) in samples from Pompeii and Herculaneum (Table S5C), which are in turn more modified than those from Baia Scalandrone and Control samples. Position 592 seems to be a hot spot in all the cases.

## Discussion

Bones can be considered time capsules, and individual history can be imprinted on their organic content^30^. Lack of intracellular proteins, extensive deamidation, backbone cleavage, oxidative chemical modifications, are all taphonomic marks of the diagenesis of organic matter. All these signs characterize the proteins extracted from the bones of human victims from Pompeii and Herculaneum, as a molecular imprint of the effects of the 79 AD eruption.

A striking feature is the almost complete absence of NCPs in the bones from Pompeii compared with those from Herculaneum, thus suggesting an incomplete consumption of the organic matter for the bones from Herculaneum. The latter hypothesis is in agreement with the evidence of preservation of organic tissue residues^22,23^ and organic compounds^24^.

Bodies in Pompeii experienced a different fate than those in Herculaneum, and the differences are also imprinted molecularly. The body flesh of Pompeii victims slowly disappeared, thus resulting in cavities between the skeleton and the volcanic ash^19^. In Herculaneum, instead, soft tissues underwent a rapid thermally-induced vanishing resulting in the complete body skeletonization and bones exceptionally well preserved^16^. The different proteomic content observed in Herculaneum bones in comparison with those from Pompeii is the result of the different environmental conditions due to exposure to different pyroclastic flows: the Pompeii victims were affected by the third and fourth pyroclastic surges, while at Herculaneum people were hit and buried by the first surge, which did not reach Pompeii^16^. Local environmental conditions during the eruption such as the peak of maximum temperature of the ash cloud and the time needed for the ash deposit to cool would have produced unique effects on the victims’ corpses and their bones.

The pathway of chemical reactions that break down the proteins within the bioapatite cage appears still fairly mysterious, with proteins normally degrading principally via a combination of two parallel as well as interplaying mechanisms: digestion by microbes and chemical modification/degradation^38^, with time, temperature and burial environment all contributing to influence the kinetics of protein decay. For instance, the presence of many copper minerals such as sulfates, oxides, carbonates, and phosphates in the volcanic soil may increase the solubility of hydroxyapatite thus leading possibly to partial bone loss^65^. Proteome complexity is generally considered a hallmark of bone degradation, inversely proportional to age, with most of the samples older than 20,000 years containing predominantly and almost exclusively collagen that benefits of the interaction with the bioapatite cage that protects them from degradation^35^. We can observe that in five of the seven Pompeii bones samples, collagen chains were the only proteins to be detected, and in the other two samples, beside collagen, only chondroadherin and biglycan were identified. Moreover, the lower NCPs content, the higher deamidation level and, in general, the higher extent of modification of collagen in the bones from Pompeii in respect to the bone samples from Herculaneum, demonstrate a more degraded state possibly as a result of the slower decomposition of soft tissue.

Despite the higher temperature that the bodies experienced at Herculaneum than at Pompeii, a good number of NCPS were identified in most of the bones. Only proteins stabilized by the binding to collagen or to the inorganic component of bones survived in Herculaneum, while all other proteins probably decayed rapidly due to the intense heat of the pyroclastic surge. The most common NCPs detected in the Herculaneum bones include Alpha-2-HS-glycoprotein, biglycan, chondroadherin, pigment epithelium derived factor (PEDF), lumican, and prothrombin, all proteins that are known to bind collagen or calcium ions. This evidence is in good agreement with proteins mostly identified in ancient bones^38,66^. Moreover, it was recently observed that fetuin-A (herein reported as Alpha-2-HS-glycoprotein), a serum glycoprotein, is relatively stable after death^36^. Here we observe that this protein, that prevents mineral precipitation during mineralization process by stabilizing supersaturated mineral solutions by forming soluble colloidal nanospheres^66^, is among the NCP survivors to the volcanic environmental conditions at Herculaneum.

Interestingly, in our samples, also Vitronectin survived quite well (it was identified in six of the seven samples from Herculaneum, as frequently as biglycan). This is an abundant multifunctional glycoprotein found in serum, extracellular matrix, and bone, involved in various physiological processes such as cell attachment, spreading, and migration, which interacts also with collagen type I^67^.

It is worth mentioning that none of the NCPs recently detected by immunological methods in calcined bone tissue^31^ has been identified herein by proteomic approach, while the set of proteins identified is in agreement with those recently identified by similar proteomic approach in rat model bones^68^.

NCPs were absent in samples from Pompeii. It might be hypothesized that, in the case of bones from Pompeii, where the body soft tissue survived much longer than in Herculaneum, proteins underwent a massive degradation process, possibly speeded up by the hot burial environment, thus resulting in skeletal remains with the fewest and most modified proteins.

Oxidative modifications in the 79 AD bone samples are extensive, very close to what expected to occur in a cooking process, which is still a debated question on a molecular basis^58^. Diagenetic increase of AGEs correlates with oxidative conditions^69,70^ and extensive glycation products were observed in the samples from Herculaneum and Pompei, always more pronounced in those from Pompeii, likely originating from reactions with the sugars originally present in the extracellular matrix. Histidine was herein confirmed as oxidative target among the amino acids^58^ and formation of radicals at C-α backbone can also eventually lead to backbone fragmentation^58^, thus suggesting an oxidative origin at the basis of the extensive backbone cleavage observed rather than hydrolysis in an environment where water evaporation is expected.

Several oxidative processes have been postulated to occur on prolines and hydroxyprolines upon heating, according to chemical pathway depicted by Hellwig^58^, who predicts hydroperoxides formation from addition of oxygen to radical at the aliphatic side-chain of prolines, as stable intermediates in protein oxidation.

The high incidence of prolines in collagen allowed to highlight the occurrence of oxidative modifications on this peculiar side chain, some of which possibly explained as modifications originating from hydroxyproline (such as that corresponding to a ΔM − 2.001 Da when considering proline as the unmodified amino acid).

**Figure 9:**
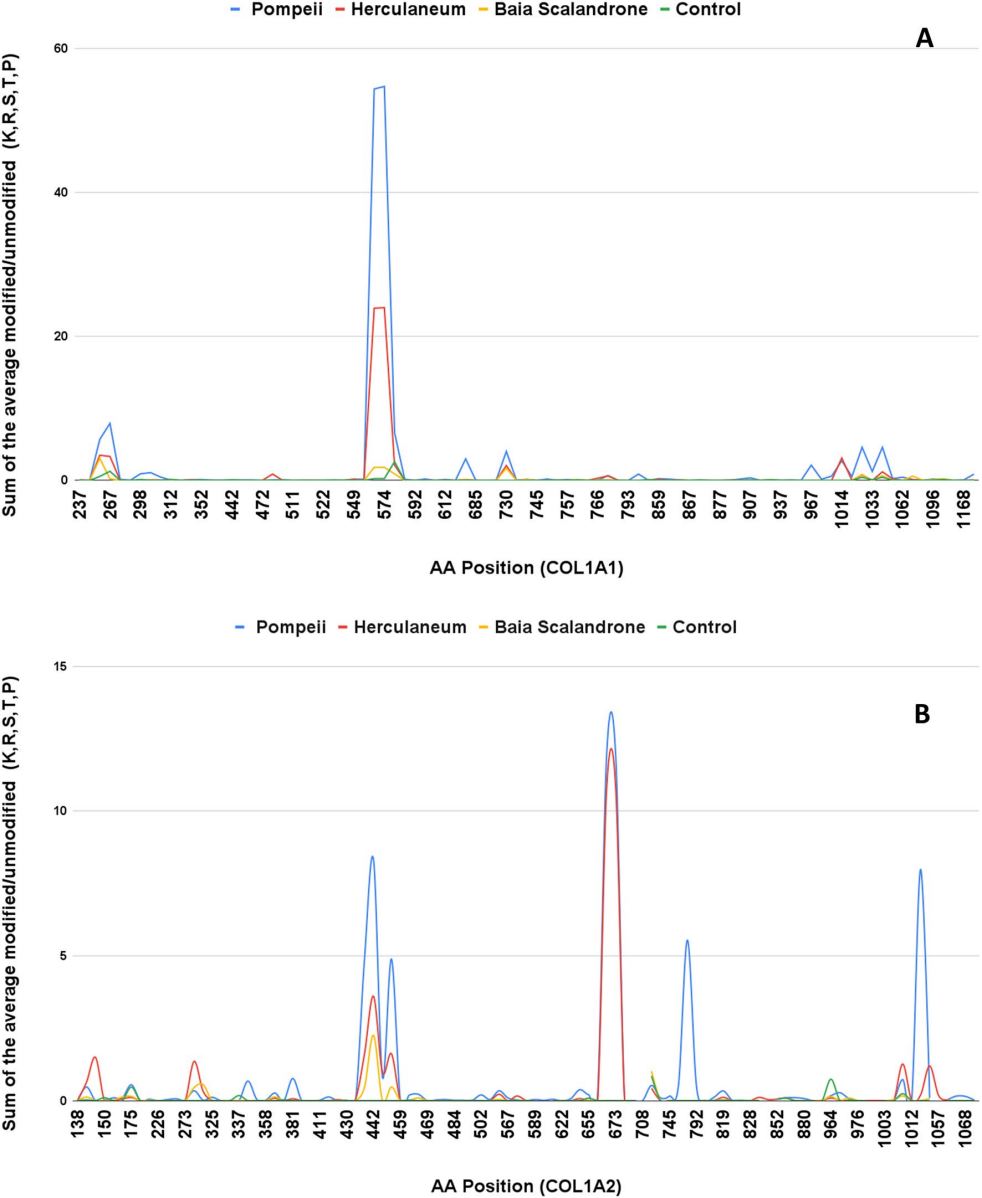
Comparative analysis of the global “damage signatures” in COL1A1 and COL1A2 from human bones of the different archaeological sites. The figure represents the sum of the average modified/unmodified values of K, R, S, T and P diagenetic modifications (except hydroxyproline and deamidation) at the specific primary structure positions of COL1A1 and COL1A2.

It is interesting to observe that modifications (although identified throughout the sequence), appear to be more pronounced in specific regions. In Fig. 9, diagenetic modifications are collectively showed along the collagen sequences, highlighting a different behaviour of the samples from Herculaneum and Pompeii in respect to those of Baia Scalandrone and control samples, that appear clearly less modified, with modifications spread along the sequence. This suggests a strong three-dimensional effect in directing chemical modifications events, an aspect that will deserve further future investigation.

These data do not claim to be conclusive of differences that we have highlighted in the diagenetic processes when comparing skeletal remains from Herculaneum and Pompeii, but rather demonstrate that molecular differences exist and can be seen as a perspective on the chemist’s approach to read through the processes that alter proteins in bone during burial. The history written in the molecules, a kind of “chemical life history tracer”. Despite the intra-samples’ variability observed, paleoproteomic analyses revealed that diagenetic processes generated by different environmental conditions are significantly reflected in the protein survival and modification. Why proteins survived better in the bones of the Herculaneum victims, whose body flesh rapidly disappeared, and why modifications were more evident in Pompeii bones are the main questions to be answered.

In this regard, it is important to highlight that bones from soils subjected to natural dry–wet hydrogeological cycles, as the case of Pompeii, show a low level of organic matter and high porosity^71^. The oxygen-rich environment during dry periods leads to a rapid degradation of the bone’s organic matter, and favours as well microbial activity^72^. Water level fluctuation induces leaching out and degradation of collagen due to increased solubility, leading to rapid destruction of the skeleton^73^. In contrast, permanently waterlogged sediments, as is the case with Herculaneum^74^, being anoxic, are able to inhibit microbial attack and related diagenetic processes^5,27^. The main characteristic of bone buried in a reducing and waterlogged stable environment is a high level of preservation of the organic and mineral matter, with a consequent low level of porosity/breakdown of the osseous structure^71^. Some microbes were also reported to have a preservative effect over time on bones^73,75^.

Soil chemistry also has an influence on bone preservation. Acidic soils are found to induce bone mineral loss, since an acidic environment is a main condition for bioapatite dissolution^76^. This diagenetic process can be increased by events as repeated wetting/drying soil cycles^77^, which in turn may accelerate the degradation of collagen^78^. In an acidic/corrosive soil, rapid bone mineral destruction and chemical alteration by microbial attack will occur. Vice versa, under alkaline to neutral conditions the organic and mineral bone components will be better preserved^76,78^. This seems to fit the case of the 79 AD eruption, where the chemical composition of volcanic deposits is primarily basic (alkaline-potassium sediment)^79^. A correlation between high fluorine (F) concentrations and alkaline soils has been also highlighted^80^. At Herculaneum, the waterlogged ash bed deposit is characterized by a fluoride-rich environment^25^. Fluorine enrichment of the bone transform bioapatite into a more thermodynamically stable phase^81^, thus giving the bone greater hardness, as also detected at Herculaneum^20,26^.

**Figure 10:**
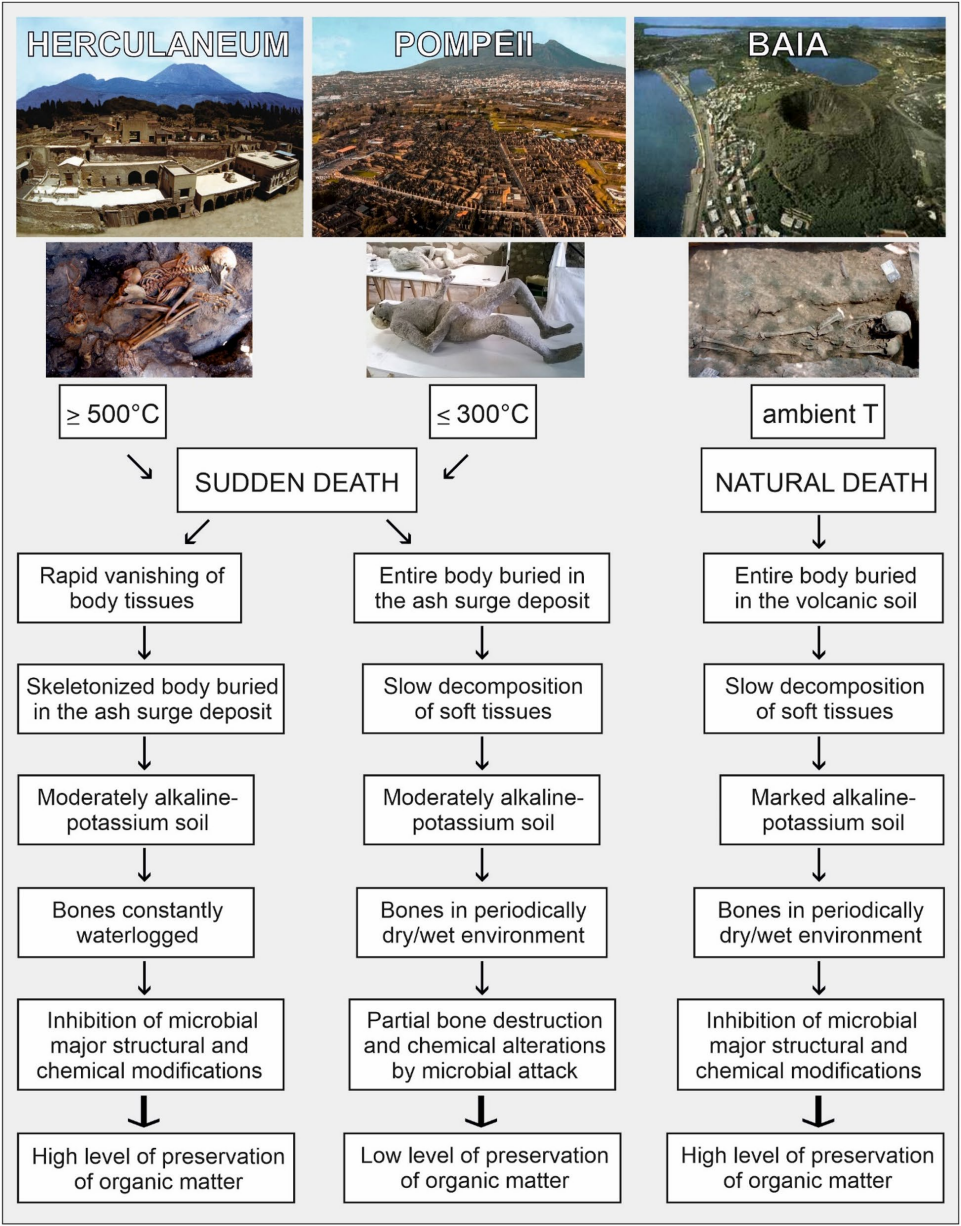
Sequence of biological and taphonomic events concerning the 79 AD human victims from Herculaneum and Pompeii, in comparison with the skeletons from the Baia Scalandrone graveyard.

Overall, the volcanic soils from the Campanian region are characterized by a high alkalinity (alkaline-potassium magmatism)^82^, with values even more marked for the Phlegraean Fields than those detectable for the Vesuvius area^83,84^. In addition, the alkalinity of groundwater from the Campanian volcanic areas, which originates from the leaching of alkaline-potassium pyroclastic deposits^85^, further supports the evidence of good preservation of organic and mineral matter in the bone^76,78^. Therefore, contrary to the assumptions of some authors^86^, the long-term good preservation of organic matter (i.e., collagen and other proteins) in the Herculaneum bones emerges as the result of the chemical-physical burial environment (ash bed deposit) rather than the effect of a not-so-high ash surge temperature. More in general, the extent of preservation of organic molecules in bones from Herculaneum and Pompeii, on the one hand, and Baia Scalandrone, on the other, regardless of whether or not they were exposed to heat, above all reflects the peculiarity of the interactions between the chemical-physical composition and the hydrogeological regime of the volcanic soils in which the bones were buried, characteristics that being different for each of the sites, produced different effects on the organic bone preservation (Fig. 10).

Finally, it is also important to stress that, in an anoxic environment, the extent of bone preservation depends on the mechanism by which the body is buried in the soil over time^78^. In this regard, the sequence of biological and taphonomic events that affected the victims’ corpses in Pompeii and Herculaneum, and the way the flesh of the body buried in the ash bed disappeared, appears to play a major role (Fig. 10). In Pompeii, the body tissues of the victims, killed by heat at 250–300 °C^17^ and then buried intact, underwent slow decay. The slow decomposition of soft tissues in a cycle of periodic wetting/drying of the soil appears to be the cause of poor preservation of organic matter. At Herculaneum, instead, after the rapid vanishing of soft tissue by ≥ 500 °C exposure^22^, the permanently waterlogged ash bed in which the skeleton was buried must have inhibited the microbial chemical modifications, allowing the long-term survival of organic matter. Such a type of environmental context seems to explain the reason of the highlighted good preservation of proteins, as well as the survival of collagen and DNA^24,87^.

Archaeological as well as forensic sciences will possibly benefit from the results herein obtained, since burned human skeletal remains are a common object of study for biological anthropologists, but they also represent a frequent type of evidence in the forensic scenario. Forensic proteomics is still in the early stages of development^88^, and the characterization of bone exposed to heat could be useful as an auxiliary strategy^31^. So far, a few proteomic analyses of bones in forensic context have been explored to estimate biological age (age-at-death)^30,36,89^ and post-mortem interval (PMI)^30,32,36^ of skeletal tissue^88^, or to distinguish individuals^90^, looking mainly at residual proteome complexity or to protein deamidation. Our results suggest that additional information can be found by expanding the set of modifications of proteins to look for, unveiling more details about taphonomic agents that may affect bone death processes, leading to find potential biomarkers for medicolegal investigations that can provide information about environmental parameters at the time of death.

## Methods

The skeletal elements of 15 individuals from the archaeological sites of Pompeii (7), Herculaneum (5) and Baia Scalandrone (3) were analysed. Table S1 describes each specimen and its related information and Figure S1 shows pictures of the samples and of the EDTA solubilised fraction. All necessary permits were obtained for the study of the human specimen from the Ethics Committee for Biomedical Activities, AOU Federico II, Naples, Italy, Protocol N. 101/17.

### Protein extraction, digestion and analyses

Bone samples were prepared as described in^34^ with slight modifications. Figure S2 represents the whole procedure, and a detailed description of the protocol is provided in the supplementary materials. Samples were processed as reported in^91^, and detailed in the supplementaries. Samples were separated on a 15 cm column (75 μm inner diameter) in-house laser pulled and packed with 1.9 μm C18 beads (Dr. Maisch, Germany) on an EASY-Nlc 1000 (Proxeon, Odense, Denmark) connected to a Q-Exactive HF (Thermo Scientific, Bremen, Germany).

### Data analysis

The resulting raw files (EvoG_sample name, in total 15 files) were searched and analysed using the MaxQuant (MQ) software^92^ against a UniProt database (759,512 sequences, 37,179,137 residues) with Homo sapiens as taxonomic restriction (20,199 sequences, 928,813 residues). Details of the different runs for standard proteins identification and searches for diagenetically induced modifications are provided in supplementary information and schematised in Table S2.

## Supplementary Information

Supplementary Information.

## Data Availability

LC–MS/MS data have been deposited to ProteomeXchange platform (http://proteomecentral.proteomexchange.org) with the dataset identifier PXD020462.
