# Author Correction: Molecular signatures written in bone proteins of 79 AD victims from Herculaneum and Pompeii

**DOI:** 10.1038/s41598-022-16362-5

**Published:** 2022-07-13

**Authors:** Georgia Ntasi, Ismael Rodriguez Palomo, Gennaro Marino, Fabrizio Dal Piaz, Enrico Cappellini, Leila Birolo, Pierpaolo Petrone

**Affiliations:** 1 Department of Chemical Sciences, University of Naples Federico II, Naples, Italy; 2 Evolutionary Genomics Section, Globe Institute, University of Copenhagen, Copenhagen, Denmark; 3 Department of Humanities, University Suor Orsola Benincasa, Naples, Italy; 4 Department of Medicine, Surgery and Dentistry, University of Salerno, Fisciano, Salerno, Italy; 5 Task Force Di Ateneo “Metodologie Analitiche Per La Salvaguardia Dei Beni Culturali”, University of Naples Federico II, Naples, Italy; 6 Department of Advanced Biomedical Sciences, Departmental Section of Legal Medicine, Anatomy and Histology, University of Naples Federico II, Naples, Italy

Correction to: *Scientific Reports*, 10.1038/s41598-022-12042-6, published on 27 May 2022

The original version of this Article contained errors in the author list, where Francesco Sirano was incorrectly listed as an author of the original Article, and has subsequently been removed.

The Author contributions section now reads:

“L.B., G.N., designed research; G.N., I.R.P. and L.B. performed research; P.P. collected samples and cured the bioanthropological and taphonomic aspects of the conceptualization; G.N., I.R.P. and L.B. analysed data; F.D.P., G.M., E.C. and L.B. supervised data analysis; G.N., P.P, G.M., E.C. and L.B. wrote the paper.”

Furthermore, the Acknowledgements section in the original version of this Article was omitted. It now reads:

“The authors would like to thank Meaghan Mackie at Globe Institute, University of Copenhagen, for her help with sample preparation and LC-MS/MS. Special thanks to the Archaeological Park of Herculaneum and the Archaeological Park of Pompeii (formerly called Pompeii Superintendence) for allowing the study of the bone samples used in this manuscript (Permit numbers “Soprintendenza di Pompei MIBACT-SSBA-PES, PROTO-ARCH 0008297 12/05/2016, CI. 16.04. 13/2” and “Parco Archeologico di Ercolano MIBAC-PA-ERCO, MBAC-PA-ERCO 0003371 13/09/2018, CI. 16.19. 40/1”).”

In addition, the Supplementary Information file that accompanies the original Article contained errors, where permission details were omitted. Under the heading ‘Description of skeletal elements’,

“The skeletal elements of 15 individuals from the archaeological sites of Pompeii (7), Herculaneum (5) and Baia Scalandrone (3) were analysed. Table S1 shows each specimen and its related information. All necessary permits were obtained for the study of the human specimen from the Ethics Committee for Biomedical Activities, AOU Federico II, Naples, Italy. Protocol N. 101/17.”

now reads:

“The skeletal elements of 15 individuals from the archaeological sites of Pompeii (7), Herculaneum (5) and Baia Scalandrone (3) were analysed. Table S1 shows each specimen and its related information. All necessary permits were obtained from the Archaeological Park of Herculaneum and the Archaeological Park of Pompeii (formerly called Pompeii Superintendence) (Permit numbers “Soprintendenza di Pompei MIBACT-SSBA-PES, PROTO-ARCH 0008297 12/05/2016, CI. 16.04. 13/2” and “Parco Archeologico di Ercolano MIBAC-PA-ERCO, MBAC-PA-ERCO 0003371 13/09/2018, CI. 16.19. 40/1”) and for the study of the human specimen from the Ethics Committee for Biomedical Activities, AOU Federico II, Naples, Italy. Protocol N. 101/17.”

Furthermore, in the section ‘Experimental procedure’ of the Supplementary Information file, under the subheading ‘Protein extractions and digestion’,

“Bone samples were prepared as described in (*34*) with slight modifications.”

now reads:

“Bone samples were prepared as based partly on a method described in (*34*) with modifications based on (*91*).”

And,

“The EDTA and Tris fraction were concentrated on an Amicon 3000 Da MWCO spin column, the flow through was discarded, and the retained sample was washed with 3ml of guanidinium hydrochloride (GuHCl) 2M extraction solution, 0.5M Tris (2-carboxyethyl) phosphine, 0.5M chloroacetamide, 100 mM Tris (pH 8.0) and water. Finally, the membrane was washed with 300 μl of GuHCl 2M extraction solution that were added to the protein pellet. The pH was adjusted to 8.0 and the mixture of proteins was heated at 80°C for 3 hours under agitation and cooled to room temperature. Without separating supernatants and the residual bone powder, samples were heated to 80 °C for 2 h, and cooled to room temperature LysC-Trypsin mix, 1/100 by amount of protein, was added to the samples for protein digestion. Mixed samples were incubated at 25 °C for 30 min then diluted to 2 M GuHCl with 25 mM Tris (pH 8.0), followed by incubation at 37 °C overnight under agitation. Digestion was terminated with 10% trifluoroacetic acid to a final concentration of 1%. After centrifugation at 14 000 g for 10 min, the tryptic peptides in the supernatant were immobilized on C18 stage tips as previously described (13).”

now reads:

“The EDTA and Tris fraction were concentrated on an Amicon 3000 Da MWCO spin column, the flow through was discarded, and the retained sample was washed with 3ml of guanidinium hydrochloride (GuHCl) 2M extraction solution containing 0.5M Tris (2-carboxyethyl) phosphine, 0.5M chloroacetamide, 100 mM Tris (pH 8.0) and water. Finally, the membrane was washed but not spun through with 300 μl of the above GuHCl 2M extraction solution which was subsequently added to the protein pellet. The pH was adjusted to 8.0 and the mixture of proteins was heated to 80 °C for 2 h and cooled to room temperature. Protein concentration was estimated by Bradford Assay. LysC, was added (0.2 µg) to the samples for initial protein digestion. Mixed samples were incubated at 37 °C for 90 min then diluted to 0.6 M GuHCl with 25 mM Tris (pH 8.0) in 10% acetonitrile (ACN), followed by incubation at 37 °C overnight under agitation with Trypsin (0.8 µg). Digestion was terminated with 10% trifluoroacetic acid (TFA) to a final concentration of 1%. After centrifugation at 14 000 g for 10 min, the tryptic peptides in the supernatant were immobilized on C18 stage tips as previously described (*13*).”

Additionally, in the same section ‘Experimental procedure’ of the Supplementary Information file, under the subheading ‘nLC-MSMS’,

“Samples were processed as reported in (*89*), and detailed below. Bone samples were eluted from the stage tips using 20 μL 40% ACN and then 10 μL 60% ACN into a 96 well MS plate.”

now reads:

“Samples were processed based on (91) and detailed below. Bone samples were eluted from the stage tips using 30 μL 40% ACN into a 96 well MS plate.”

And,

“Samples were then separated on a 15 cm column (75 μm inner diameter) in-house laser pulled and packed with 1.9 μm C18 beads (Dr. Maisch, Germany) on an EASY-Nlc 1000 (Proxeon, Odense, Denmark) connected to a Q-Exactive HF (Thermo Scientific, Bremen, Germany) on a 77 min gradient. Buffer A was milliQ water.”

now reads:

“5 μl of each sample was then separated on a 15 cm column (75 μm inner diameter) in-house laser pulled and packed with 1.9 μm C18 beads (Dr. Maisch, Germany) on an EASY-nLC 1200 (Proxeon, Odense, Denmark) connected to a Q-Exactive HF-X (Thermo Scientific, Bremen, Germany) on a 77 min gradient. Buffer A was milliQ water.”

Finally,

“The Q-Exactive HF was operated in data dependent top 10 mode. Spray voltage was 2 kV, S-lens 162 RF level at 50, and heated capillary to 275 °C. Full scan mass spectra were recorded at a resolution of 120,000 at m/z 200 over the m/z range 350–1400 with a target value of 3e6 and a maximum injection time of 25 ms. HCD-generated product ions were recorded with a maximum ion injection time set to 108 ms and a target value set to 2e5 and recorded at a resolution of 60,000. ”

now reads:

“The Q-Exactive HF-X was operated in data dependent top 10 mode. Spray voltage was 2 kV, S-lens RF level at 50, and heated capillary to 275°C. Full scan mass spectra were recorded at a resolution of 120,000 at m/z 200 over the m/z range 350–1400 with a target value of 3e6 and a maximum injection time of 25 ms. HCD-generated product ions were recorded with a maximum ion injection time set to 118 ms and a target value set to 2e5 and recorded at a resolution of 60,000 at a scan range of 200-2000 m/z.”

The original Article and its accompanying Supplementary Information file have been corrected.

## Supplementary Information

Supplementary Information.

