# Supplementary material for: Author Correction: Molecular signatures written in bone proteins of 79 AD victims from Herculaneum and Pompeii
Source: Sci Rep. 2022 Jul 13;12:11888. doi: 10.1038/s41598-022-16362-5 (PMC9279501; doi:10.1038/s41598-022-16362-5)
Supplement: Supplementary file 1 — Supplementary Information. [file 41598_2022_16362_MOESM1_ESM.pdf]

Supporting Information

Molecular signatures written in bone proteins of 79 AD victims from Herculaneum and Pompeii.

Georgia Ntasi, [1] Ismael Rodriguez Palomo, [2] Gennaro Marino, [2,3] Fabrizio Dal Piaz, [4] Francesco Sirano, [5] Enrico Cappellini, [2] Leila Birolo, *[,1,6] and Pier Paolo Petrone [6,7]

[1] Department of Chemical Sciences, University of Naples Federico II, Naples, Italy
[2] Evolutionary Genomics Section, Globe Institute, University of Copenhagen, Copenhagen, Denmark
[3] Department of Humanities, University Suor Orsola Benincasa, Naples, Italy
[4] Department of Medicine, Surgery and Dentistry, University of Salerno, Fisciano, Salerno, Italy.
[5] Archaeological Park of Herculaneum, Ercolano, Naples, Italy.
[6] Task Force di Ateneo "Metodologie Analitiche per la Salvaguardia dei Beni Culturali", University of Naples Federico II, Naples, Italy
[7] Department of Advanced Biomedical Sciences, Departmental Section of Legal Medicine, Anatomy and Histology, University of Naples Federico II, Naples, Italy.

Table of contents

Description of skeletal elements 4
Table S1. Specimens analysed. 4
Fig. S1. Photographic representation of each skeletal element, EDTA fraction, and state of bone preservation 5
Experimental procedures 7
Protein extraction and digestion 7
Fig. S2. Schematic representation of the workflow of protein extraction and digestion from each bone sample. 8
nLC-MSMS 8
Data analysis 9
Table S2. Details of the different MaxQuant (MQ) search runs and data analyses performed in this study 10
Evaluation of diagenetically induced chemical modifications. 13
Results 14
Protein Identification 14
Table S3. Proteins identified in the skeletal samples from Pompeii, Herculaneum and Baia Scalandrone archeological sites. 14
Fig. S3. Protein identification analysis. 20
Fig. S4. Venn diagram of proteins 21
Table S4. Unique and shared proteins between the different sample groups. 21
Fig. S5. STRING interaction pathway of bone proteins. 24
Deamidation (N, Q) 25
Fig. S6. Overall percentage of deamidation (N, Q) of non-collagenous bone proteins 25
Fig. S7. Percentage of deamidation (N, Q) of collagenous proteins 25
Fig. S8. Percentage of deamidation (N, Q) of non-collagenous proteins 26
Fig. S9. Visualization of deamidation level (N, Q) along the sequence of collagen 26
Oxidation (M) 27
Fig. S10. Percentage of oxidation (M) of collagenous proteins 27
Fig. S11. Percentage of oxidation (M) of non-collagenous proteins 28
Fig. S12. Visualization of oxidation level (M) along the sequence 29

Non-enzymatic backbone cleavage 30
Fig. S13. Example of the “pacman” effect 30
Fig. S14. Semi-quantitative evaluation of the backbone cleavage along the sequence 31
Other diagenetically induced chemical modifications 31
Table S5 (A-E). Occurrence of chemical modifications 31
Fig. S15. Conversion of Arginine to Ornithine 33
Fig. S16. Oxidation reaction at the side chain of Threonine (T) and Serine (S) 33
Fig. S17. Example of site-specific hydroxylation occupancy on Proline residues 34
Fig. S18. Possible Proline oxidation products. 35
Fig. S19. Site-specific modification on Arginine residues along the sequence 36
Fig. S20. Site-specific modification on Lysine residues along the sequence 37
Fig. S21. Site specific modification on Histidine residues along the sequence 38
Fig. S22. Primary structure modification occupancy on Proline residues along the sequence 39
Selected MS/MS spectra 40
Fig. S23 (a-b). MS/MS Spectra of COL1A1 GDAGPPGPAGPAGPPGPIGNVGAPGAK peptide 40
Fig. S24 (a-b). MS/MS Spectra of COL1A2 GPAGPSGPAGKDGR peptide 41
Fig. S25 (a-b). MS/MS Spectra of COL1A2 GIPGPVGAAGATGAR peptide. 42
Fig. S26 (a-b). MS/MS Spectra of COL1A1 GLTGSPGSPGPDGK peptide. 43
Fig. S27. MS/MS Spectra of COL1A2 SGDRGETGPAGPAAGPVGPVGAR peptide. 44
Fig. S28 (a-b). MS/MS Spectra of COL1A1 GSAGPPGATGFPGAAGR peptide 45
Fig. S29. View of the sea-front chambers on the beach of Herculaneum 46
Fig. S30. Fiberglass replica of the Herculaneum victims in chamber 11 47
Fig. S31. Plaster casts of human victims found in Pompeii 47

Description of skeletal elements

In the present study, the samples adopted for the bone proteomic profiling refers to human remains from the 79 AD eruption victims discovered in the pyroclastic ash surge deposits at Herculaneum and Pompeii [17,20]. The specimens from Herculaneum regard the victims' skeletons discovered in the early '80s in a series of sea-front chambers and on the ancient beach, in the suburban area of the town (Figure S. 29). The samples belong to some of the victims found in chambers 5 and 10 whose skeletons, once the appropriate surveys were carried out, were removed to be studied in the laboratory, and replaced by fiberglass replicas (Figure S. 30). In Pompeii, the victims were unearthed in both outdoor and indoor, and, where possible, a plaster cast was made (Fig. S. 31). Many casts are still in situ, others have been restored to be preserved at the site and, in some cases, even displayed in exhibitions (P. Petrone. Corpi umani come capsule del tempo, in: M. Osanna et al. (eds). *I calchi di Pompei da Giuseppe Fiorelli ad oggi*, Roma, 2021).

The skeletal elements of 15 individuals from the archaeological sites of Pompeii (7), Herculaneum (5) and Baia Scalandrone (3) were analysed. Table S1 shows each specimen and its related information. All necessary permits were obtained for the study of the human specimen from the Ethics Committee for Biomedical Activities, AOU Federico II, Naples, Italy. Protocol N. 101/17.

Table S1. Specimens analysed.

Skeletal code	Specimen	Sex/age	Skeletal element	Age context	Locality	Country	Latitude	Longitude
PC203	C. 46	F?/Adult	Rib	79 AD	Pompeii	Italy	40,74722222	14,49861111
PC204	C. 47	?/Adult	Femur	79 AD	Pompeii	Italy	40,74722222	14,49861111
PC205	C. 48	?/Infant	Skull	79 AD	Pompeii	Italy	40,74722222	14,49861111
PC206	C. 68	?/Adult	Tibia	79 AD	Pompeii	Italy	40,74722222	14,49861111
PC207	C. 69	M/Adult	Scapula	79 AD	Pompeii	Italy	40,74722222	14,49861111
PC193	C. 28	F/Juvenile	Rib	79 AD	Pompeii	Italy	40,74722222	14,49861111
PC195	C. 38B	M?/Adult	Femur	79 AD	Pompeii	Italy	40,74722222	14,49861111
ECC106	F5/3	M/Adult	Rib	79 AD	Herc	Italy	40,80611	14,3475
ECC108	F10/2	M/Juvenile	Rib	79 AD	Herc	Italy	40,80611	14,3475
ECC109	F10/8	M/Adult	Rib	79 AD	Herc	Italy	40,80611	14,3475
ECC117	F10/11a	F/Adult	Rib	79 AD	Herc	Italy	40,80611	14,3475
ECC118	F10/12	M/Adult	Rib	79 AD	Herc	Italy	40,80611	14,3475
BSC180	T. 3	M/Adult	Rib	II sec AD	Baia Scal	Italy	40.79647	14.07741
BSC182	T. 6	ND/Infant	Rib	II sec AD	Baia Scal	Italy	40.79647	14.07741
BSC183	T. 20	M?/Infant	Rib	II sec AD	Baia Scal	Italy	40.79647	14.07741

Legend: M = Male
ND = undetermined F = Female
? = dubitative Adult = 20 – x
Herc = Herculaneum Juvenile = 13-19
Scal = Scalandrone Infant = 0 - 6

Fig. S1. Photographic representation of each skeletal element, EDTA fraction, and state of bone preservation

Skeletal code	Skeletal element image	State of bone preservation	EDTA fraction image
ECC106	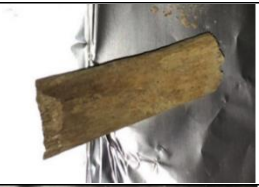	Compact outer bone, brittle inner bone	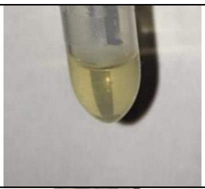
ECC108	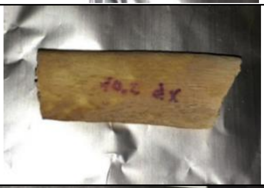	Compact outer bone, brittle inner bone	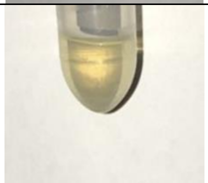
ECC109	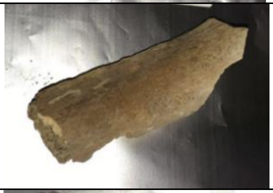	Compact outer bone, brittle inner bone	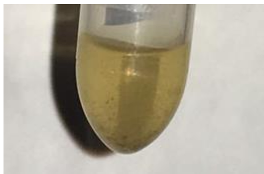
ECC117	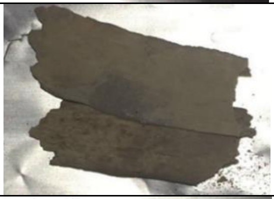	Brittle outer/inner bone	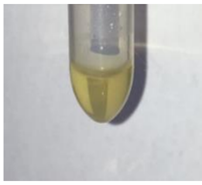
ECC118	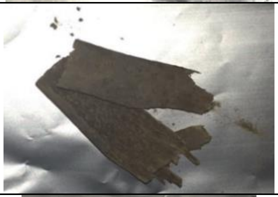	Brittle outer/inner bone	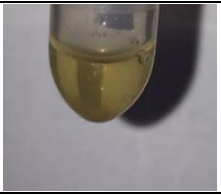
PC193	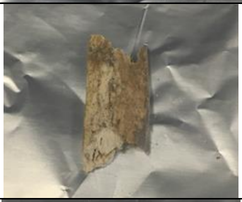	Brittle outer/inner bone	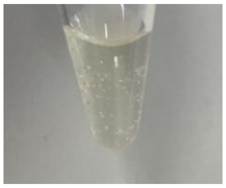
PC195	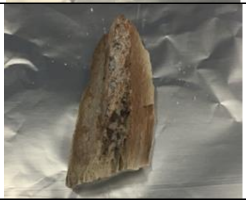	Compact outer bone, brittle inner bone	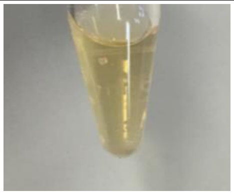

PC203	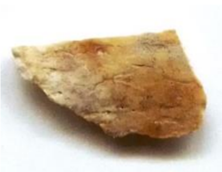	Compact outer bone, brittle inner bone	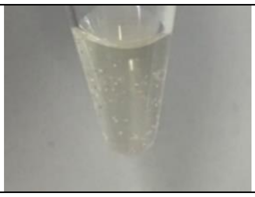
PC204	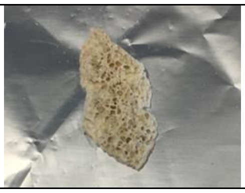	Brittle outer/inner bone	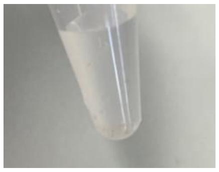
PC205	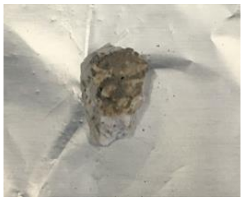	Brittle outer/inner bone	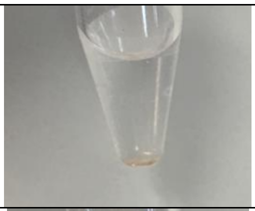
PC206	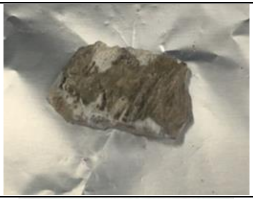	Brittle outer/inner bone	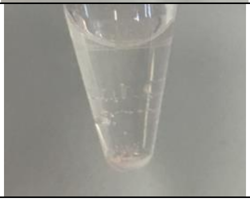
PC207	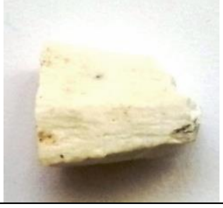	Brittle outer/inner bone	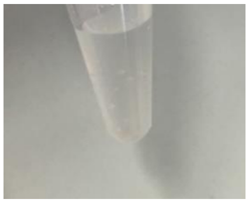
BSC180	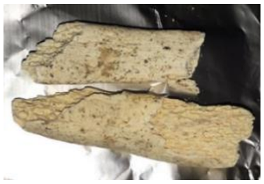	Brittle outer/inner bone	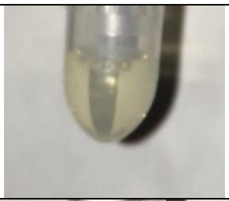
BSC182	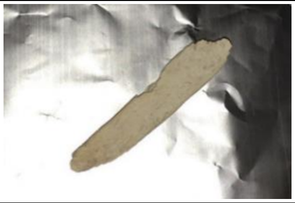	Compact outer bone, brittle inner bone	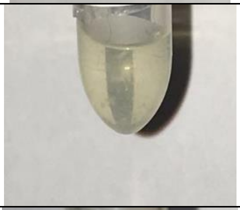
BSC183	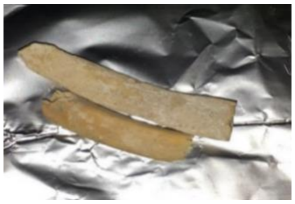	Compact outer bone	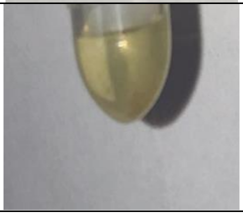

Experimental procedures

Protein extraction and digestion

Bone samples were prepared as described in (*34*) with slight modifications. Figure S2 represents the whole procedure.

Briefly, in order to prevent contamination, surfaces were removed from bone samples with sandpaper. Samples (30–50 mg) were wrapped in clean aluminum foil and fragmented into powder using a conventional hammer. The hammer was cleaned with bleach and ethanol, repeatedly. 300 µl of 0.5 M EDTA (pH 8.0) was added to the powdered bones, followed by incubation at 4 °C for 24–48 h with agitation. Every 24h fresh EDTA was added after the samples have been spun down and the supernatants were separated from the pellet (EDTA fraction). Subsequently, pellets were washed three times with 100 µl 0.1 M Tris (pH 8.0) and the supernatants (Tris fraction) were combined to the previous fraction (EDTA fraction). The EDTA and Tris fraction were concentrated on an Amicon 3000 Da MWCO spin column, the flow through was discarded, and the retained sample was washed with 3ml of guanidinium hydrochloride (GuHCl) 2M extraction solution, 0.5M Tris (2-carboxyethyl) phosphine, 0.5M chloroacetamide ,100 mM Tris (pH 8.0) and water. Finally, the membrane was washed with 300 µl of GuHCl 2M extraction solution that were added to the protein pellet. The pH was adjusted to 8.0 and the mixture of proteins was heated at 80°C for 3 hours under agitation and cooled to room temperature. Without separating supernatants and the residual bone powder, samples were heated to 80 °C for 2 h, and cooled to room temperature LysC-Trypsin mix, 1/100 by amount of protein, was added to the samples for protein digestion. Mixed samples were incubated at 25 °C for 30 min then diluted to 2 M GuHCl with 25 mM Tris (pH 8.0), followed by incubation at 37 °C overnight under agitation. Digestion was terminated with 10% trifluoroacetic acid to a final concentration of 1%. After centrifugation at 14 000 g for 10 min, the tryptic peptides in the supernatant were immobilized on C18 stage tips as previously described (*13*).

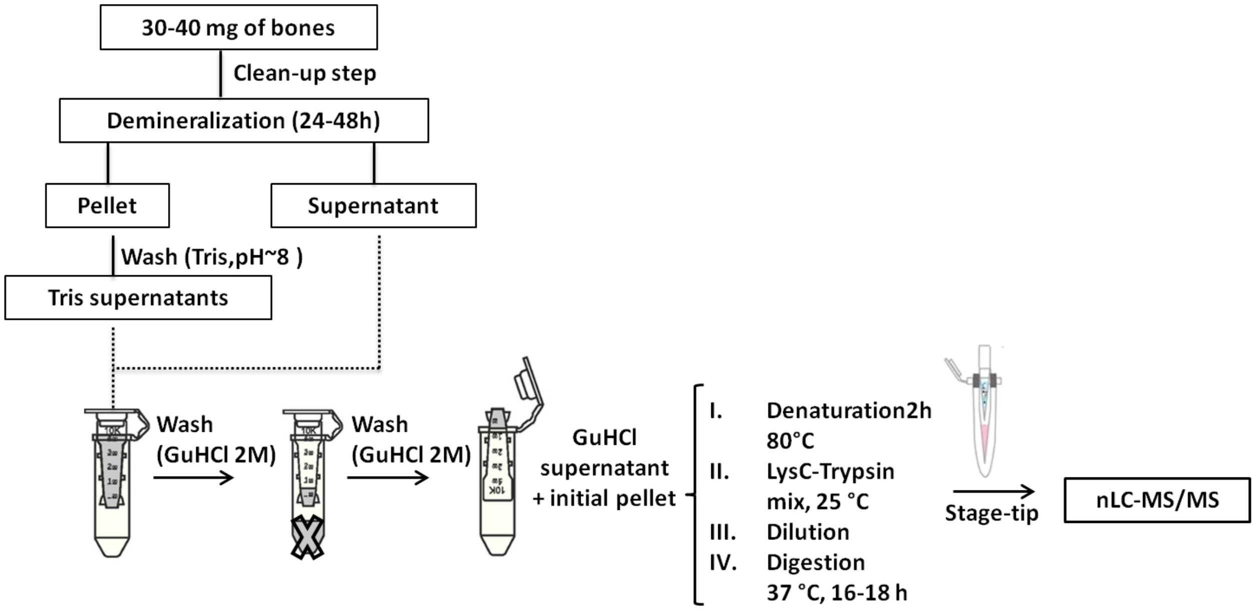

Fig. S2. Schematic representation of the workflow of protein extraction and digestion from each bone sample.

nLC-MSMS

Samples were processed as reported in (*89*), and detailed below.

Bone samples were eluted from the stage tips using 20 µL 40% ACN and then 10 µL 60% ACN into a 96 well MS plate. Samples were concentrated under vacuum at 40ºC up to approximately 3 µL, and then 5 µL of 0.1% TFA and 5% ACN were added.

Samples were then separated on a 15 cm column (75 µm inner diameter) in-house laser pulled and packed with 1.9 µm C18 beads (Dr. Maisch, Germany) on an EASY-Nlc 1000 (Proxeon, Odense, Denmark) connected to a Q-Exactive HF (Thermo Scientific, Bremen, Germany) on a 77 min gradient. Buffer A was milliQ water. The peptides were separated with increasing buffer B (80% ACN and 0.1% formic acid), going from 5% to 30% in 50 min, 30% to 45% in 10 min, 45% to 80% in 2 min, held at 80% for 5 min before dropping back down to 5% in 5 min and held for 5 min. Flow rate was 250 nL/min. The column temperature was maintained at 40°C using an integrated column oven. A wash-blank method using 0.1% TFA, 5% ACN was run in between each sample to hinder cross contamination.

The Q-Exactive HF was operated in data dependent top 10 mode. Spray voltage was 2 kV, S-lens RF level at 50, and heated capillary to 275°C. Full scan mass spectra were recorded at a resolution of 120,000 at m/z 200 over the m/z range 350–1400 with a target value of 3e6 and a

maximum injection time of 25 ms. HCD-generated product ions were recorded with a maximum ion injection time set to 108 ms and a target value set to 2e5 and recorded at a resolution of 60,000. Normalized collision energy was set at 28% and the isolation window was 1.2 m/z with the dynamic exclusion set to 20 s.

LC-MS/MS data have been deposited to ProteomeXchange platform (http://proteomecentral.proteomexchange.org) with the dataset identifier PXD020462.

Data analysis

The resulting raw files (EvoG_sample name, in total 15 files) were searched and analysed using the MaxQuant (MQ) software (*90*) against a UniProt database (759,512 sequences, 37,179,137 residues) with *Homo sapiens* as taxonomic restriction (20199 sequences, 928,813 residues). Initially standard searches (1-3, table S2) were performed, tolerances were those preset for Orbitrap, using a tryptic search with up to two missed cleavages. Minimum peptide length was set to 7. Carbamidomethylation was set as a fixed modification, while methionine oxidation, hydroxylation of proline, Gln-pyro Glu-pyro-Glu- were set as variable modifications, up to a maximum of 5 modifications per peptide. Protein identifications were supported by a false discovery rate (FDR) of 0.01 applied (same FDR for dependent peptides when applied) and manually filtered by at least 2 different non overlapping peptides above 70 ion score threshold. Contaminant proteins were assessed using the contamination.fasta provided by MQ which includes common laboratory contaminants (*90*) (see MaxQuant Downloads-contaminants.fasta,) can be found under http://www.coxdocs.org/doku.php?id=maxquant:start_downloads.htm, n.d. These protein hits were excluded from further analysis. MQ searches were also carried out on raw data from two archaeological Japanese bones samples that had been processed as herein samples from Pompeii and Herculaneum (*34*) (original work reference: samples H-142 and H-162, in the work by Sawafuji *et al*. (*34*)), but completely unrelated to the bones from Vesuvius area and belonging to more recent times (Hitotsubashi site AD 1657-1683), to compare and assess diagenetically induced chemical modifications in our skeletal samples.

In the search for non-enzymatic chemical modifications, we utilized the concept of “dependent peptides” for unbiased, comprehensive analysis of diagenetically induced chemical modifications in collagen proteins. The “dependent peptides” are modified peptides identified in an unrestricted database search against a library of previously identified nonmodified “base peptides”, a peptide search feature integrated in MaxQuant with statistical analysis for validation of modified peptides (S. Tyanova, T. Temu, J. Cox. The MaxQuant computational platform for mass spectrometry-based shotgun proteomics. *Nat. Protoc*. **11**, 2301–2319 (2016); M. M. Savitski, M. L. Nielsen, R. A. Zubarev, ModifiComb. A new proteomic tool for mapping substoichiometric post-translational modifications, finding novel types of modifications, and fingerprinting complex protein mixtures. *Mol. Cell. Proteomics*. **5**, 935–948 (2006)). This strategy was already demonstrated successful with painting samples (*39*) and *in vitro* metal ion induced oxidation (M.

Rykær, B. Svensson, M. J. Davies, P. Hägglund. Unrestricted Mass Spectrometric Data Analysis for Identification, Localization, and Quantification of Oxidative Protein Modifications. *J. Proteome Res.* **16**, 3978–3988 (2017)). In a separate run (run 4, table S2) all samples including the control samples (original work reference: samples H-142 and H-162, in the work by Sawafuji *et al*. (*34*)) were re-analysed and mass shifts were ranked by their occurrence within the dataset and grouped on the amino acid on which occurred. The modifications were firstly chosen after filtering with localization probabilities of ≥80% for modified peptides and occurrence of detection of DP Cluster Mass≥ 5times for each sample. Afterwards, alleged statistically relevant modifications for which a plausible chemical interpretation was given ("UNIMOD, protein modifications for mass spectrometry" http://www.unimod.org/modifications_list.php) were subsequently searched in separate runs, as group datasets (runs 5-24, table S2) as variable modifications in order to map selected PTMs on all identified as further confirmation. To reduce the search space, we restricted the database to the most relevant identified protein sequences, i.e. type I collagen chains, namely Collagen alpha-1(I) chain and Collagen alpha-2(I), which are, as expected, largely overrepresented, with more than 58% of the total assigned spectra on average. Moreover, type I collagen chains α1 and α2 are the two polypeptide chains identified in all samples. Backbone cleavage was searched against all groups in separate runs (25-28), inserting the standard parameters of the runs 1-3 and semitrypsin as enzyme in the MQ searches.

Table S2. Details of the different MaxQuant (MQ) search runs and data analyses performed in this study.

MQ search	Details on specific Parameters	Aim
1-3	Standard search in the UniProt human protein database (759,512 sequences, 37,179,137 residues) with *Homo sapiens* as taxonomic restriction (20199 sequences, 928,813 residues) to identify proteins in Pompeii, Herculaneum and Baia Scalandrone samples in separate runs (runs 1-3). Hydroxylation of Proline and Lysine, deamidation of Glutamine and Asparagine, oxidation of Methionine, Glutamine and Glutamic acid to Pyroglutamate were considered as variable modifications, carbamidomethylation of Cysteine was set as fixed modification; ion score cut off ≥ 70 for unmodified and modified peptides.<br>Dependent peptides search.	1 - Initial discovery of any common proteins found in human bones<br>2 - Untargeted discovery of AA substitutions and chemical modifications<br>3 - Evaluation of the PTMs frequency of detection
4	Addition of the two control archaeological bone samples (Control) in a separate run (run 4) in the	Comparison of PTMs among Pompeii,

	UniProt human protein database (759,512 sequences, 37,179,137 residues) with *Homo sapiens* as taxonomic restriction (20199 sequences, 928,813 residues). Hydroxylation of Proline and Lysine, deamidation of Glutamine and Asparagine, oxidation of Methionine, Glutamine and Glutamic acid to Pyroglutamate were considered as variable modifications, carbamidomethylation of Cysteine was set as fixed modification; ion score cut off ≥ 70 for unmodified and modified peptides.<br>Dependent peptides search.	Herculaneum and Baia Scalandrone with the control samples
5-8	Analysis of all archaeological bone samples, including controls with a database of the confidently identified *Homo sapiens* collagen proteins (163 sequences 176,326 residues) in separate runs (runs 6-10). Hydroxylation of Proline, deamidation of Glutamine and Asparagine, carbamylation, formylation, carboxyethylation, carboxymethylation of Lysine, and conversion of Lysine to aminoadipic acid were considered as variable modifications, carbamidomethylation of Cysteine was set as fixed modification; ion score cut off ≥ 70 for unmodified and modified peptides. Maximum 4 modifications per peptide.	Discovery of AGEs on lysine residues (K)
9-12	Analysis of all archaeological bone samples, including controls with a database of the confidently identified *Homo sapiens* collagen proteins (163 sequences 176,326 residues) in separate runs (runs 11-15). Hydroxylation of Proline, deamidation of Glutamine and Asparagine, formation of glyoxal, methylglyoxal at Arginine, and conversion of Arginine to Ornithine were considered as variable modifications, carbamidomethylation of Cysteine was set as fixed modification; ion score cut off ≥ 70 for unmodified and modified peptides. Maximum 4 modifications per peptide.	Discovery of AGEs on arginine residues (R)
13-16	Analysis of all archaeological bone samples, including controls with a database of the confidently identified *Homo sapiens* collagen proteins (163	Discovery of modifications on histidine residues (H)

	sequences 176,326 residues) in separate runs (runs 11-15). Hydroxylation of Proline, deamidation of Glutamine and Asparagine, mono-oxidation and bi-oxidation of Histidine, and conversion of Histidine to Aspartic acid were considered as variable modifications, carbamidomethylation of Cysteine was set as fixed modification; ion score cut off ≥ 70 for unmodified and modified peptides. Maximum 4 modifications per peptide.	
17-20	Analysis of all archaeological bone samples, including controls with a database of the confidently identified *Homo sapiens* collagen proteins (163 sequences 176,326 residues) in separate runs (runs16-20). Hydroxylation of Proline, deamidation of Glutamine and Asparagine, Proline oxidation to pyroglutamic acid, Proline di-oxidation, tri-oxidation and dehydration of Hydroxyproline (Dhp) were considered as variable modifications, carbamidomethylation was set as fixed modification; ion score cut off ≥ 70 for unmodified and modified peptides. Maximum 4 modifications per peptide.	Discovery of modifications on proline residues (P)
21-24	Analysis of all archaeological bone samples, including controls with a database of the confidently identified *Homo sapiens* collagen proteins (163 sequences 176,326 residues) in separate runs (runs11-15). Hydroxylation of Proline, deamidation of Glutamine and Asparagine, oxidation of Methionine and conversion of Serine and Threonine to Glycine were considered as variable modifications, carbamidomethylation was set as fixed modification; ion score cut off ≥ 70 for unmodified and modified peptides. Maximum 4 modifications per peptide.	Discovery of modifications on serine and threonine residues (S, T)
25-27	Backbone cleavage (Semitryptic analysis) search in the UniProt human protein database (759,512 sequences, 37,179,137 residues) with *Homo sapiens* as taxonomic restriction (20199 sequences, 928,813 residues) to identify proteins in Pompeii,	Discovery of non-enzymatic backbone cleavages

	Herculaneum and Baia Scalandrone samples in separate runs (runs 25-27). Hydroxylation of Proline and Lysine, deamidation of Glutamine and Asparagine, oxidation of Methionine, Glutamine and Glutamic acid to pyroglutamate were considered as variable modifications, carbamidomethylation was set as fixed modification; ion score cut off ≥ 70 for unmodified and modified peptides.	
28	Backbone cleavage (semitryptic analysis) search of the two control archaeological bone samples (Control) in a separate run (run 28) in the UniProt human protein database (759,512 sequences, 37,179,137 residues) with *Homo sapiens* as taxonomic restriction (20199 sequences, 928,813 residues). Hydroxylation of Proline and Lysine, deamidation of Glutamine and Asparagine, oxidation of Methionine, Glutamine and glutamic acid to pyroglutamate were considered as variable modifications, carbamidomethylation was set as fixed modification; ion score cut off ≥ 70 for unmodified and modified peptides.	Discovery of non-enzymatic backbone cleavages

Evaluation of diagenetically induced chemical modifications.

Deamidation of Asparagine (N) and Glutamine (Q), oxidation of Methionine (M), and hydroxylation of Proline (P) and Lysine (K) were evaluated as variable modifications in standard MaxQuant searches. Additionally, MaxQuant's "evidence.txt" file was used to calculate separate deamidation rates for N and Q for individual samples with a code that is freely available on GitHub (https://github.com/dblyon/deamidation). Also using both "evidence.txt" and "pepetides.txt", positional evaluation of the relative deamidation (N, Q) and oxidation (M) was performed using code available on https://github.com/ismaRP/MSMSdeamidation. For each N or Q position, the values are calculated dividing the sum of intensities of the peptides containing the modification by the total intensity of the peptides containing that position.

Backbone cleavage was assessed in all samples (runs 25-28, table S2) searching for semitryptic peptides allowing for one non-specific end by setting semitrypsin as enzyme in the MQ searches. Search for semi-tryptic peptides was carried out on collagen type I chains only, since they are the only polypeptide chains shared among all the samples. In terms of accuracy, only the peptides

with the MSMS count more than 1 and one unspecific cleavage site were considered. The frequency of semitryptic peptides was evaluated by calculating the percentage of semitryptic peptides over the total number of identified peptides for each chain, counting peptide to spectrum matches (PSMs) from the peptides.txt files of MQ (*13*)(M. Rykær, B. Svensson, M. J. Davies, P. Hägglund. Unrestricted Mass Spectrometric Data Analysis for Identification, Localization, and Quantification of Oxidative Protein Modifications. *J. Proteome Res.* **16**, 3978–3988 (2017)). Subsequently, manual alignment of all the semitryptic peptides in the five different groups of collagen type I sequences was performed in search of collagen regions that are most frequently hydrolysed. The cleavage frequency was then re-evaluated considering the regions rather than the single peptide bonds (window of ±2 AA) by calculating the number of PSMs with semitryptic cleavages identified in a region divided by the total PSMs in the same region, including both tryptic and semitryptic matches.

Non-enzymatic chemical modifications were initially evaluated by manual inspection of all MS/MS spectra, thus also allowing to confidently assess the site localization of the selected chemical modifications. The relative abundance of a mass shift associated with a type of amino acid [i.e. +31.989(P)] was calculated for each group, as an average of the modified amino acids normalized for the sum of all the corresponding detected amino acids in the primary structures of COL1A1 and COL1A2. The number of the modified positions was extracted from the modificationSites.txt files of MQ excluding potential false positives without an unmodified counterpart and excluding overlap (each primary structure position was considered once) although the number of the unmodified positions from the evidence.txt files. On the other hand, the modification occupancy at a specific primary structure position for each modification was calculated from the mod/base values extracted from the modification(X)site.txt files of MaxQuant (*47*). Ratios of the modified *vs* non-modified peptides were determined for each sample and then averaged for each group. These average ratios were then used to determine the significance of each modification in the archaeological samples in comparison to the average ratios of the control samples. Finally, in order to have a total vision of collagen "damage" among the different groups, the sum of the average ratios modified/unmodified (K, R, P, S, T) was calculated along the sequence of COL1A1 and COL1A2.

Results

Protein Identification

Table S3. Proteins identified in the skeletal samples from Pompeii, Herculaneum and Baia Scalandrone archaeological sites. Proteins were identified by searching by MAxQuant the UniProt protein database (759,512 sequences, 37,179,137 residues) with *Homo sapiens* as taxonomic restriction (20199 sequences, 928,813 residues. Carbamidomethylation of Cysteine was set as fixed modification; hydroxylation of Proline and Lysine, deamidation of Glutamine and Asparagine, oxidation of Methionine, Glutamine and Glutamic acid to pyroglutamate were

considered as variable modifications; ion score cut off ≥ 70 for unmodified and modified peptides. Proteins were considered as identified only when 2 or more peptides have been detected. LC-MS/MS data have been deposited to ProteomeXchange platform (http://proteomecentral.proteomexchange.org) with the dataset identifier PXD020462.

Skeletal sample code	Protein	UniProt entry	Gene name	Razor + Unique peptides	Unique peptides	Sequence coverage (%)	Sequence length
**PC203**	Collagen alpha-1(I) chain	P02452	COL1A1	58	57	47.6	1464
	Collagen alpha-2(I) chain	P08123	COL1A2	57	57	52.2	1364
**PC204**	Collagen alpha-1(I) chain	P02452	COL1A1	80	78	53.7	1464
	Collagen alpha-2(I) chain	P08123	COL1A2	65	65	57.5	1364
**PC205**	Collagen alpha-1(I) chain	P02452	COL1A1	61	59	43.2	1464
	Collagen alpha-2(I) chain	P08123	COL1A2	58	58	53.6	1364
**PC206**	Collagen alpha-2(I) chain	P08123	COL1A2	37	37	41.3	1364
	Collagen alpha-1(I) chain	P02452	COL1A1	39	38	37.4	1464
	Collagen alpha-1(II) chain	P02458	COL2A1	2	2	3.5	1418
**PC207**	Collagen alpha-2(I) chain	P08123	COL1A2	62	62	53.4	1364
	Chondroadherin	O15335	CHAD	3	3	11.4	359
	Collagen alpha-1(I) chain	P02452	COL1A1	89	88	55.5	1464
	Biglycan	P21810	PGS1	3	3	10.9	368
**PC193**	Collagen alpha-2(I) chain	P08123	COL1A2	93	93	65.5	1364
	Collagen alpha-1(I) chain	P02452	COL1A1	114	112	59.9	1464
	Chondroadherin	O15335	CHAD	5	5	17.8	359
	Collagen alpha-2(V) chain	P05997	COL5A2	6	6	7.4	1499
	Collagen alpha-1(II) chain	P02458	COL2A1	3	3	4.2	1418
**PC195**	Collagen alpha-2(I) chain	P08123	COL1A2	30	30	36.7	1364
	Collagen alpha-1(I) chain	P02452	COL1A1	31	30	30.5	1464
**ECC106**	Collagen alpha-	P08123	COL1A2	16	16	19.4	1464

	1(I) chain						
	Collagen alpha-2(I) chain	P02452	COL1A1	11	11	17.1	1364
	Hemoglobin subunit beta	P68871	HBB	2	1	25.6	90
	Pigment epithelium-derived factor	P36995	SERPINF1	2	2	5.3	418
	Prothrombin	P00734	F2	2	2	1.9	583
**ECC108**	Collagen alpha-2(I) chain	P08123	COL1A2	82	82	58.7	1364
	Collagen alpha-1(I) chain	P02452	COL1A1	106	103	58	1464
	Pigment epithelium-derived factor	P36995	SERPINF1	19	19	53.1	418
	Chondroadherin	O15335	CHAD	16	16	44.6	359
	Biglycan	P21810	BGN	15	15	41.6	368
	Alpha-2-HS-glycoprotein	P02765	AHSG	5	5	25.8	368
	Prothrombin	P00734	F2	11	11	20.8	583
	Vitronectin	P04004	VTNC	7	7	16.3	478
	Lumican	P51884	LUM	4	4	15.1	338
	Antithrombin-III	P01008	SERPINC1	3	3	14.7	464
	Decorin	P07585	DCN	5	5	14.2	359
	Periostin	Q15063	POSTN	4	4	11.4	808
	Osteomodulin	Q99983	OMD	3	3	9.7	421
	Protein Z-dependent protease inhibitor	Q9UK55	SERPINA10	2	2	9.5	484
	Kininogen-1	P01042	KNG1	3	3	7.3	427
	Collagen alpha-2(V) chain	P05997	COL5A2	3	3	6.5	1112
	Olfactomedin-like protein 3	Q9NRN5	OLFL3	2	2	6.2	406
	Collagen alpha-1(II) chain	P02458	COL2A1	2	2	4.5	1418
	Coagulation factor IX	P00740	F9	2	2	3.5	423
	Collagen alpha-1(XXII) chain	Q8NFW1	COL22A1	3	3	3	1319
	Collagen alpha-1(XII) chain	Q99715	COL12A1	6	6	2.5	3062

	Collagen alpha-1(III) chain	P02461	COL3A1	2	2	2.3	1466
	NHS-like protein 2	Q5HYW2	NHSL2	2	2	0.8	950
	Basement membrane-specific heparan sulfate proteoglycan core protein	P98160	HSPG2	2	2	0.8	4391
**ECC109**	Collagen alpha-2(I) chain	P08123	COL1A2	77	77	61.8	1364
	Collagen alpha-1(I) chain	P02452	COL1A1	98	96	57.2	1464
	Chondroadherin	O15335	CHAD	5	5	22.3	359
	Biglycan	P21810	BGN	4	4	13	368
	Collagen alpha-2(V) chain	P05997	COL5A2	3	3	5.8	1112
	Vitronectin	P04004	VTNC	2	2	4.8	478
**ECC117**	Collagen alpha-1(I) chain	P02452	COL1A1	94	92	58	1464
	Collagen alpha-2(I) chain	P08123	COL1A2	63	63	51.5	1364
	Chondroadherin	O15335	CHAD	15	15	42.6	359
	Pigment epithelium-derived factor	P36995	SERPINF1	10	10	33.5	418
	Biglycan	P21810	PGS1	13	13	28.5	368
	Alpha-2-HS-glycoprotein	P02765	AHSG	3	3	21.2	368
	Osteocalcin	P02818	BGLAP	2	2	20	100
	Protein Z-dependent protease inhibitor	Q9UK55	SERPINA10	6	6	16.9	484
	Prothrombin	P00734	F2	9	9	16.3	583
	Vitronectin	P04004	VTNC	7	7	16.3	478
	Lumican	P51884	LUM	4	4	15.1	338
	Antithrombin-III	P01008	SERPINC1	3	3	14.7	464
	Osteomodulin	Q99983	OMD	3	3	8.1	421
	Olfactomedin-like protein 3	Q9NRN5	OLFL3	2	2	6.2	406
	Kininogen-1	P01042	KNG1	2	2	4.9	427
	Coagulation factor IX	P00740	F9	3	3	4.7	423

	Collagen alpha-1(XXII) chain	Q8NFW1	COL22A1	2	2	2	1319
**ECC118**	Collagen alpha-2(I) chain	P08123	COL1A2	78	78	61.4	1364
	Collagen alpha-1(I) chain	P02452	COL1A1	98	97	56	1464
	Chondroadherin	O15335	CHAD	14	14	41.5	359
	Pigment epithelium-derived factor	P36995	SERPINF1	9	9	29.2	418
	Biglycan	P21810	PGS1	9	9	25.5	368
	Alpha-2-HS-glycoprotein	P02765	AHSG	4	4	23.9	368
	Lumican	P51884	LUM	4	4	15.1	338
	Antithrombin-III	P01008	SERPINC1	3	3	14.7	464
	Vitronectin	P04004	VTNC	6	6	12.6	478
	Decorin	P07585	DCN	4	4	10.9	359
	Prothrombin	P00734	F2	4	4	7.9	583
	Collagen alpha-2(V) chain	P05997	COL5A2	4	4	7.7	1112
	Osteomodulin	Q99983	OMD	2	2	7.4	421
**BSC180**	Collagen alpha-2(I) chain	P08123	COL1A2	47	47	51.5	1364
	Collagen alpha-1(I) chain	P02452	COL1A1	56	56	45.4	1464
**BSC182**	Collagen alpha-2(I) chain	P08123	COL1A2	67	67	56.7	1364
	Collagen alpha-1(I) chain	P02452	COL1A1	88	87	52.7	1464
	Vitronectin	P04004	VTNC	2	2	3.8	478
**BSC183**	Collagen alpha-1(I) chain	P02452	COL1A1	77	76	55.7	1464
	Collagen alpha-2(I) chain	P08123	COL1A2	67	67	53.1	1364
	Chondroadherin	O15335	CHAD	17	17	48.2	359
	Biglycan	P21810	PGS1	14	14	42.7	368
	Alpha-2-HS-glycoprotein	P02765	AHSG	6	6	28.8	368
	Pigment epithelium-derived factor	P36995	SERPINF1	10	10	26.6	418
	Protein Z-dependent protease inhibitor	Q9UK55	SERPINA10	6	6	19.6	484

	Vitronectin	P04004	VTNC	12	12	19.5	478
	Coagulation factor VII	P08709	F7	4	4	16.8	382
	Antithrombin-III	P01008	SERPINC1	4	4	16.8	464
	Prothrombin	P00734	F2	7	7	16.1	583
	Lumican	P51884	LUM	4	4	15.1	338
	Decorin	P07585	DCN	4	4	13.6	359
	Collagen alpha-1(II) chain	P02458	COL2A1	11	11	12.6	1418
	Kininogen-1	P01044	PNG1	3	3	10.5	427
	Collagen alpha-1(IX) chain	P20849	COL9A1	2	2	9.5	328
	Osteomodulin	Q99983	OMD	2	2	7.4	421
	Collagen alpha-2(XI) chain	P13942	COL11A2	9	9	6.7	1623
	Fibronectin	P02751	FN1	8	8	6.6	2031
	Cartilage oligomeric matrix protein	P49747	COMP	4	4	6.1	724
	Matrilin-3	O15232	MATN3	3	3	5.4	444
	Coagulation factor IX	P00740	F9	3	3	4.7	423
	Collagen alpha-1(X) chain	Q03692	COL10A1	3	3	3.7	680
	Collagen alpha-1(XI) chain	P12107	COL11A1	3	3	2.8	1690
	Collagen alpha-1(XII) chain	Q99715	COL12A1	4	4	2.1	3062
	Collagen alpha-1(XXII) chain	Q8NFW 1	COL22A1	2	2	2	1319
	Basement membrane-specific heparan sulfate proteoglycan core protein	P98160	HSPG2	6	6	1.9	4391

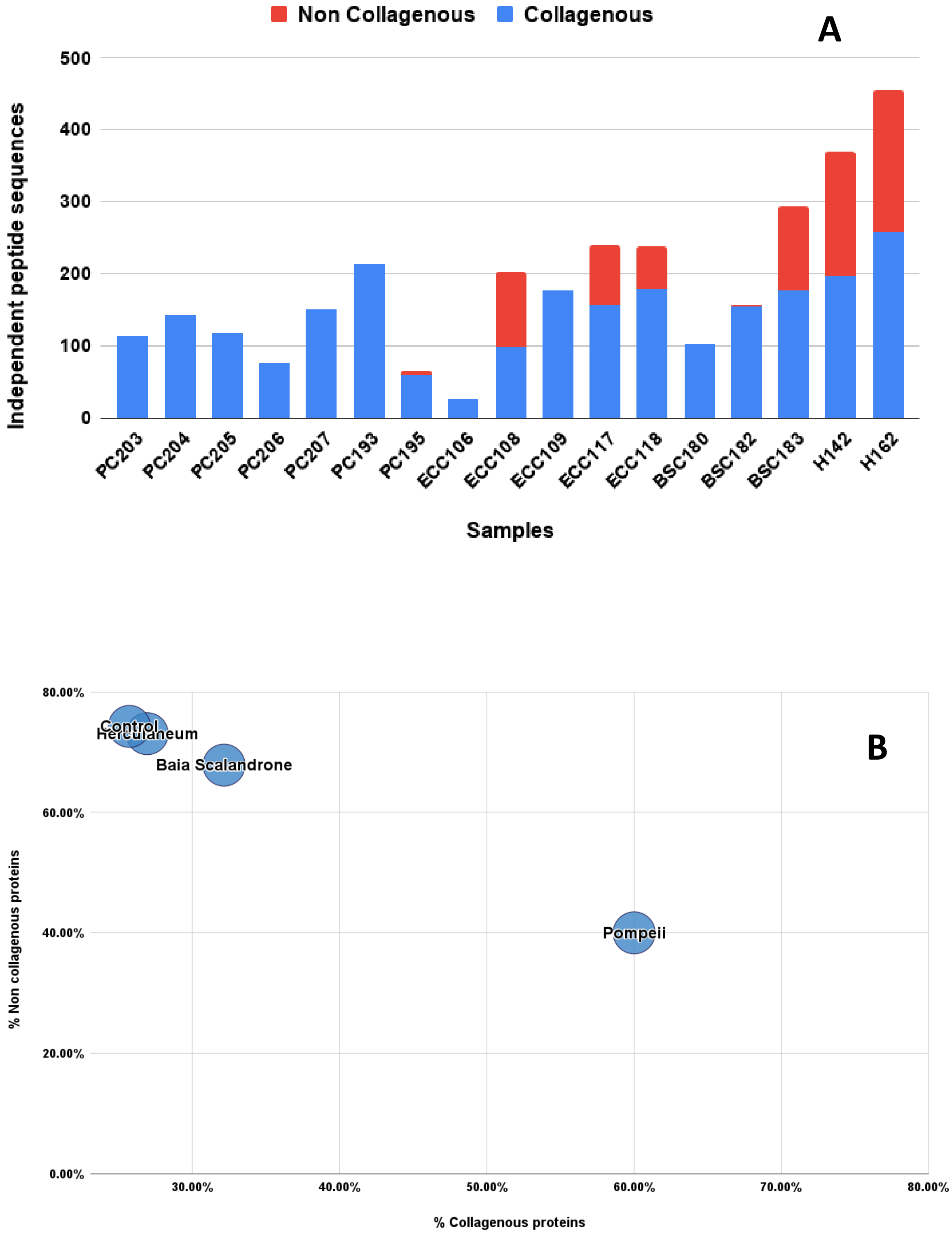

Fig. S3. Protein identification analysis. A) Number of independent peptides of collagenous (blue) and non-collagenous (red) proteins in each single sample. B) Grouping samples with respect to collagenous and non-collagenous protein content for each group.

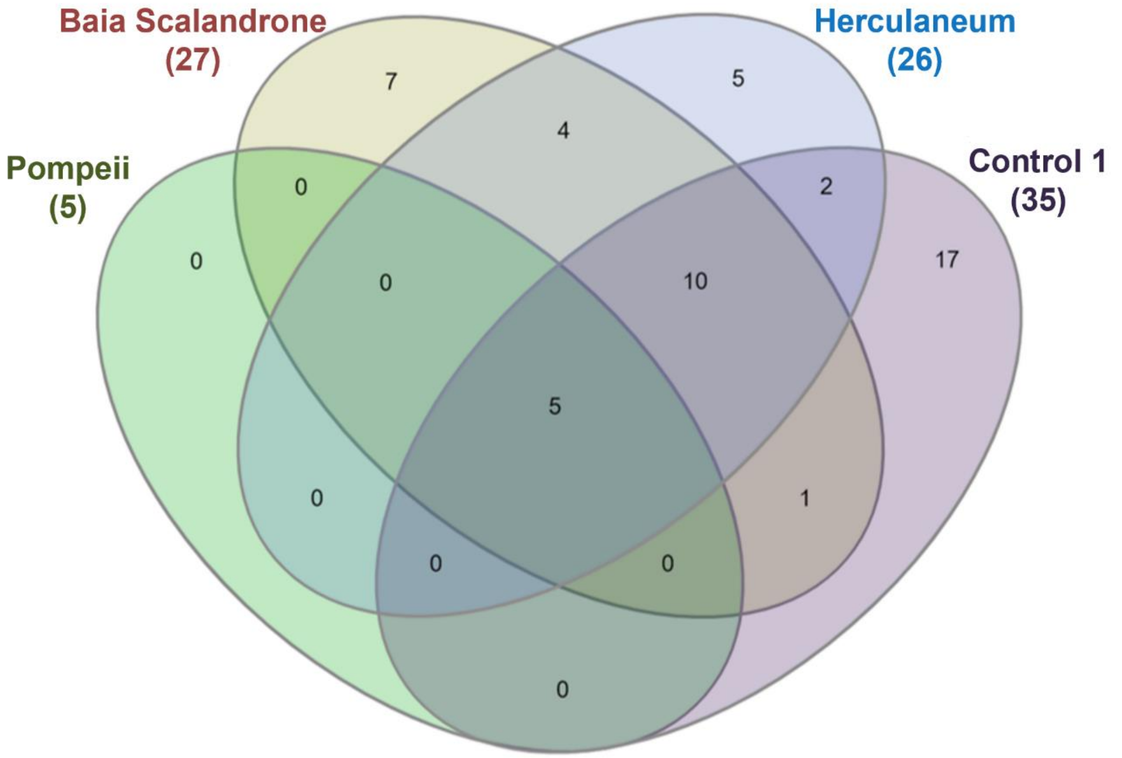

Fig. S4. Venn diagram of proteins that are shared among the groups of Pompeii, Herculaneum, Baia Scalandrone, and control (H-162, H-142 (*34*)) (http://www.interactivenn.net/).

Table S4. Unique and shared proteins between the different sample groups.

Group	Number of Proteins	Unique proteins
Herculaneum	5	Hemoglobin subunit beta, Periostin, Collagen alpha1(III) chain, NHS like protein 2, Osteocalcin
Baia Scalandrone	7	Collagen alpha-1(IX) chain, Collagen alpha-2(XI) chain, Fibronectin, Cartilage oligomeric matrix protein, Matrilin-3, Collagen alpha-1(X) chain, Collagen alpha-1(XI) chain

Control	17	Collagen alpha-3(VI) chain, Apolipoprotein E, Coagulation factor X, Insulin-like growth factor II, Cathepsin G, Matrix Gla Protein, Eosinophil peroxidase, Bone marrow proteoglycan, Collagen alpha-1(V) chain, Insulin-like growth factor-binding protein 5, Neutrophil defensin 3, Sushi repeat-containing protein SRPX, Collagen alpha-1(VII) chain, Dermatopontin

**Groups**	**Number of Proteins**	**Shared Proteins**
Herculaneum<br>Pompeii<br>Baia Scalandrone<br>Control	5	Collagen alpha-2(I) chain, Chondroadherin, Collagen alpha-1(I) chain, Collagen alpha-1(II) chain, Biglycan
Herculaneum<br>Baia Scalandrone	20	Alpha-2-HS-glycoprotein, Antithrombin-III, Basement membrane-specific heparan sulfate proteoglycan core protein, Biglycan, Chondroadherin, Coagulation factor IX, Collagen alpha-1(I), Collagen alpha-1(II), Collagen alpha-1(XII), Collagen alpha-1(XXII), Collagen alpha-2(I), Collagen alpha-2(V), Decorin, Kininogen-1, Lumican, Osteomodulin, Pigment epithelium-derived factor, Protein Z-dependent protease inhibitor, Prothrombin, Vitronectin
Herculaneum<br>Pompeii	6	Collagen alpha-1(I), Collagen alpha-2(I), Collagen alpha-1(II), Chondroadherin, Biglycan, Collagen alpha-2(V)
Herculaneum<br>Control	20	Alpha-2-HS-glycoprotein, Basement membrane-specific heparan sulfate proteoglycan core protein, Biglycan, Chondroadherin, Coagulation factor IX, Collagen alpha1(I) chain, Collagen alpha1(II) chain, Collagen alpha1(XII) chain, Collagen alpha1(XXII) chain, Collagen alpha2(I) chain, Collagen alpha2(V) chain, Decorin, Kininogen, Lumican, Olfactomedin like protein 3, Osteomodulin, Pigment epithelium derived factor, Protein Z dependent protease inhibitor, Prothrombin, Vitronectin
Baia Scalandrone<br>Control	20	Alpha-2-HS-glycoprotein, Basement membrane-specific heparan sulfate proteoglycan core protein, Biglycan, Chondroadherin, Coagulation factor IX, Coagulation factor VII, Collagen alpha-1(I), Collagen alpha-1(II), Collagen alpha-1(XII), Collagen alpha-1(XXII), Collagen alpha-2(I), Decorin, Kininogen-1, Lumican, Osteomodulin, Pigment epithelium-derived factor, Protein Z-dependent protease inhibitor, Prothrombin, Vitronectin, Coagulation factor-VII

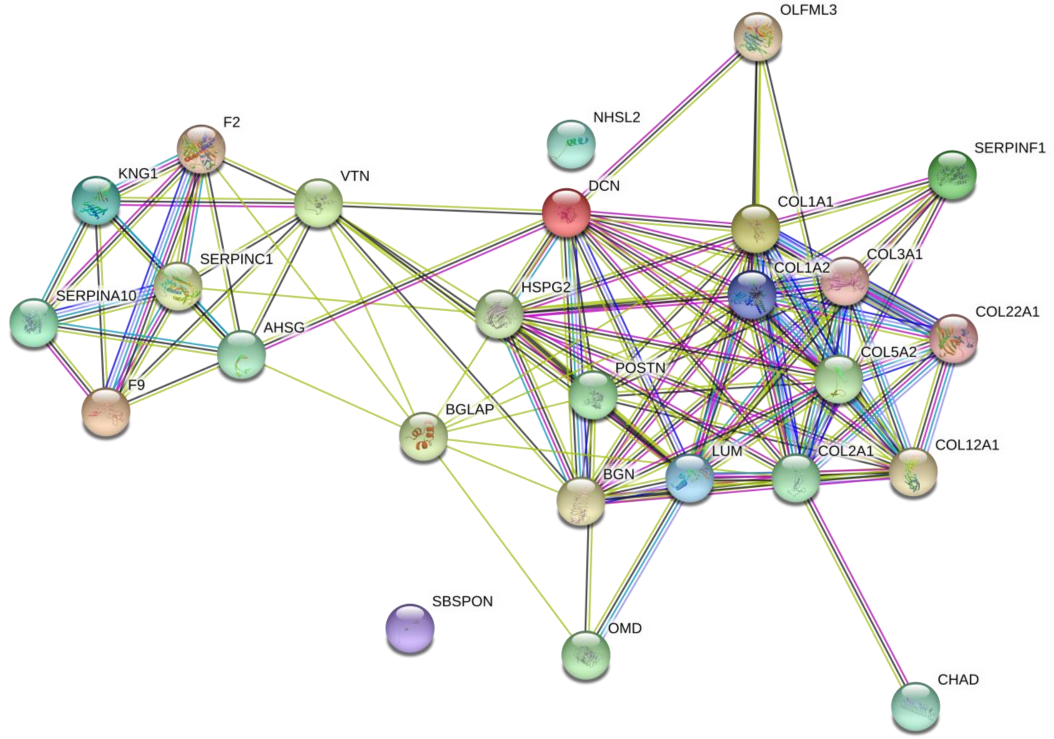

Abbreviation	Protein Name
POSTN	Periostin
VTN	Vitronectin
HBB	Hemoglobin subunit beta
BGLAP	Osteocalcin
BGN	Biglycan
SERPINC1	Antithrombin-III
DCN	Decorin
SERPINF1	Pigment epithelium-derived factor
COL1A1	Collagen alpha-1(I) chain
COL1A2	Collagen alpha-2(I) chain
COL3A1	Collagen alpha-1(III) chain
COL2A1	Collagen alpha-1(II) chain
CHAD	Chondroadherin
COL22A1	Collagen alpha-1(XXII) chain
COL5A2	Collagen alpha-2(V) chain
OMD	Osteomodulin
COL12A1	Collagen alpha-1(XII) chain
KNG1	Kininogen-1
LUM	Lumican
F2	Prothrombin
HSPG2	Basement membrane-specific heparan sulfate proteoglycan core protein

AHSG	Alpha-2-HS-glycoprotein
F9	Coagulation factor IX
ITGB1	Integrin beta-1
OLFML3	Olfactomedin-like protein 3
NHSL2	NHS-like protein 2
SERPINA10	Protein Z-dependent protease inhibitor

Fig. S5. STRING interaction pathway of bone proteins. Functional association network of proteins identified in skeletal bone samples from Pompeii, Herculaneum and Baia Scalandrone archaeological sites (https://string-db.org/).

Deamidation (N, Q)

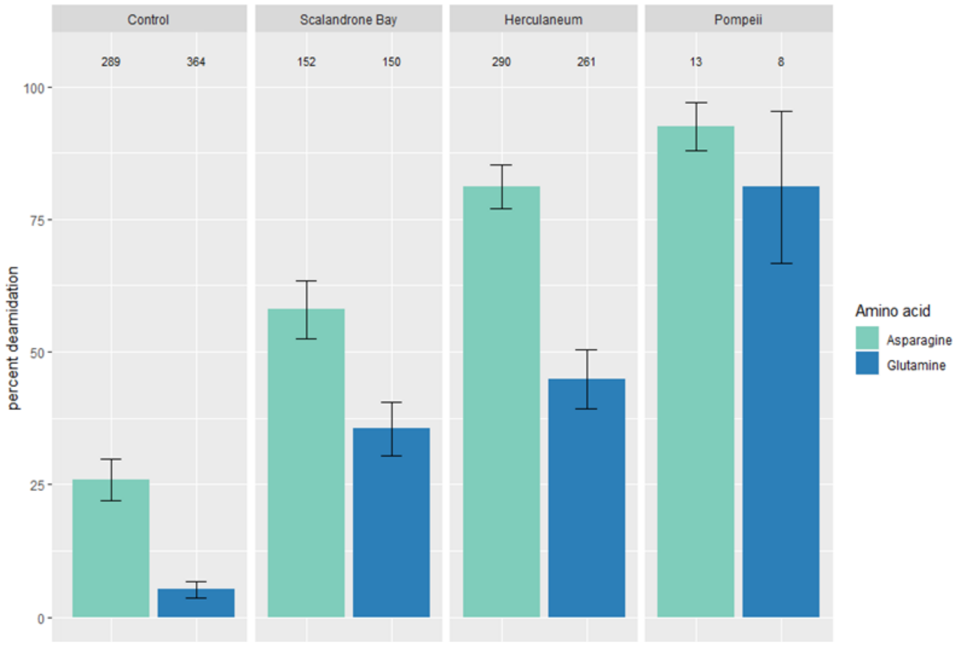

Fig. S6. Overall percentage of deamidation (N, Q) of non-collagenous bone proteins from Pompeii, Herculaneum, Baia Scalandrone and control (H-162, H-142 (*34*)) samples. Error bars represent standard deviation and numbers above each bar represent the number of deamidation sites the data is based on.

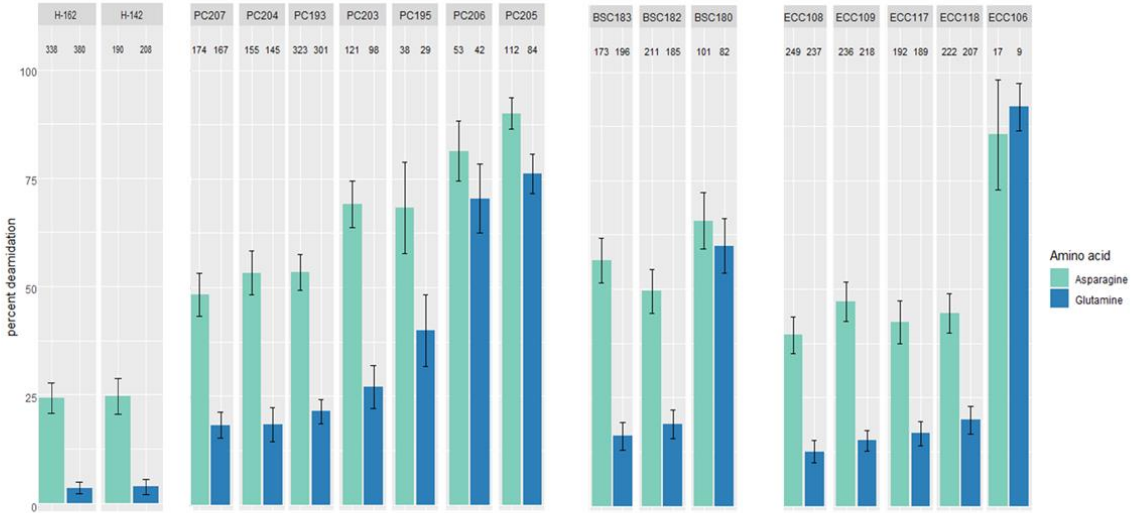

Fig. S7. Percentage of deamidation (N, Q) of collagenous proteins in bone samples from Pompeii, Herculaneum, Baia Scalandrone and control (H-162-H-142 (*34*)). Error bars represent standard deviation, and numbers above each bar represent the number of peptides the data is based on.

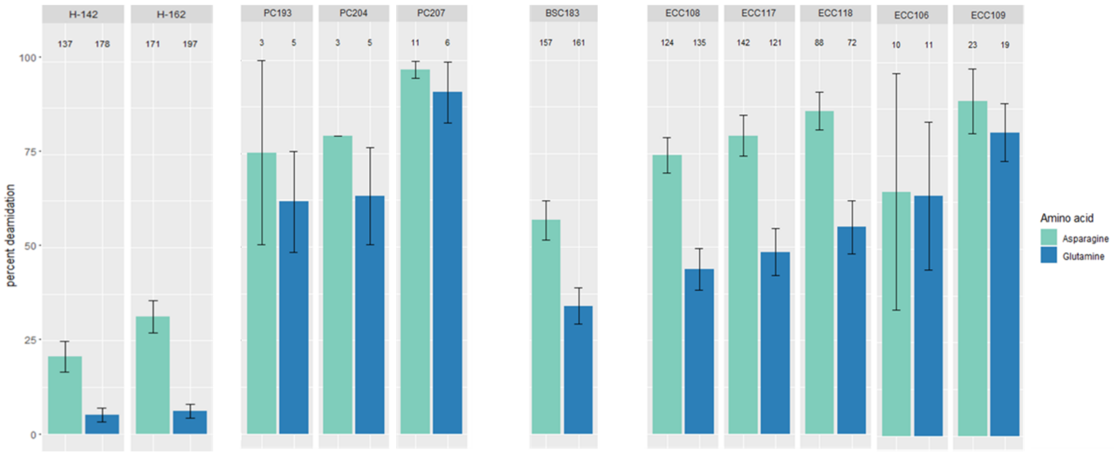

Fig. S8. Percentage of deamidation (N, Q) of non-collagenous proteins in bone samples from Pompeii, Herculaneum, Baia Scalandrone and control (H-162-H-142 (*34*)). Error bars represent standard deviation, and numbers above each bar represent the number of peptides the data is based on.

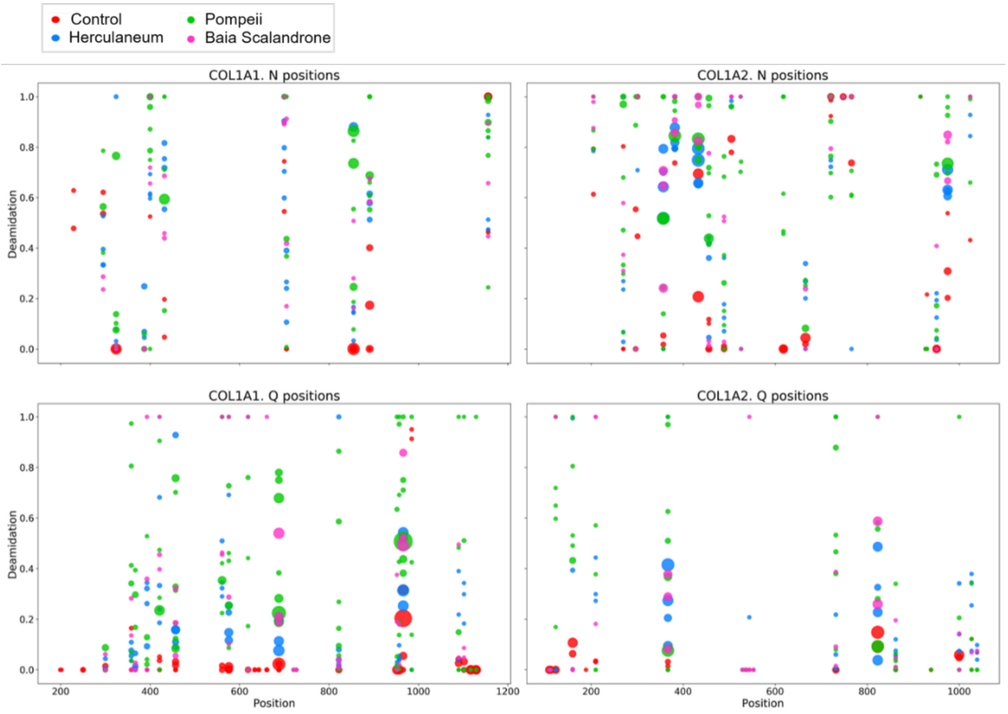

Fig. S9. Visualization of deamidation level (N, Q) along the sequence of collagen alpha-1 (I) (upper panel) and alpha-2 (I) (lower panel), in the bone samples from Pompeii, Herculaneum, Baia Scalandrone and control (H-162, H-142 (*34*)).

Oxidation (M)

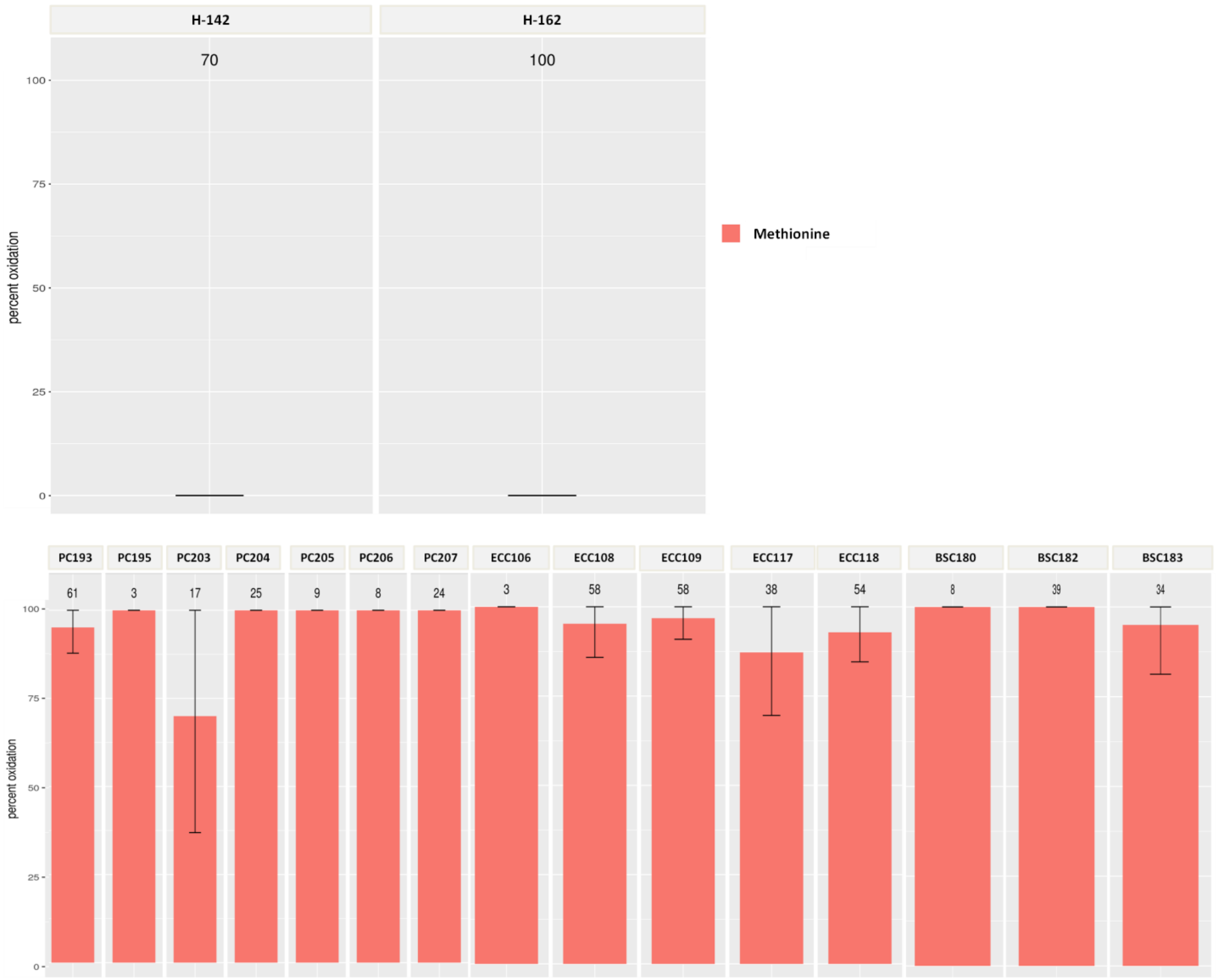

Fig. S10. Percentage of oxidation (M) of collagenous proteins in bone samples from Pompeii, Herculaneum, Baia Scalandrone and control (H-162-H-142 (*34*)). Error bars represent standard deviation, and numbers above each bar represent the number of peptides the data is based on.

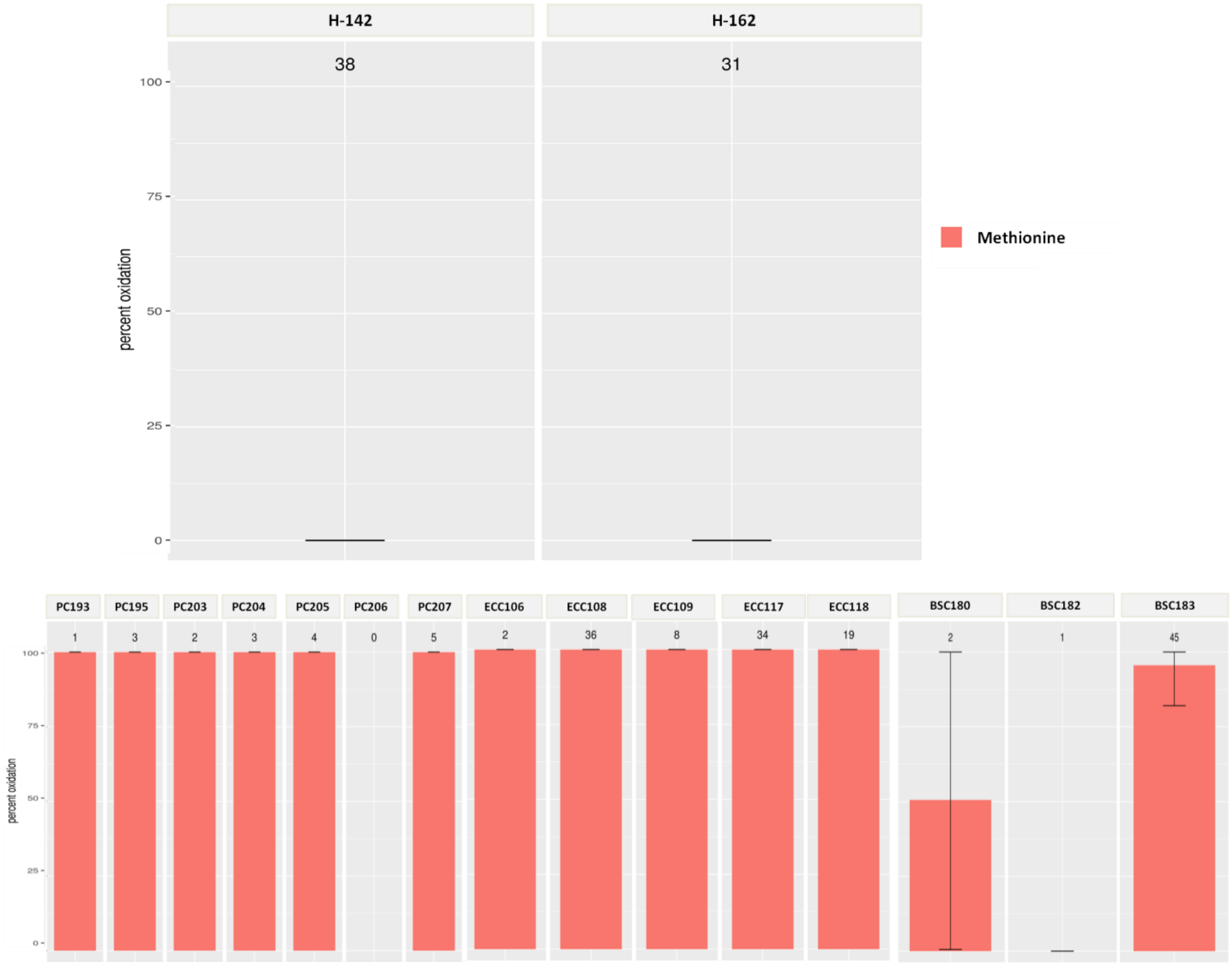

Fig. S11. Percentage of oxidation (M) of non-collagenous proteins in bone samples from Pompeii, Herculaneum, Baia Scalandrone and control (H-162-H-142 (*34*)). Error bars represent standard deviation, and numbers above each bar represent the number of peptides the data is based on.

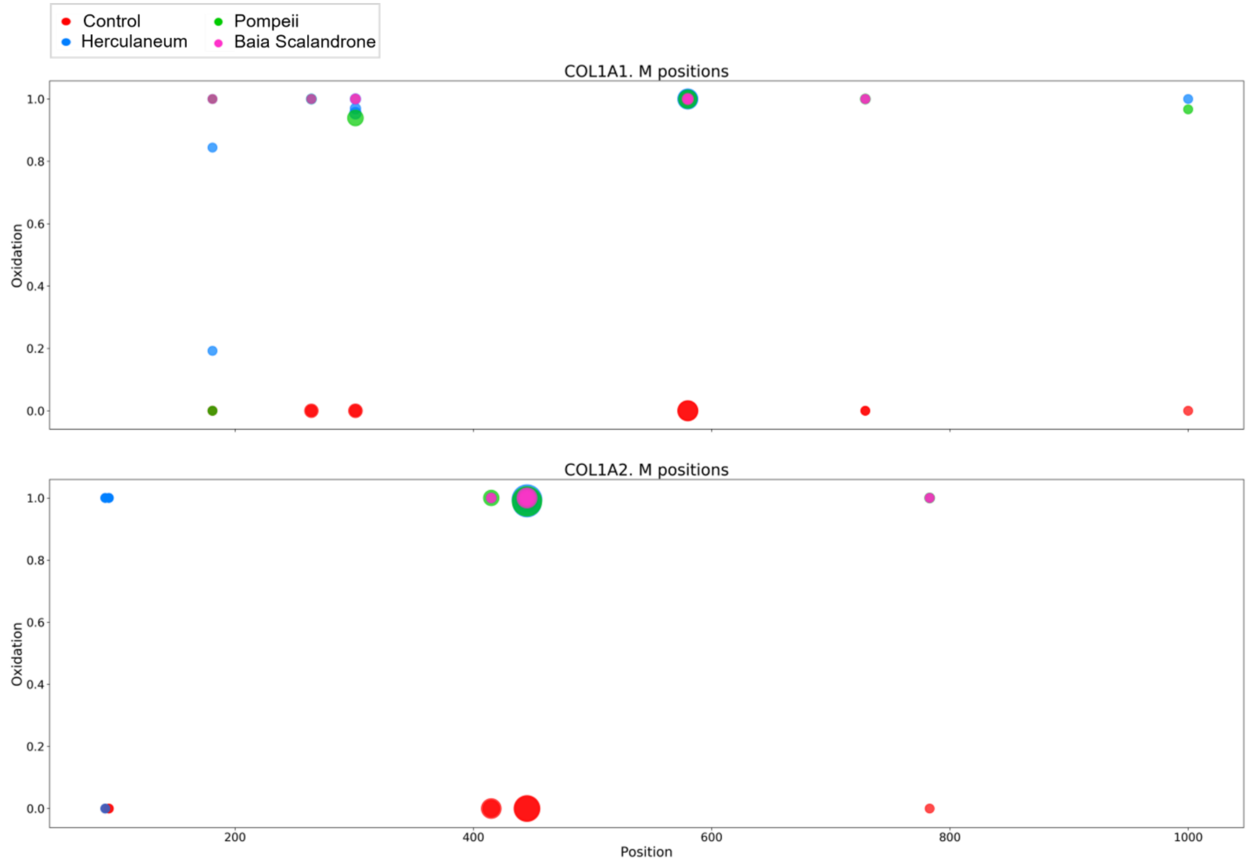

Fig. S12. Visualization of oxidation level (M) along the sequence of collagen alpha-1 (I) (upper panel) and alpha-2 (I) (lower panel) in the bone samples from Pompeii, Herculaneum, Baia Scalandrone, and control (H-162, H-142 (*34*)).

Non-enzymatic backbone cleavage

GSDGSVGPVGPA
GSDGSVGPVGPAG
GSDGSVGPVGPAGPIG
GSDGSVGPVGPAGPIGSA
GSDGSVGPVGPAGPIGSAG
GSDGSVGPVGPAGPIGSAGP
GSDGSVGPVGPAGPIGSAGPP
GSDGSVGPVGPAGPIGSAGPPG

Fig. S13. Example of the "pacman" effect observed in searching for the semitryptic peptides in order to visualize spontaneous backbone cleavages.

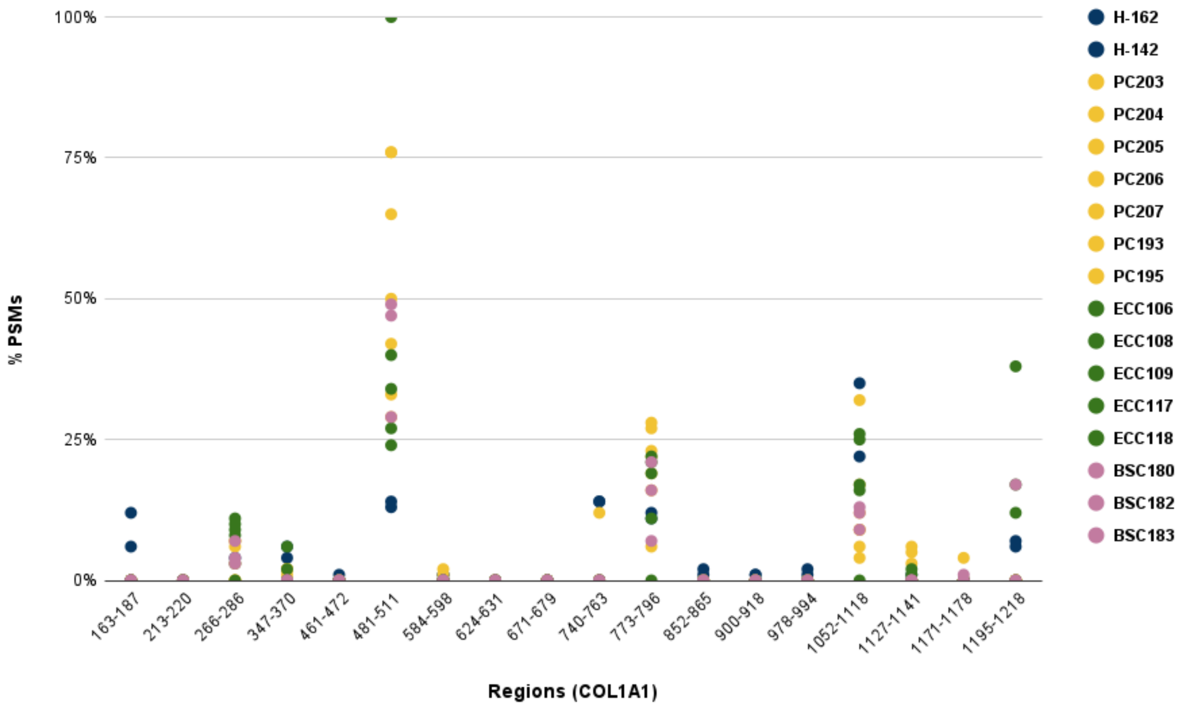

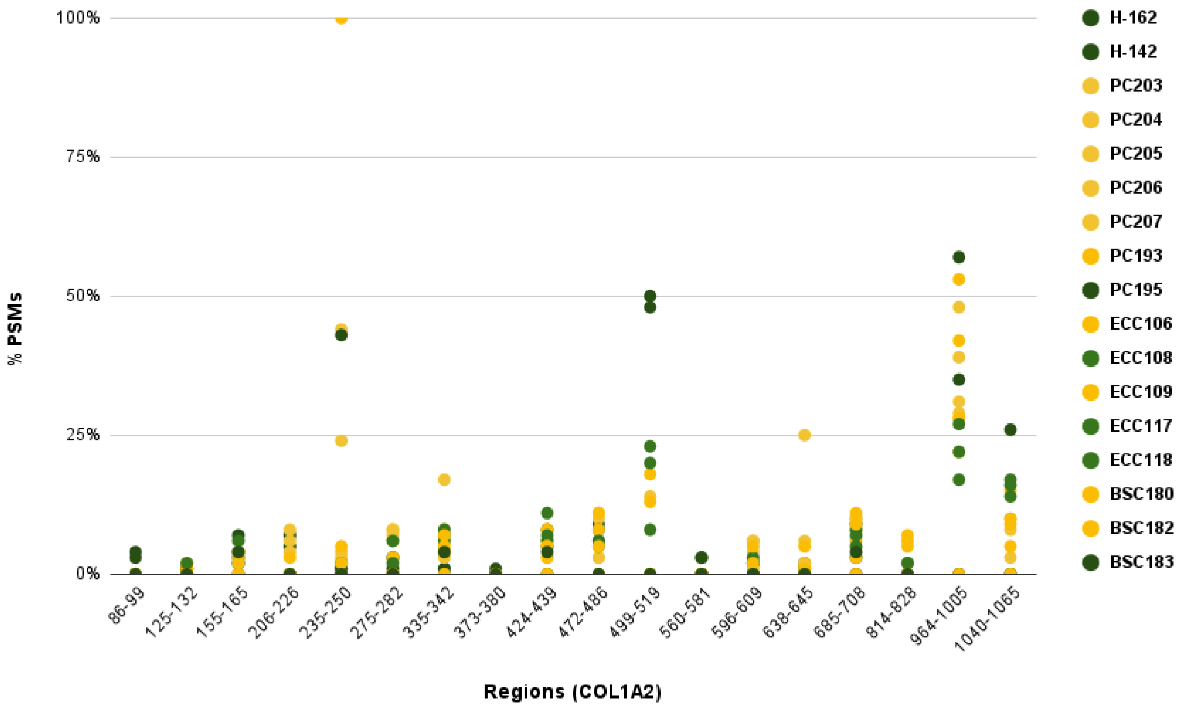

Fig. S14. Semi-quantitative evaluation of the backbone cleavage along the sequence of COL1A1 and COL1A2 chains on the basis of detection of semitryptic peptides.

Other diagenetically induced chemical modifications

Table S5 (A-E). Occurrence of chemical modifications within the dataset of the sample groups of Pompeii, Herculaneum, Baia Scalandrone and control (H162-H142 (*1*)).

A<br>Lysine Modification	ΔM	Pompeii	Herculaneum	Baia Scalandrone	Control
		**K positions** (mod/detected/theoretical number in the sequence of COL1A1 and COL1A2)			
**Carboxyethyl**	72.022	2/51/69	6/54/69	3/50/69	12/64/69
**Aminoadipic**	14.964	6/51/69	4/54/69	1/50/69	1//64/69
**Carboxymethyl**	58.006	6/51/69	7/54/69	3/50/69	1//64/69
**Formyl**	27.996	6/51/69	3/54/69	1/50/69	6//64/69

**B** **Arginine Modification**	**ΔM**	**Pompeii**	**Herculaneum**	**Baia Scalandrone**	**Control**
		**R positions** (mod/detected/theoretical number in the sequence of COL1A1 and COL1A2)			
**MG-H1**	54.011	9/87/108	8/88/108	3/91/108	4/103/108
**G-H1**	39.995	7/87/108	5/88/108	6/91/108	4//103/108
**Ornithine**	- 42.021	8/87/108	14/88/108	6/91/108	5//103/108

**C** **Proline Modification**	**ΔM**	**Pompeii**	**Herculaneum**	**Baia Scalandrone**	**Control**
		**P positions** (mod/detected/theoretical number in the sequence of COL1A1 and COL1A2)			
**Di-Oxidation**	31.989	30/344/449	42/306/449	28/328/449	21/372/449
**Tri-Oxidation**	47.983	10/344/449	19/306/449	8/328/449	10/372/449
**PyroGlu**	13.980	10/344/449	23/306/449	8/328/449	10/372/449
**Dhp**	-2.001	17/344/449	14/306/449	3/328/449	3/372/449

**D** **Histidine Modification**	**ΔM**	**Pompeii**	**Herculaneum**	**Baia Scalandrone**	**Control**
		**H positions** (mod/detected/theoretical number in the sequence of COL1A1 and COL1A2)			
**His->Asp**	-22.031	2/7/15	3/8/15	2/7/15	0/13/15
**Di-Oxidation**	31.989	2/7/15	3/8/15	0/7/15	1//13/15
**Oxidation**	15.995	2/7/15	2/8/15	1/7/15	2//13/15

**E** **Serine and Threonine Modification**	**ΔM**	**Pompeii**	**Herculaneum**	**Baia Scalandrone**	**Control**
		**S and T positions** (mod/detected/theoretical number in the sequence of COL1A1 and COL1A2)			
**S->G**	-30.010	3/54/71	3/53/71	0/51/71	2/62/71

**T->G**	-44.026	3/29/37	3/30/37	1/32/37	2/32/37

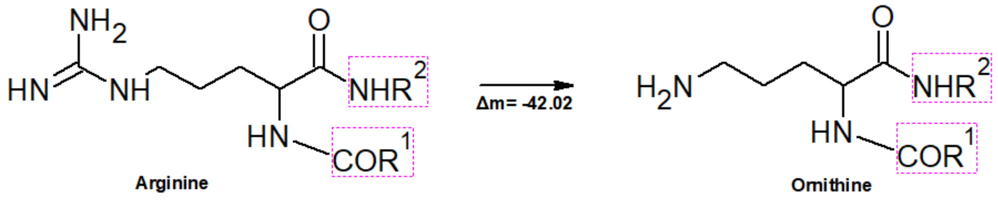

Fig. S15. Conversion of Arginine to Ornithine.

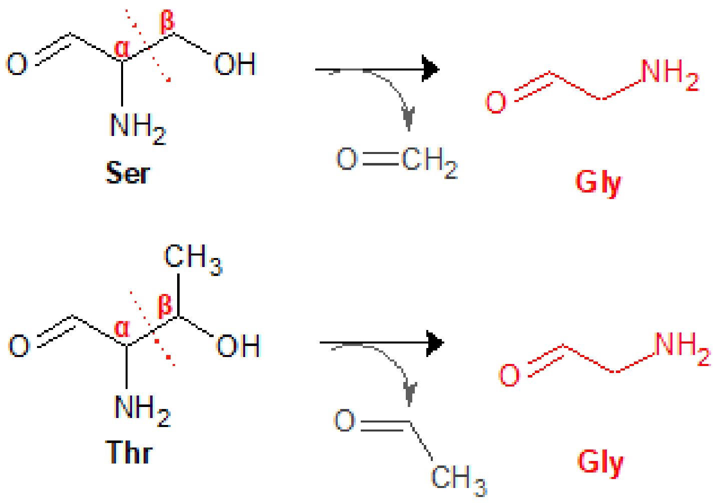

Fig. S16. Oxidation reaction at the side chain of Threonine (T) and Serine (S), leading to Cα-Cβ bond cleavage to Glycine (G).

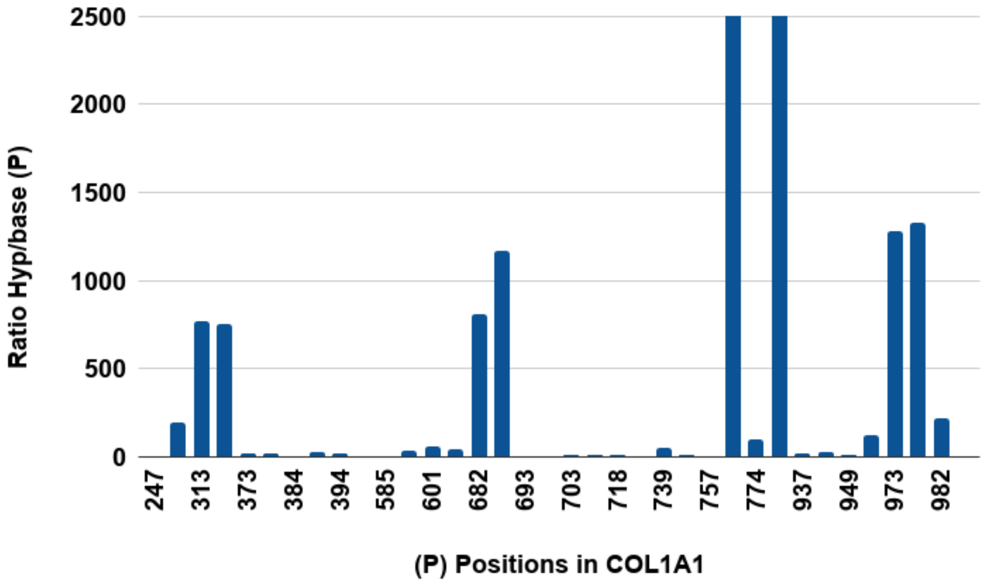

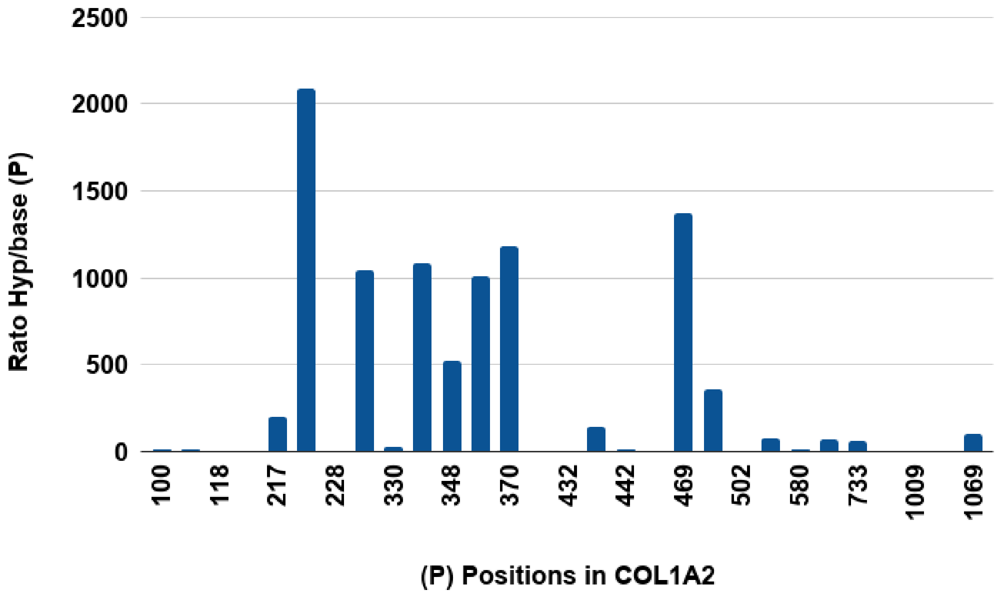

Fig. S17. Example of site-specific hydroxylation occupancy on Proline residues along the sequence of COL1A1 (left) and COL1A2 (right) of control samples (*34*). The modification occupancy at a specific primary structure position arises from the average ratio modified/non modified values extracted from the HydroxyprolineSites.txt files of MQ (M. Rykær, B. Svensson, M. J. Davies, P. Hägglund. Unrestricted Mass Spectrometric Data Analysis for Identification, Localization, and Quantification of Oxidative Protein Modifications. *J. Proteome Res.* **16**, 3978–3988 (2017)).

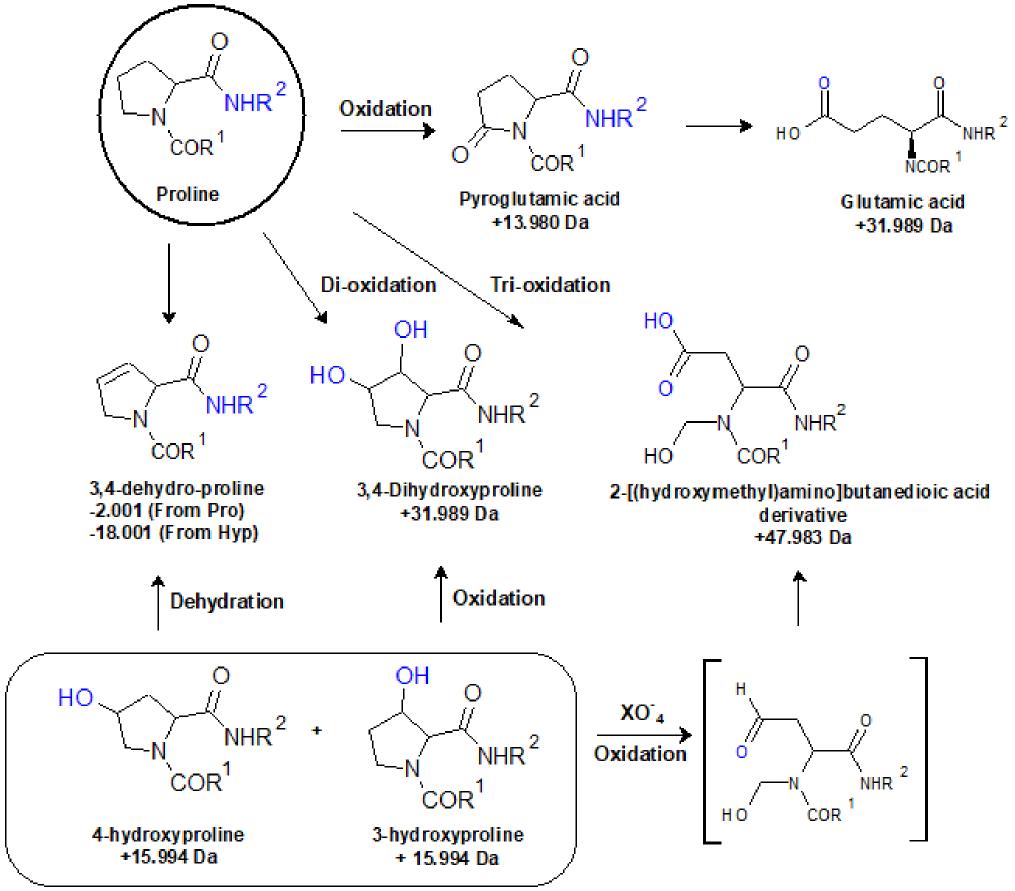

Fig. S18. Possible Proline oxidation products. Unless otherwise stated, ΔM values are calculated in respect to proline.

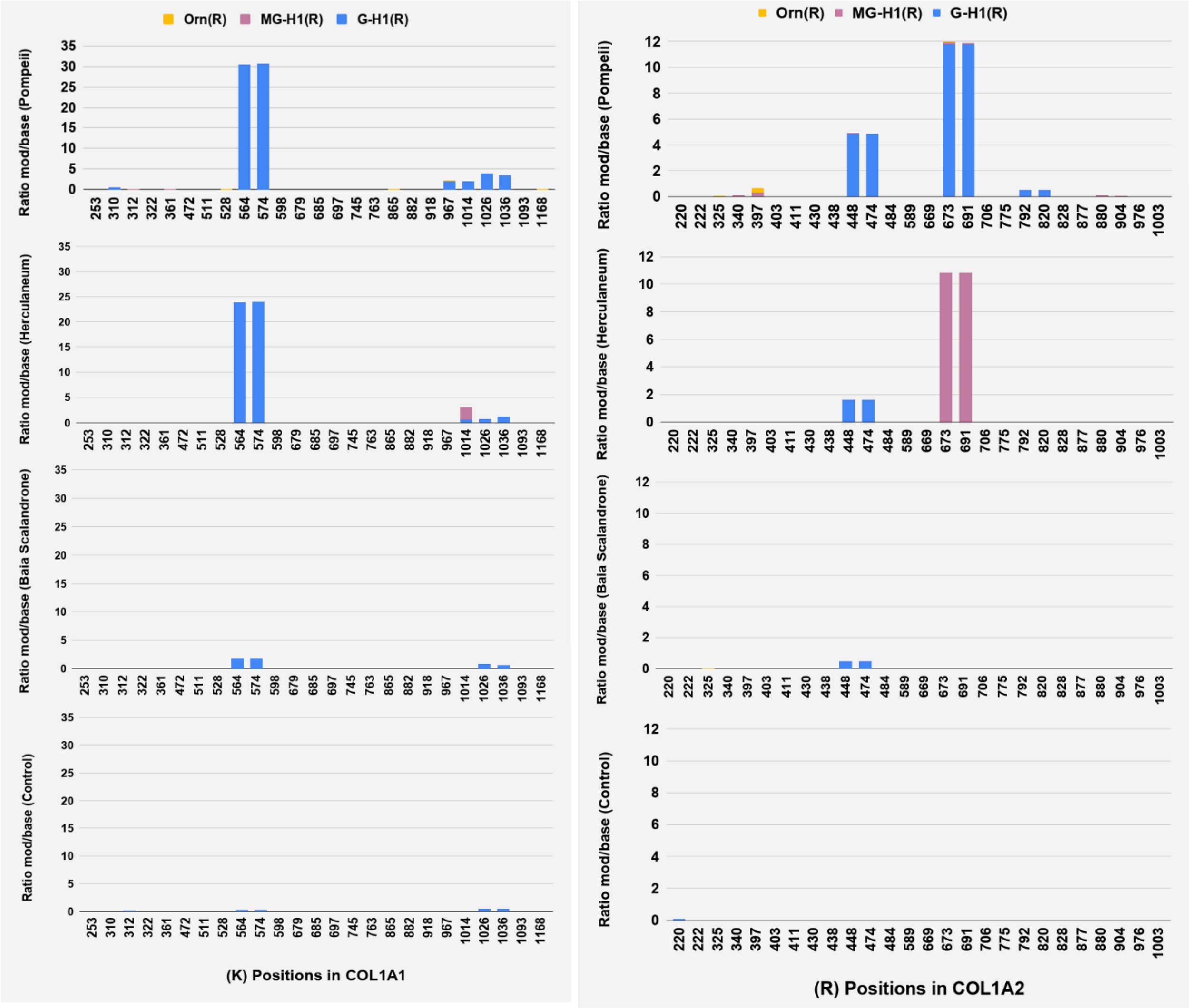

Fig. S19. Site-specific modification on Arginine residues along the sequence of COL1A1 (left) and COL1A2 (right). Methylglyoxal (MGH-1) and glyoxal (GH-1) derived hydroimidazolones, and conversion of Arginine to Ornithine (Orn) were semi-quantitatively calculated from the average modified/non modified values, extracted from the modificationSites.txt files of MQ (M. Rykær, B. Svensson, M. J. Davies, P. Hägglund. Unrestricted Mass Spectrometric Data Analysis for Identification, Localization, and Quantification of Oxidative Protein Modifications. *J. Proteome Res.* **16**, 3978–3988 (2017)).

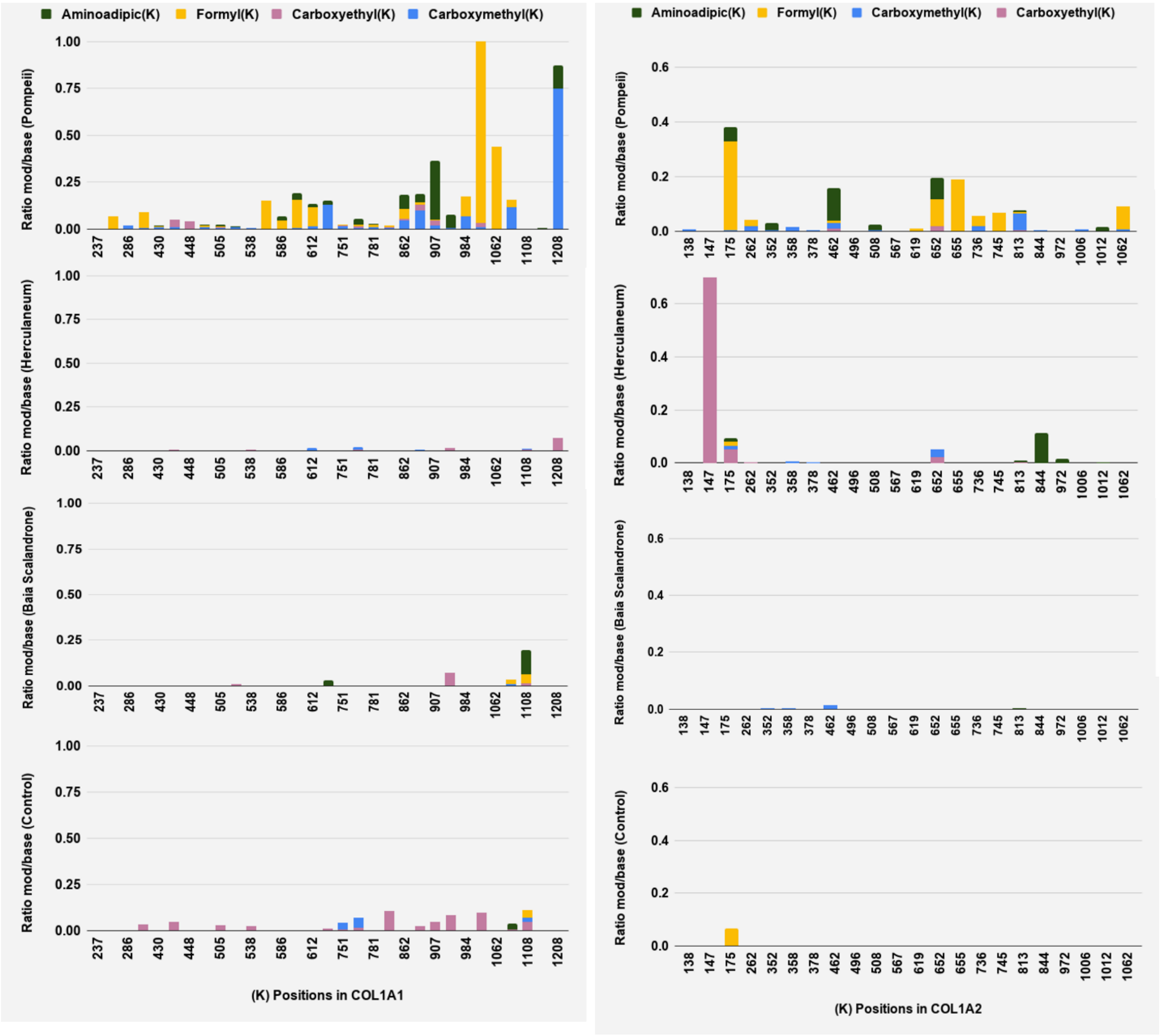

Fig. S20. Site-specific modification on Lysine residues along the sequence of COL1A1 (left) and COL1A2 (right). Aminoadipic, Formyl, Carboxyethyl, and Carboxymethyl modifications on Lysines were semi-quantitatively calculated from the average modified/non modified values, extracted from the modificationSites.txt files of MQ (M. Rykær, B. Svensson, M. J. Davies, P. Hägglund. Unrestricted Mass Spectrometric Data Analysis for Identification, Localization, and Quantification of Oxidative Protein Modifications. *J. Proteome Res.* **16**, 3978–3988 (2017)).

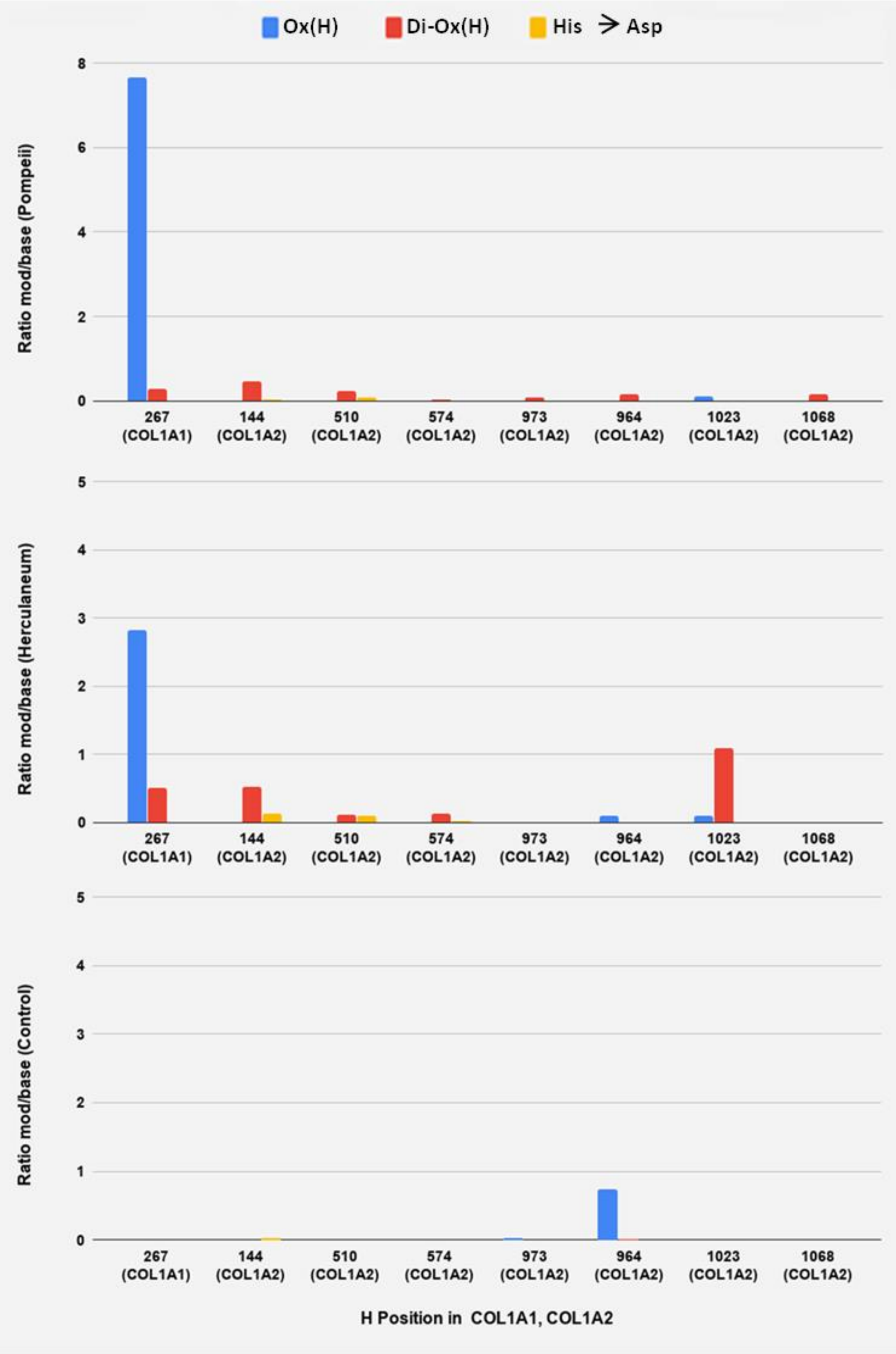

Fig. S21. Site specific modification on Histidine residues along the sequence of COL1A1 and COL1A2. Oxidation (Ox), Di-oxidatio (Di-Ox) and conversion of Histidine to Aspartic were semi-quantitatively calculated from the average modified/non modified values, extracted from the modificationSites.txt files of MQ (M. Rykær, B. Svensson, M. J. Davies, P. Hägglund. Unrestricted Mass Spectrometric Data Analysis for Identification, Localization, and Quantification of Oxidative Protein Modifications. *J. Proteome Res.* **16**, 3978–3988 (2017)).

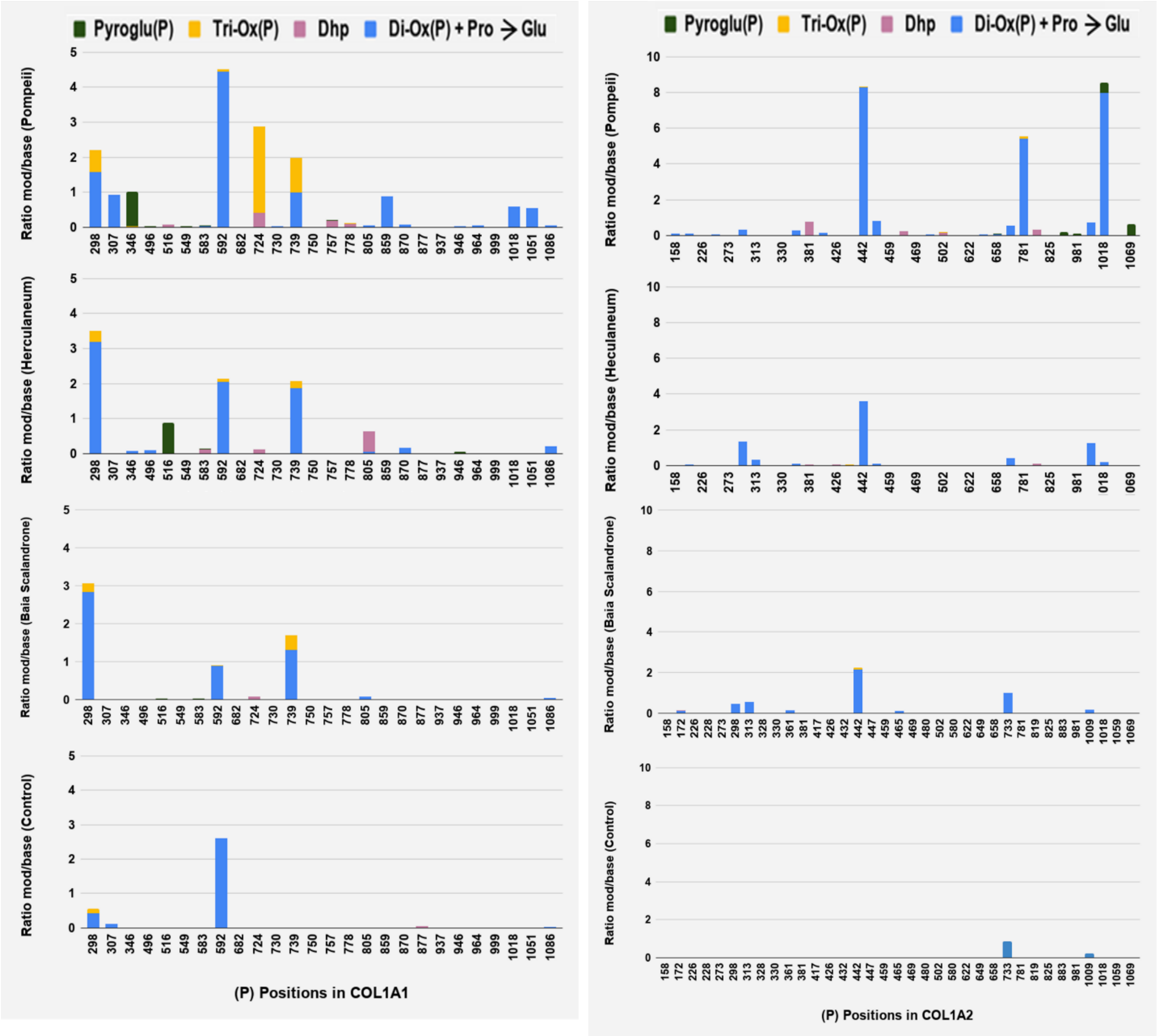

Fig. S22. Primary structure modification occupancy on Proline residues along the sequence of COL1A1 (left) and COL1A2 (right). The modification occupancy at a specific primary structure position was semi-quantitatively calculated from the average modified/non modified values, extracted from the modificationSites.txt files of MQ (M. Rykær, B. Svensson, M. J. Davies, P. Hägglund. Unrestricted Mass Spectrometric Data Analysis for Identification, Localization, and Quantification of Oxidative Protein Modifications. *J. Proteome Res.* **16**, 3978–3988 (2017)).

Selected MS/MS spectra

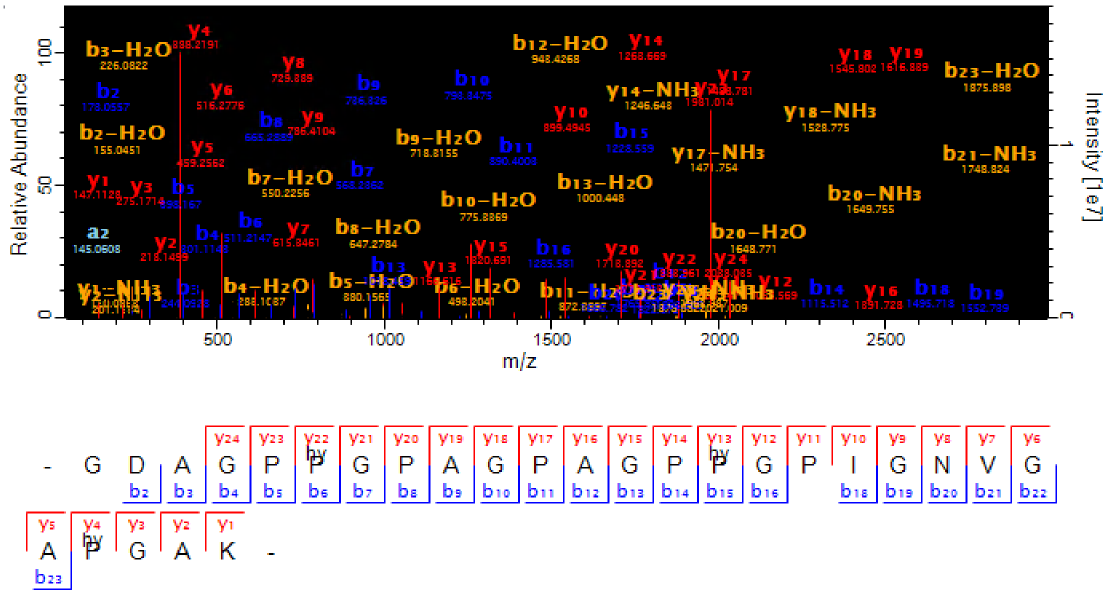

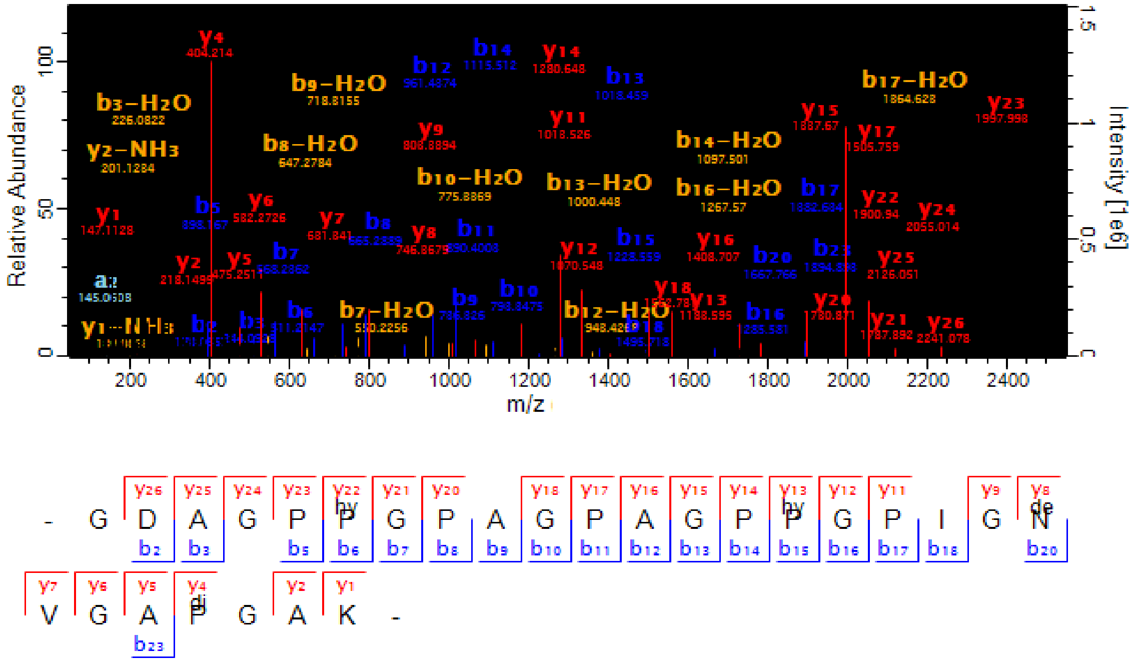

Fig. S23 (a-b). MS/MS Spectra of COL1A1 GDAGPPGPAGPAGPPGPIGNVGAPGAK peptide. The underlined Proline is detected as hydroxylated in the upper spectrum and di-oxidated (ΔM 31.989) in the lower spectrum.

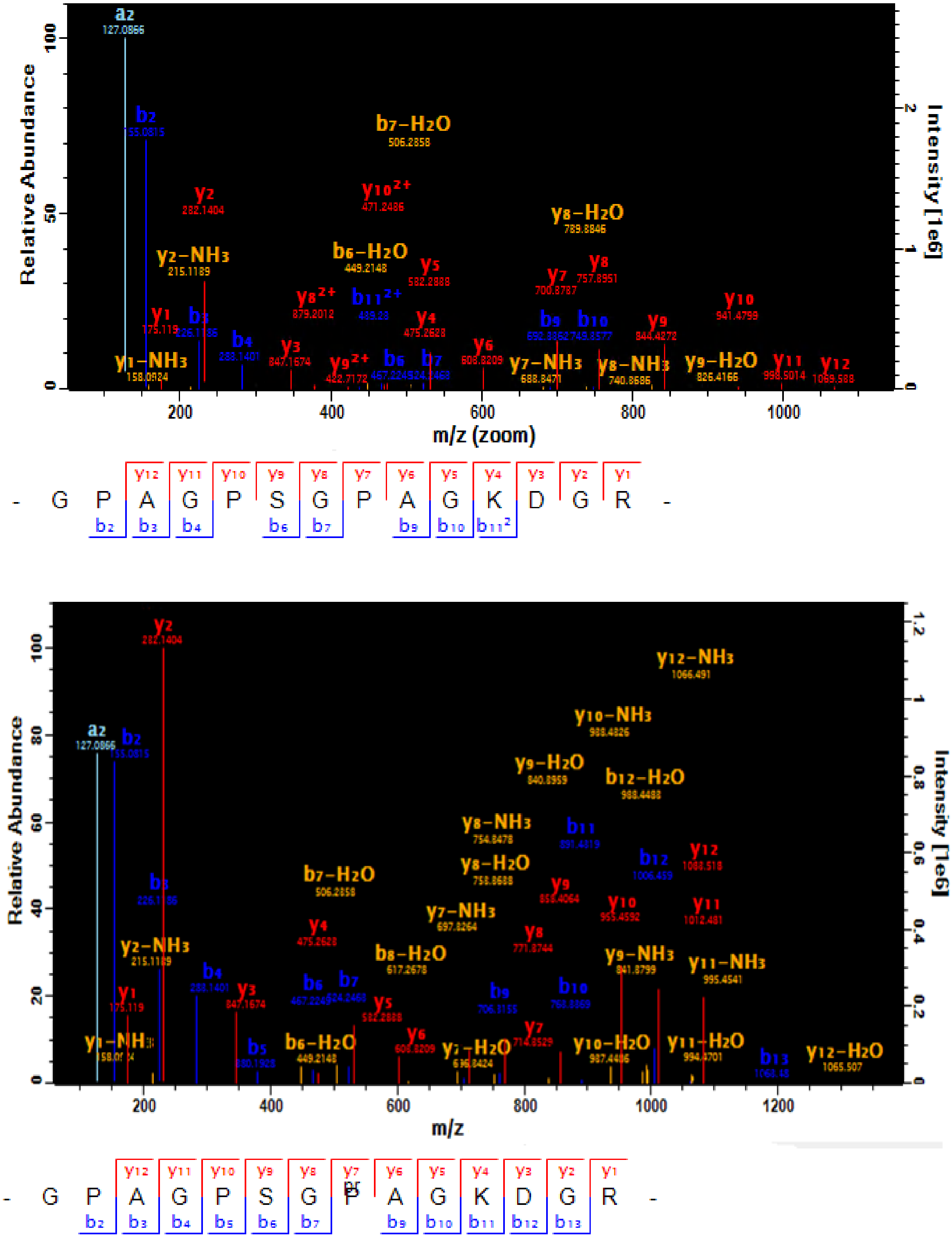

Fig. S24 (a-b). MS/MS Spectra of COL1A2 GPAGPSGPAGKDGR peptide. The underlined Proline is detected as not modified in the upper spectrum and as oxidized to pyroglutamic acid (ΔM 13.979) in the lower spectrum.

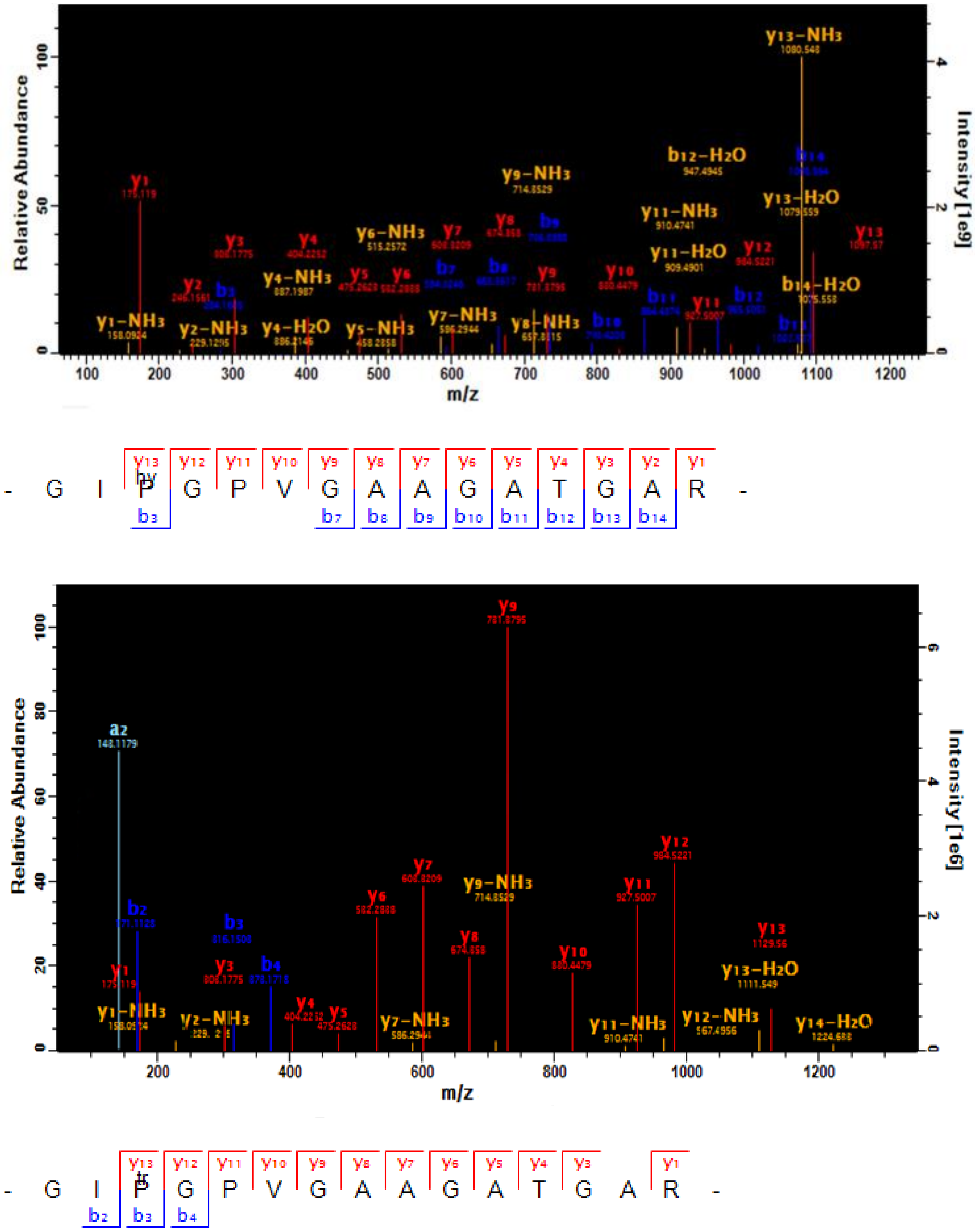

Fig. S25 (a-b). MS/MS Spectra of COL1A2 GIPGPVGAAGATGAR peptide. The underlined Proline is detected as hydroxylated in the upper spectra and as tri-oxidized (ΔM 47.985) in the lower spectrum.

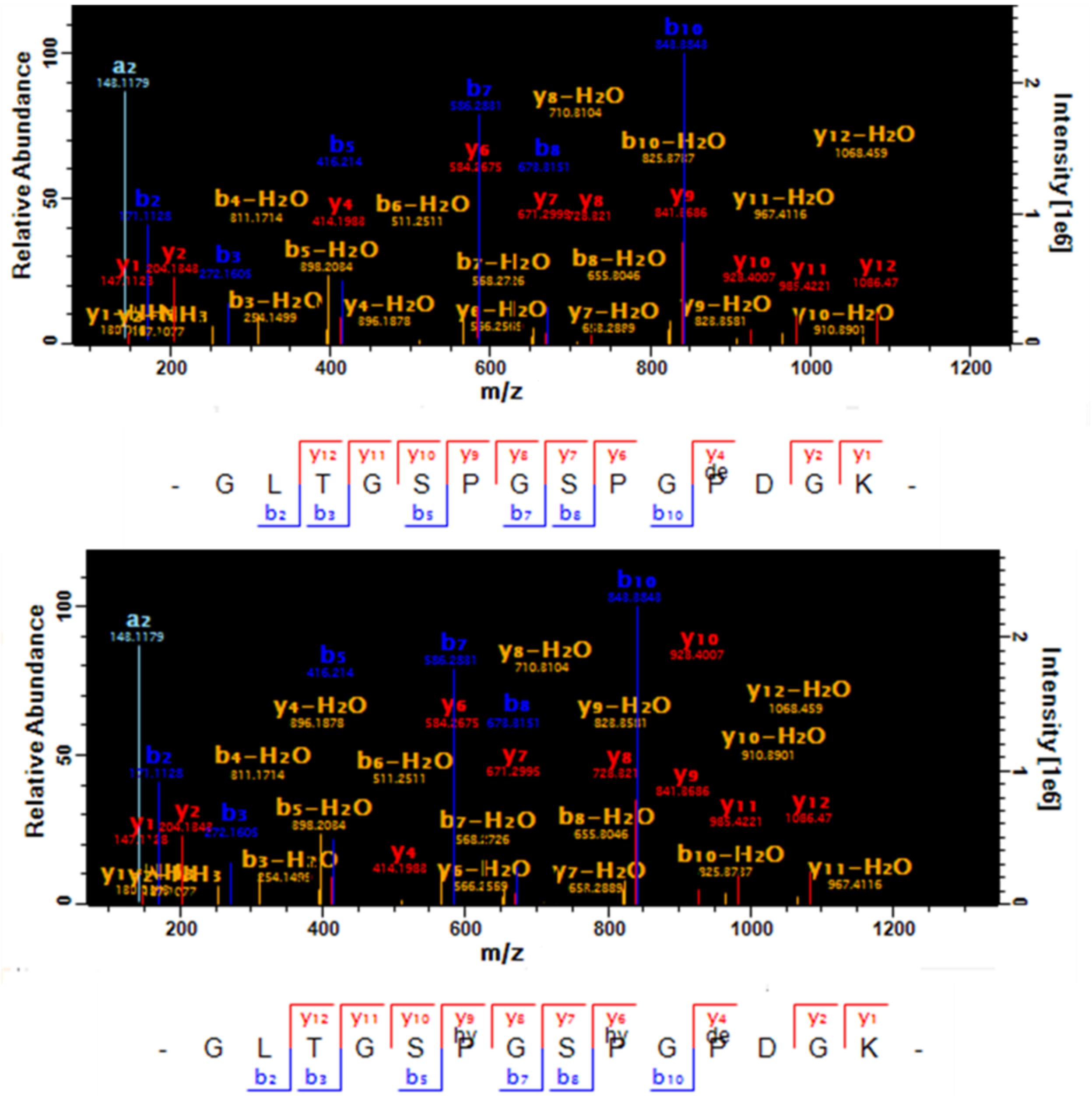

Fig. S26 (a-b). MS/MS Spectra of COL1A1 GLTGSPGSPGPDGK peptide. The underlined Proline is detected in the upper spectra where hydroxylation (P) was set as variable modification with a mass shift of -2.016DA. When hydroxylation (P) was set as fixed modification (lower spectra), the underlined P was detected with a mass shift of -18.010 Da.

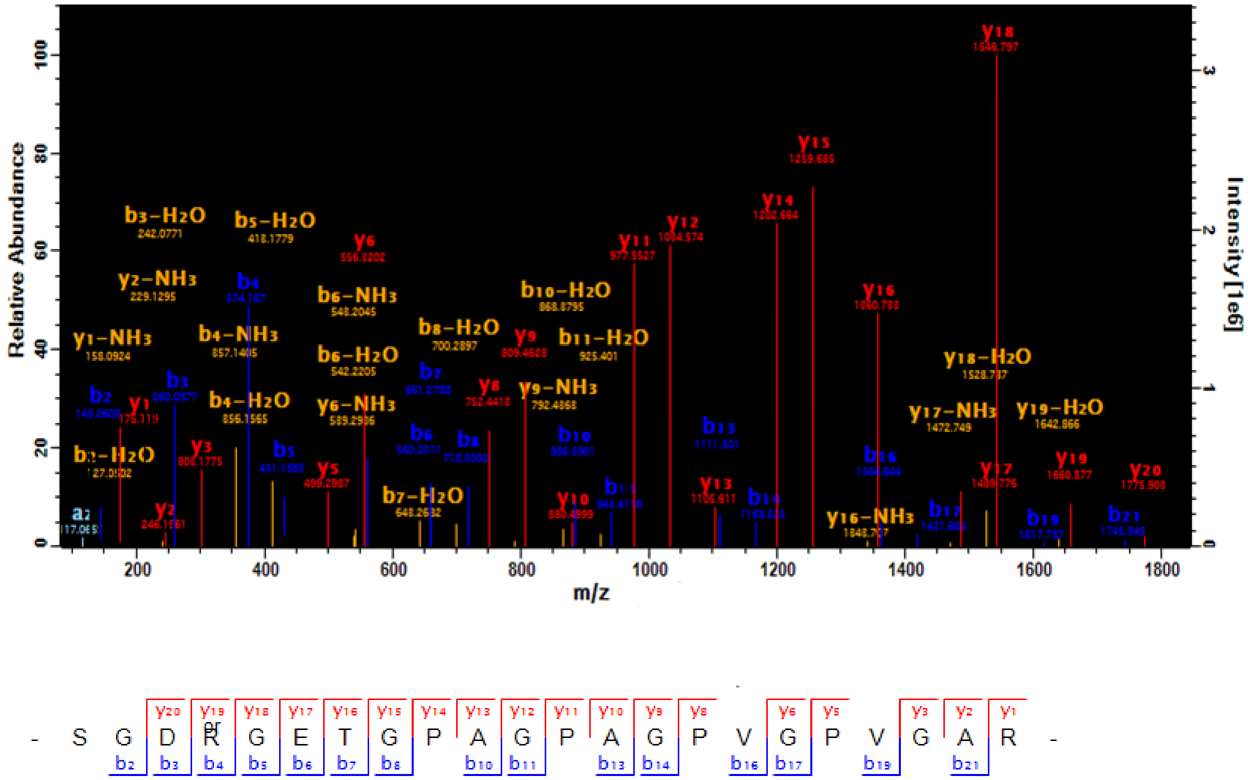

Fig. S27. MS/MS Spectra of COL1A2 SGDRGETGPAGPAGPVGPVGAR peptide. The underlined Arginine is detected as ornithine (ΔM - 42.022).

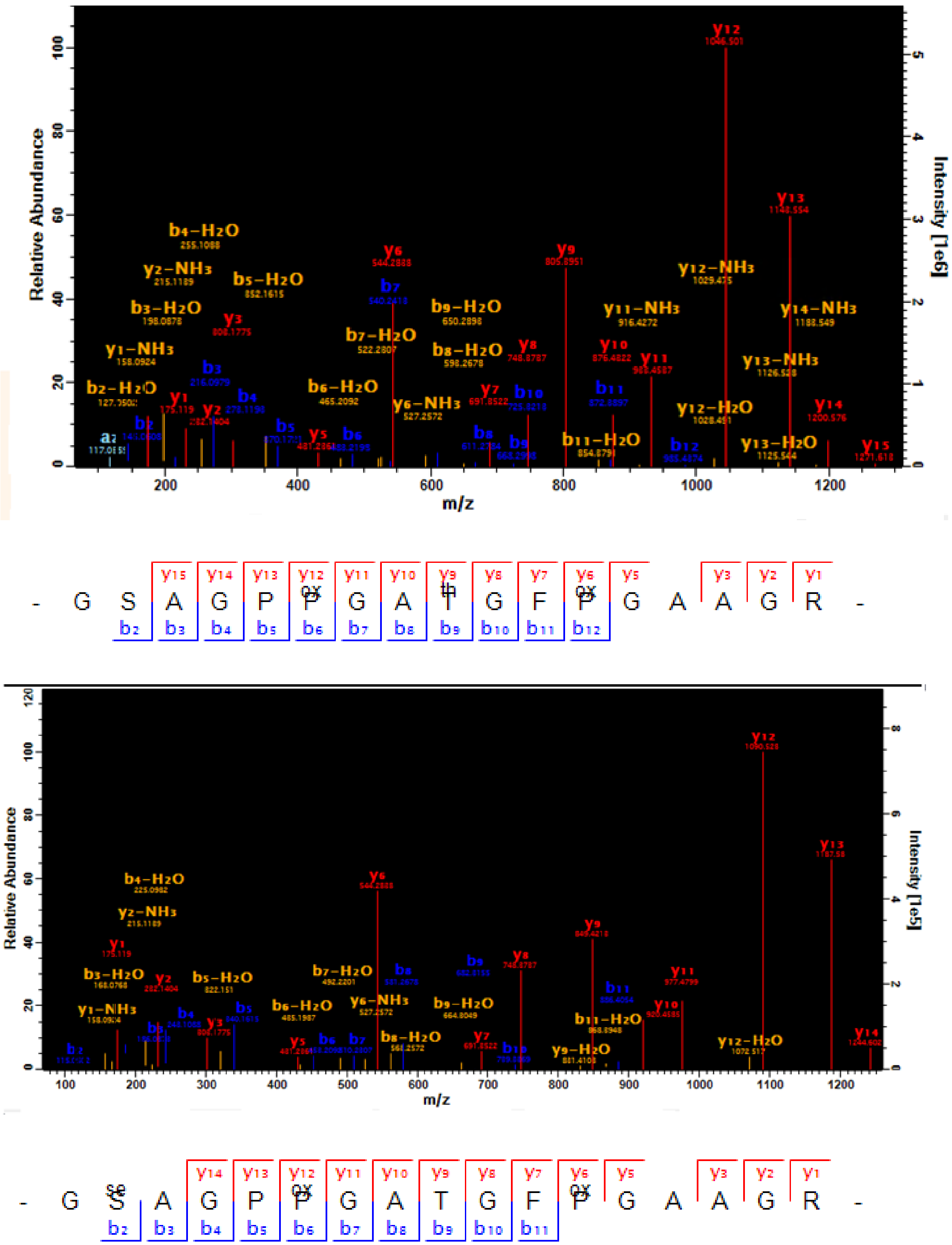

Fig. S28 (a-b). MS/MS Spectra of COL1A1 GSAGPPGATGFPGAAGR peptide. The spectra demonstrate a Threonine → Glycine (ΔM -30.010) and a Serine → Glycine (ΔM -44.026) substitution, respectively. The peptide with unmodified residues was also identified.

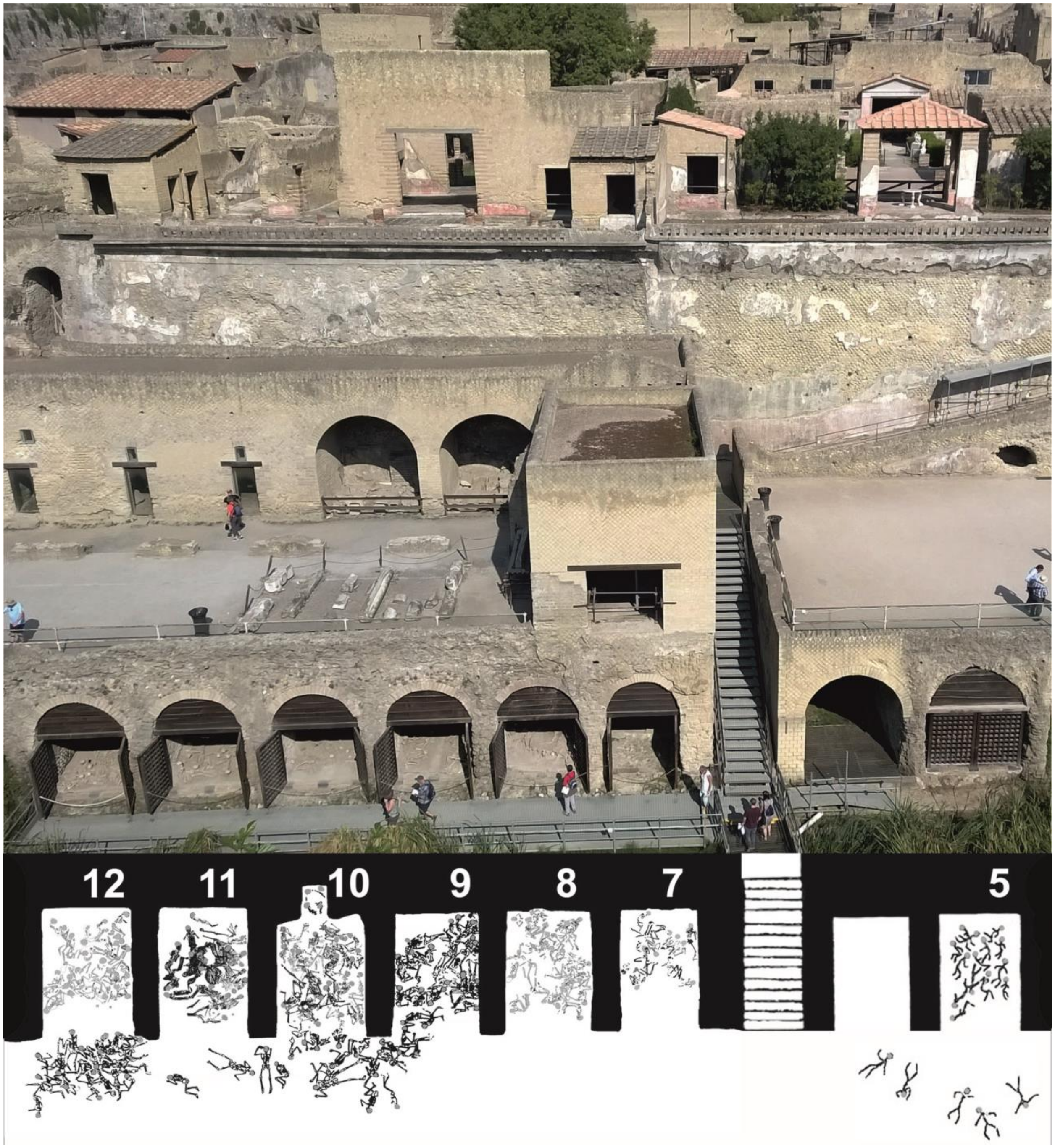

Fig. S29. View of the sea-front chambers on the beach of Herculaneum. The graphic represents the rooms crowded by human victims as they were found during the archaeological investigations in the 1980s and 1990s. The analysed bone samples belong to some of the victims' skeletons discovered in chambers 5 and 10.

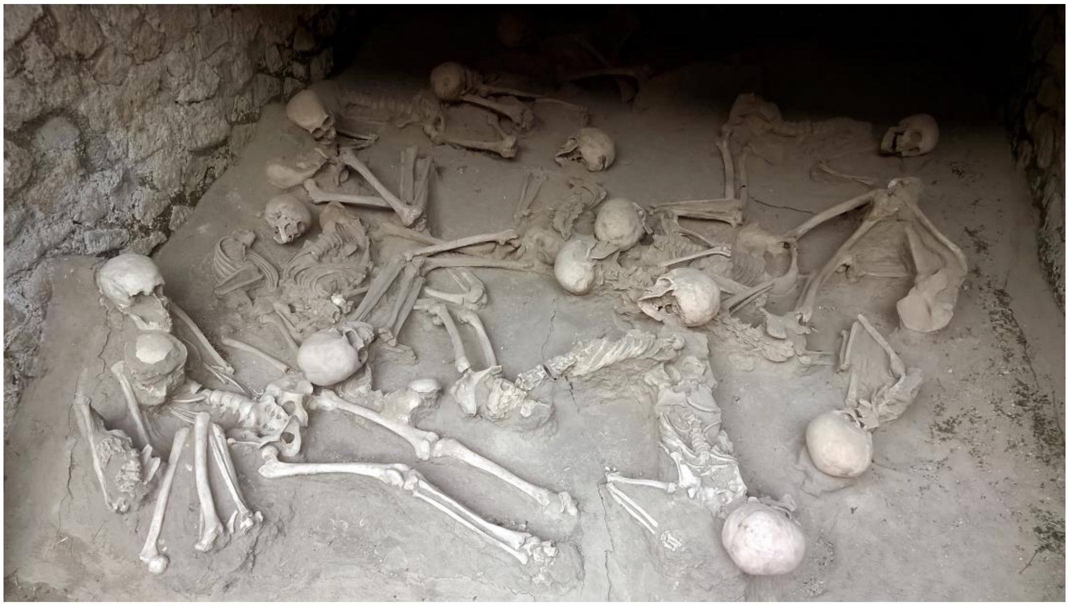

Fig. S30. Fiberglass replica of the Herculaneum victims in chamber 11. The casts were realized before removing the skeletons, in order to preserve the evidence of the masse catastrophe.

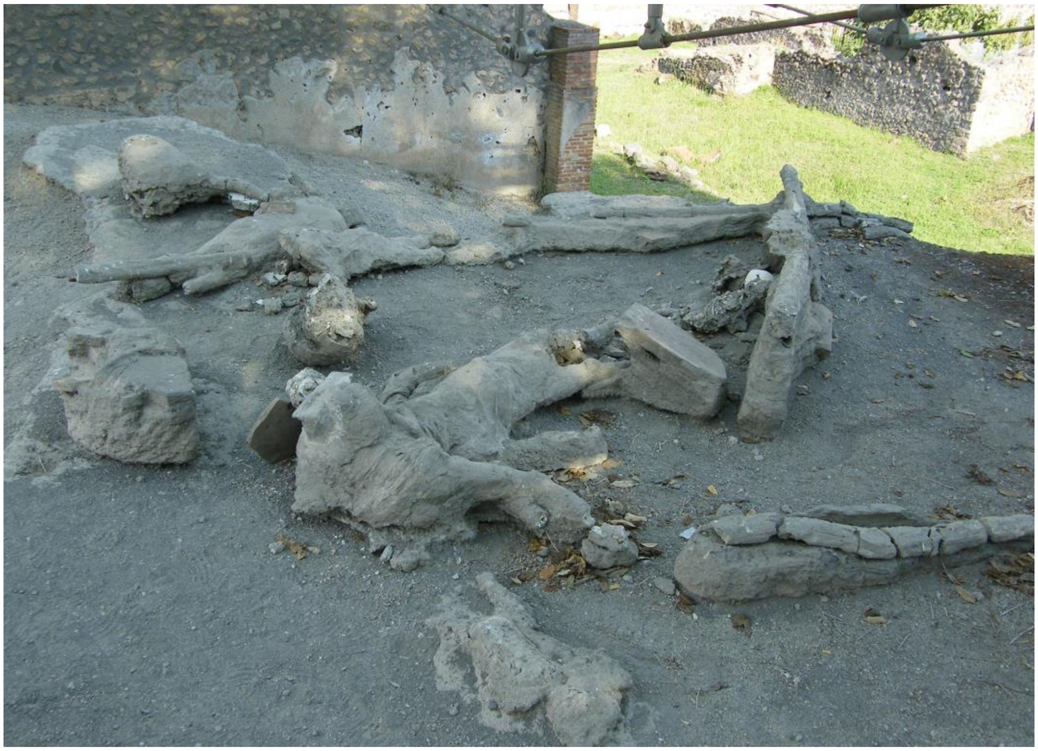

Fig. S31. Plaster casts of human victims found in Pompeii. The victims were found in 1990 in an open area, close to the House of *Stabianus* (*insula* 20, *regio* I).
